# Multiplex amplicon sequencing for microbe identification in community-based culture collections

**DOI:** 10.1038/srep29543

**Published:** 2016-07-12

**Authors:** Jaderson Silveira Leite Armanhi, Rafael Soares Correa de Souza, Laura Migliorini de Araújo, Vagner Katsumi Okura, Piotr Mieczkowski, Juan Imperial, Paulo Arruda

**Affiliations:** 1 Centro de Biologia Molecular e Engenharia Genética, Universidade Estadual de Campinas (UNICAMP), 13083-875, Campinas, SP, Brazil; 2 Department of Genetics, University of North Carolina, Chapel Hill, North Carolina, USA; 3 Centro de Biotecnología y Genómica de Plantas, Universidad Politécnica de Madrid (UPM) – Instituto Nacional de Investigación y Tecnología Agraria y Alimentaria (INIA), Campus Montegancedo UPM, 28223, Pozuelo de Alarcón, (Madrid), Spain; 4 Consejo Superior de Investigaciones Científicas, Madrid, Spain; 5 Departamento de Genética e Evolução, Instituto de Biologia, Universidade Estadual de Campinas (UNICAMP), 13083-970, Campinas, SP, Brazil

## Abstract

Microbiome analysis using metagenomic sequencing has revealed a vast microbial diversity associated with plants. Identifying the molecular functions associated with microbiome-plant interaction is a significant challenge concerning the development of microbiome-derived technologies applied to agriculture. An alternative to accelerate the discovery of the microbiome benefits to plants is to construct microbial culture collections concomitant with accessing microbial community structure and abundance. However, traditional methods of isolation, cultivation, and identification of microbes are time-consuming and expensive. Here we describe a method for identification of microbes in culture collections constructed by picking colonies from primary platings that may contain single or multiple microorganisms, which we named community-based culture collections (CBC). A multiplexing 16S rRNA gene amplicon sequencing based on two-step PCR amplifications with tagged primers for plates, rows, and columns allowed the identification of the microbial composition regardless if the well contains single or multiple microorganisms. The multiplexing system enables pooling amplicons into a single tube. The sequencing performed on the PacBio platform led to recovery near-full-length 16S rRNA gene sequences allowing accurate identification of microorganism composition in each plate well. Cross-referencing with plant microbiome structure and abundance allowed the estimation of diversity and abundance representation of microorganism in the CBC.

Advances in metagenomic sequencing methods have linked the vast microbial diversity to assorted biological functions in human health, plant development, and biotechnological processes^1^,^2^. In plants, beneficial microorganisms can affect plant growth by playing vital roles in several mechanisms, such as repression of pathogens, production of phytohormones, nitrogen fixation, stress resistance and nutrient uptake^3^,^4^,^5^,^6^,^7^,^8^,^9^,^10^. However, evaluation of the biological potential of microorganisms might be limited to the availability of representatives microbial culture collections^11^. Additionally, common approaches for microorganism isolation have limited throughput capabilities and lack the ability to cross-reference with culture-independent surveys.

Isolation, cultivation and identification of microbial candidates are imperative steps for the emerging areas of microbial community manipulation and discovery of its biological function. However, we still rely on approaches from the past century for recovery of microbial communities. Traditionally, the isolation of a representative set of microorganisms from a given environment requires several rounds of picking and streaking to obtain pure colonies. These methods are time-consuming, costly and may not be appropriate to the recovery of representative samples of the microbial diversity^12^. Microbial collections based exclusively on axenic cultures may result in the loss of relevant biological information since there are microorganisms that might depend on microbe-microbe interactions for their growth^10^,^13^. In such cases, the strict mutual dependence within microbes may drastically reduce their growth in culture media thus lessen the chance that it would be represented in collections obtained using pure colonies pickings. To this point, there is a need for new methods to isolate microbes, store them and annotate their identification in a high-throughput manner, preserving potential microbe-microbe interactions that is lost when culture collections are constructed based on axenic colonies.

Although technologies for microbial surveys have rapidly evolved, the same did not occur for methods of isolation and identification of microbial communities. This impairment between microbial surveys and isolation procedures constitute a barrier for microbiome research imposed by the lack of methods for systematically store microbiota culture collections^11^,^12^,^14^. Also, it is important to have the possibility to cross-reference culture collections with culture-independent community analysis which is an important step to explore fundamental aspects of microbial studies such as estimate microbial recovery of isolation procedures, perform functional screenings and study host-microbe interactions^11^.

Here we advance the concept of community-based culture collections (CBC) that is based on the isolation of colonies containing single or multiple microorganisms, and store them in a single plate well. The rationale is to bypass the laborious work of pure culture isolation and to concentrate on relevant insights based on community analysis, microbe-microbe and microbe-host interactions. The CBC approach may be useful in case of need to recover larger fractions of microbiota from a given environment to preserve putative microbe-microbe interactions. If microbes present in a given plate well appear to be of interest, pure colonies can be easily obtained, or their interactions further studied. Similar methods based on isolation of communities instead of axenic colonies have recently been reported^15^. We also presented a method to annotate the CBC through a multiplex strategy for pooling and sequencing near-full-length 16S ribosomal gene amplicons. This approach allows precise identification of CBC microorganisms that can be directly compared with culture-independent community analysis for the estimation of microbial community recovery and selection of microbial candidates. The method presented here is suitable for the construction of culture collections from any environment in a high-throughput and large-scale manner.

## Results and Discussion

### Community-based culture collection (CBC) of sugarcane

The method described here was developed when preparing a sugarcane CBC. The sugarcane CBC was prepared by picking colonies from primary platings of enriched microbial fractions of the rhizosphere, endophytic root and endophytic stalk of developing plants. Microbial samples were diluted and plated on three different culture media supplemented with sugarcane syrup. Colonies were picked regardless if they were single or multiple (community) microorganisms. A total of 5,137 colony communities were picked and stored in 96-well plates.

### Multiplex amplicon sequencing for microbe identification

**Figure 1:**
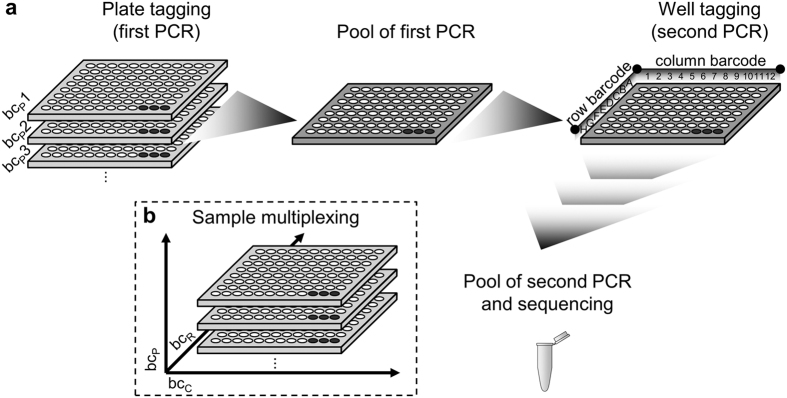
Schematic representation of multiplex strategy for 16S rRNA gene amplification of community-based culture collection (CBC). The multiplex strategy allows pooling of libraries from more than one 96-well plate in one tube for sequencing of the near-full-length gene using a single run of the PacBio RS platform. (**a**) A two-step PCR for library preparation. The first step adds unique plate barcodes and the second step, rows and columns barcodes. (**b**) The combination of plates, rows and columns barcodes allows tracing back the exact well of origin from each sequence. Highlighted wells are kept empty and allow us to use them as positive (H10) and negative (H11 and H12) controls during PCR reactions and libraries preparation. bc_P_: plate barcode. bc_R_: row barcode. bc_C_: column barcode.

In order to identify and annotate the CBC near-full-length microbial 16S rRNA amplicons were sequenced from 96-well plates pooled down to a single tube using the PacBio RS sequencing platform (Fig. 1a). The use of longer 16S sequences enhances the quality of biological information, by either improving the depth of taxonomic assignment^16^,^17^ and comparison with available full-length 16S rRNA databases^18^,^19^. Two-step PCR amplifications were designed to add individual barcodes for the identification of each plate and well using primers for the 16S rRNA gene based on the V3–V9 region (Supplementary Fig. 1a). The first PCR step amplifies the 16S rRNA gene and adds specific barcodes for each 96-well plate (Supplementary Fig. 1b). First step PCR-barcoded amplicons were pooled in a single 96-well plate and used as templates for the second PCR step, which adds specific barcodes for columns and rows (Supplementary Fig. 1b). The second PCR-barcoded amplicons were pooled into a single tube. The pooled amplicons were sequenced on the PacBio RS platform because it conveniently allows the retrieval of the near-full-length sequence of the 16S rRNA genes. The multi-tagging method (Fig. 1b), coupled to PacBio SMRT sequencing, allows tracing back individual sequences to their original wells regardless of whether the well contains one or multiple microorganisms. The method was developed using five 96-well plates from the sugarcane CBC. A bioinformatics pipeline was established for the sequence demultiplexing, removal of chimeric and nonspecific sequences, clustering into operational taxonomic units (OTUs) and their taxonomical assignment (Supplementary Fig. 2).

### Circular consensus sequence (CCS) accuracy and OTU assignment

The intrinsically low raw-read quality of PacBio was overcome by generating circular consensus sequences (CCSs). Since errors are randomly distributed along raw sequences, error rates are almost eliminated by the CCS processing, and the higher the coverage of CCSs, the higher their accuracy^20^,^21^,^22^. Data accuracy was evaluated by assembling CCSs using a minimum value of 2× coverage. The per-base error rates of assembled CCSs were assessed by comparing CCSs generated from positive control wells that contained 16S rRNA amplicons from *E. coli* to a 16S reference sequence from this organism (Supplementary Fig. 3a). The average per-base error rate was 2.3% for CCSs with coverage lower than 5×, 0.7% for coverage from 5 to 9×, and 0.3% for CCSs with coverage higher than 9× (Supplementary Fig. 3b). Notably, 74.7% of the dataset was constituted by CCSs with coverage greater than 9× (Supplementary Fig. 4a) and showed the expected amplicon lengths (Supplementary Fig. 4b).

**Figure 2:**
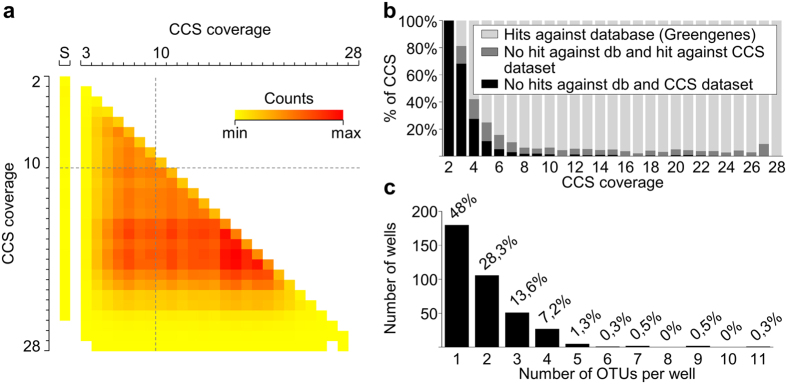
Sequence reliability filter maximizes retention of OTUs that might represent true biological sequences in community-based isolates. A quality filter solely based on CCS coverage causes loss of sequences that contain relevant biological information (Supplementary Fig. 5). A low-coverage CCS can be validated regarding its reliability if it has a representative in a 16S rRNA gene database or if it has high similarity to at least one other sequence in the CCS dataset from the same culture collection. (**a**) Heatmap showing that low-coverage CCSs (<10×) mostly cluster with high-coverage CCSs (≥10×) in the OTU clustering, which raises their reliability. (**b**) Alignment of CCSs against Greengenes^18^ 16S rRNA gene database and the CCS dataset from the culture collection. The lower the coverage, the higher the number of error-prone CCSs (no hit against Greengenes and no hit against CCS dataset). (**c**) Number of OTUs per well found after removing error-prone CCSs. S: singletons. db: database.

We then investigated if the low-coverage CCSs could lead to a misidentification or produce false positive OTUs. The assumption was that OTUs called by high-coverage assembled CCSs have a low probability of being false positives because of their high sequence accuracy. Low-coverage CCSs have a greater chance of containing more errors and therefore could lead to singleton OTUs that do not represent true biological sequences. We first addressed this question by looking for sequence similarity through pairwise alignment of CCSs. Based on quality accuracy, CCSs were arbitrarily classified as low- (<10×) and high-coverage (≥10×). We found that the majority of low-coverage CCSs align with at least one high-coverage CCS, which raises their reliability and lowers the chance that they represent false and non-biological sequences (Fig. 2a). We investigated if it directly reflects in OTU clustering by looking how many OTUs were formed by the clustering of high-coverage CCS, low-coverage CCS or a combination of both. A total of 361 out of 1,003 identified OTUs presented at least one low-coverage CCS clustered with high-coverage CCSs. Hence, they could more confidently be assigned as true OTUs. Among 558 singleton OTUs, 335 were called from one low-coverage CCS and 223 from one high-coverage CCS (Supplementary Fig. 5).

### Reliability filter for removal of error-prone sequences

Traditionally, quality filters based on CCS coverage are used to obtain highly accurate sequences. However, our analysis indicates that removing sequences based on coverage is not an adequate method to remove error-prone CCSs (Fig. 2b) because it could potentially lead to the loss of relevant biological information. The trade-off between accuracy and throughput should be determined for each application. In addition, considering that preserving information is highly desirable, we elaborated a strategy to maximize the number of sequences with high confidence to represent true biological sequences (reliable sequences). The rationale involved filtering the data based on the reliability of the CCS, rather than solely on its coverage. A CCS could be considered reliable if (1) it is above a threshold of similarity to a sequence deposited in a curated database and/or (2) if it is above a threshold of similarity to any other sequence within the dataset. The first case comprised instances in which the microorganism was already identified and had its gene sequences deposited in a database. The second one assumed that within the collection there might exist redundancy, i.e. more than one well having the same microbe. Thus, the fact that a sequence appeared more than once in the collection, especially if called by high-coverage CCSs, makes it reliable.

After sequence demultiplexing, all chimeric, non-specific and larger than expected sequences were removed (Table 1). Out of 11,750 demultiplexed CCSs, a total of 12.3% and 0.14% represented chimeras and nonspecific sequences, respectively (Table 1). From the remaining CCSs (usable CCS) we were able to recover a total of 375 wells out of the expected 480 (78.1%; Table 1). Unrecovered wells might have occurred due to the lack of microorganism growth, low DNA concentration or low primer specificity. Usable CCSs were filtered by reliability (Supplementary Fig. 6a), and a total of 312 CCSs were discarded after being classified as error-prone (Table 1). The reliability filter allowed us to retain 22,6% of the total usable CCSs that would have been discarded if a filter based on coverage had been applied (Table 1 and Supplementary Fig. 6b). As isolates in the sugarcane CBC might contain multiple microorganisms, the gain in the number of CCSs per well is advantageous for identification of microbes and also for further community analysis.

### Microbial identification and cross-reference with culture-independent community analysis

We next evaluated the number of identified OTUs per well. Over half of the wells showed two or more OTUs (Fig. 2c). The high number of wells that contained more than one identified microorganism can be considered as a proof of the concept for the method described in this work (Supplementary Fig. 7 and Supplementary Table 1). Considering the identification at the genus level with a confidence score of >0.95 given by UTAX (www.drive5.com/usearch/manual/utax_algo.html) we counted 34 distinct genera in the five plates tested (Supplementary Table 2). Together, the four most representative genera (*Bacillus*, *Chitinophaga*, *Rhizobium* and *Burkholderia*) accounted for over 56% of the total OTUs assigned in the five plates. A total of 206 OTUs could not be assigned at the genus level.

**Figure 3:**
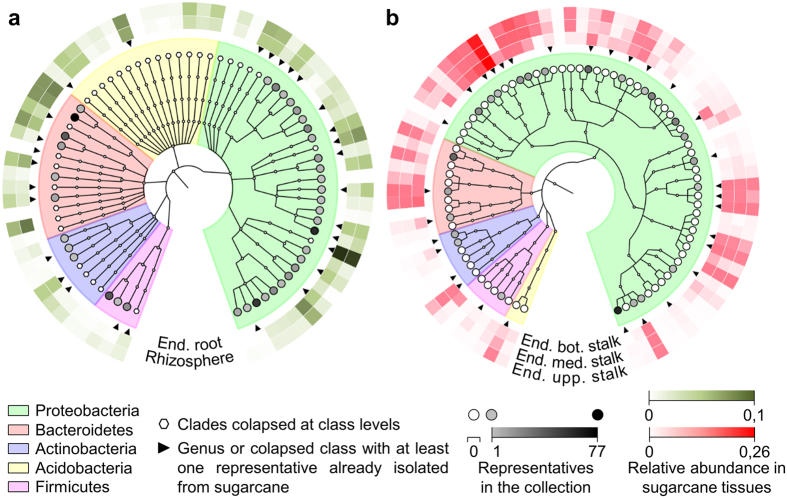
Cross-referencing of community-based culture collection (culture-dependent) and sugarcane microbial profile (culture-independent). The sugarcane community profile has identified a core bacterial community comprised of less than 20% of the total microbial richness but accounting for over 90% of the total microbial relative abundance^23^ (Supplementary Note). Because of its relevance, we have highlighted the recovery for the core microbiome. OTUs in five culture collection plates were cross-referenced against core community profile from root (rhizosphere and endophytic root) and stalks (endophytic bottom, medium and superior region of stalks) from sugarcane plants. The identified isolates comprised OTUs that account for 14.2% and 29.1% of the total bacterial relative abundance in sugarcane roots and endophytic stalk, respectively. Cladograms were constructed at the genus level based on taxonomy assignment. (**a**) Endophytic root and rhizosphere microbiota. The cladogram was simplified to show only five phyla; genera with no representatives in the collection were collapsed (see Supplementary Fig. 8 for detailed information). (**b**) Endophytic stalk microbiota. End.: endophytic. Bot.: bottom. Med.: medium. Upp.: upper.

**Figure 4:**
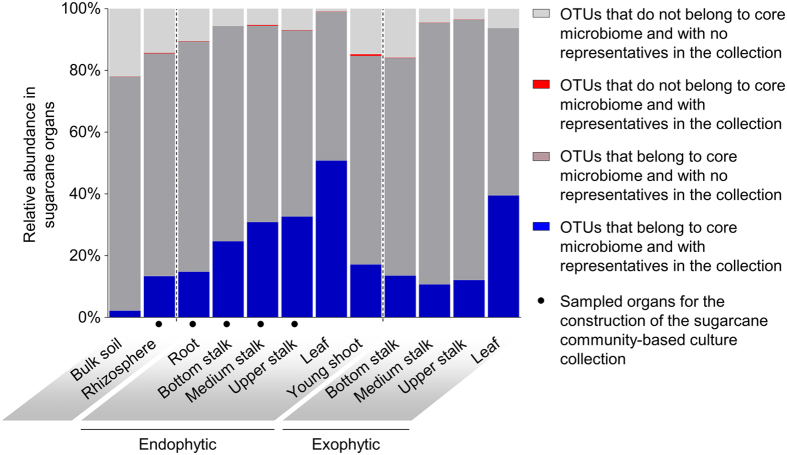
Estimation of microbial community recovery in community-based culture collection (CBC). The graph shows an estimate of the relative abundance represented by the isolates in five plates of the CBC. The estimate was calculated based on cross-reference between the OTUs from CBC and sugarcane microbial profile^23^ (Supplementary Note). The taxonomical overlap of plant organs made possible to find representatives of the microbial community from plant organs that were not sampled for construction of the sugarcane CBC.

Finally, we estimated the extent of the sugarcane microbiota diversity that we recovered using the described method. The OTUs identified in the five CBC plates were cross-referenced with the 20,731 OTUs from the sugarcane microbiota previously identified by culture-independent community analysis^23^ (Supplementary Note). The cross-referencing was performed by sequence alignment using USEARCH^24^ with a threshold of 97% identity. This analysis revealed that the sugarcane CBC contains members of the most abundant microbial genera of the root (Fig. 3a and Supplementary Fig. 8) and endophytic stalk sugarcane organ core microbiomes^23^ (Fig. 3b). Identified isolates comprised OTUs that account for 13.3%, 14.8% and 29.1% of the total bacterial relative abundance in sugarcane rhizosphere, endophytic root and endophytic stalk, respectively (Fig. 4). Although we aimed to isolate root and stalk endophytic microorganisms, our collection contained microorganisms that represent 12.2%, 39.5% and 50.8% of the exophytic stalk, exophytic leaf and endophytic leaf diversity, respectively (Fig. 4). Furthermore, most of the isolates represent groups of bacteria that have never been studied regarding their biological role in association with plants (Fig. 3a,b and Supplementary Fig. 8).

## Conclusions

The biotechnological use of environmental microbiota requires methods for construction and identification of large microbial culture collections. Conventional techniques for isolation and identification of microorganisms are based on axenic cultures obtained by several steps of picking and streaking colonies. These methods are hardly scalable for massive isolation of microorganisms and do not allow cross-referencing with data from culture-independent community analysis. Here we presented an alternative for high-throughput identification of microorganisms in a culture collection constructed by colony picking of primary platings. These colonies may contain single of multiple microorganisms, and for this reason it was called community-based culture collection (CBC). The identity of the microorganisms in each well was assigned using multiplex sequencing of amplicons that allowed tagging plates and wells. The generated data could be cross-referenced with culture-independent community analysis to estimate precisely the microbial recovery. The method can be used for annotation of culture collections of any environment as it overcomes the major limitation of current microbiome research related to microbiota culture collections.

## Material and Methods

### Construction of a sugarcane community-based culture collection

The microorganisms used in this study represent a sample of a sugarcane community-based culture collection (CBC). This culture collection was constructed from microorganism-enriched pellets prepared from the rhizosphere, endophytic root and endophytic stalk compartments^23^. Briefly, microorganism-enriched pellets were diluted and plated on LB medium containing sugarcane syrup and incubated at 30 °C for 2–4 days. Individualized colonies from primary platings were picked in 96-well plates that contained liquid LB plus sugarcane syrup medium. From the 96-well plates, 93 wells were used for colony picking, and H10, H11, and H12 wells were used for *E. coli* controls. The 96-well plates were shaken at 225 rpm at 30 °C for 2–4 days and stored at −80 °C. No previous assumption was made concerning whether each of the 93 wells contained pure or multiple microorganisms.

### DNA extraction

Replicas of five 96-well plates from the sugarcane CBC were thawed, centrifuged at 3,200 × *g* for 10 min at 15 °C, and the supernatant was discarded. The bacterial pellets were resuspended in 200 μL of GTE (glucose 50 mM, Tris-HCl 25 mM pH 8 and EDTA 10 mM pH 8). Plates were centrifuged again at 3,200 × *g* for 10 min at 15 °C, and the supernatant was discarded. Pellets were resuspended in 60 μL of PrepMan Ultra Sample Preparation Reagent (Applied Biosystems, Foster City, CA, USA) and water at a 1:2 reagent-to-water ratio. The DNA extraction was performed according to the manufacturer’s instruction protocol. DNAs were stored in 96-well plates at −20 °C.

### Primer design

The 16S rRNA V3–V9 gene region (Supplementary Fig. 1a) was amplified using 341f^25^ and 1492r^26^ primers. For the first PCR step, plate barcodes were added to the 1492r primer (first PCR; Supplementary Fig. 1b). Primers for the second step were designed with MT-FS region and Illumina barcode sequences (Nextera sequences) for columns and rows (second PCR; Supplementary Fig. 1b). A two-step library preparation is a standard protocol used for 16S community analysis and provides reliable sequences^27^. Additionally, we used Illumina sequences (Nextera sequences) to give flexibility regarding sequencing platform. Using these primers, amplicons are compatible with both Illumina SBS chemistry and PacBio platform. Both the first and second PCR forward and reverse primers are listed in the Supplementary Data 1. All of the primers were obtained from Integrated DNA Technologies (IDT, Coralville, IA, USA).

### First PCR step

PCR reactions were performed in 96-well plates (Fig. 1a). The reaction was designed to amplify the 16S rRNA V3–V9 gene region with unique plate barcodes. The reverse primer contained an overhang with a 9-bp barcode sequence (Supplementary Fig. 1b). To minimize the pipetting variation of small volumes, master mixes were prepared for 100 reactions. The master mix was prepared using the KAPA2G Robust PCR Kit (KK5024; Kapa Biosystems, Wilmington, MA, USA) and contained 400 μL of 5× KAPA2G buffer A, 400 μL of 5× KAPA enhancer, 100 μL of 10 μM forward primer, 100 μL of 10 μM reverse-tagging primer (plate tagging), 40 μL of 10 μM dNTPs, 8 μL of KAPA2G Robust DNA polymerase and 752 μL of sterile deionized water. 18 μL of master mix and 2 μL of DNA template were dispensed into each well of the five 96 well plates. Each plate contained *E. coli* K-12 MG1655 purified DNA as positive controls. The amplification program was as follows: 5 min denaturation at 95 °C; 25 cycles of 30 s denaturation at 95 °C, 30 s primer annealing at 55 °C, 2 min extension at 72 °C; and a final cooling to 4 °C. The reactions were validated for amplification quality in 1% agarose gel electrophoresis.

### Pooling and purification of the first-PCR amplicons

The amplicons of the five 96-well plates were pooled by transferring, with a multichannel pipette, 2 μL of each well of first-PCR reactions into a single 96-well plate (Fig. 1a). The final 10-μL volume was purified with Agencourt AMPure XP Beads (Beckman Coulter, Brea, CA, USA) according to the manufacturer’s instruction, at a bead-to-DNA ratio of 0.6:1. Purified PCR products were resuspended in 30 μL of sterile deionized water and stored at −20 °C.

### Second PCR step

The pooled first-PCR amplicons were used as templates for the second PCR step to add unique barcodes for rows and columns in a 96-well plate format (Fig. 1a). The PCR reactions were performed using forward and reverse primers from Nextera XT DNA Library Prep Kit (FC-121-1012; Illumina, San Diego, CA, USA). An overhang with 8-bp barcodes was added to the forward primer for each one of the 8 rows of the 96-well plate. For the reverse primer, an overhang with 8-bp barcodes was added for each one of the 12 columns of the 96-well plate (Fig. 1a). The PCR amplification was performed using the KAPA HiFi HotStart ReadyMix PCR Kit (KK2602; Kapa Biosystems, Wilmington, MA, USA) in a 20-μL reaction containing 10 μL 2× KAPA HiFi HotStart ReadyMix, 2 μL of 10 μM forward-tagging primer, 2 μL of 10 μM reverse-tagging primer and 6 μL of the first-PCR amplicons. The amplification program was as follows: 3 min denaturation at 95 °C; 12 cycles of 30 s denaturation at 95 °C, 30 s primer annealing at 55 °C, 30 s extension at 72 °C; and a final cooling to 4 °C. Amplicons were purified using 12 μL of Agencourt AMPure XP Beads (Beckman Coulter, Brea, CA, USA) according to the manufacturer’s instructions at a bead-to-DNA ratio of 0.6:1. Exonucleases were inactivated by heating the plate in a thermocycler at 65 °C for 30 min. The amplified products were validated by 1% agarose gel electrophoresis.

### Quantification, library preparation, and sequencing

The cleaned amplicons of the second-step PCR were quantified using Qubit dsDNA BR Assay Kit (Invitrogen, Carlsbad, CA, USA) and pooled at equimolar ratios in a single tube (Fig. 1a). The pool of the second-PCR products was quantified using Qubit and validated for quality by 1% agarose gel electrophoresis. The PCR products were prepared for PacBio circular consensus sequencing using the “2-kb Template Preparation and Sequencing” protocol from Pacific Biosciences (PacBio, Menlo Park, CA, USA). In this protocol, the PCR products are circularized by ligation of SMRTbell adapters and repeatedly sequenced by multiple passes of the polymerase. All library preparation steps were performed at the University of North Carolina (UNC) High-Throughput Sequencing Facility (HTSF, Chapel Hill, North Carolina, USA). The library was sequenced using the PacBio RS II platform (P5-C3 chemistry).

### Data processing

The raw sequence data generated by the PacBio RS II sequencing was assembled in circular consensus sequences (CCSs) using the RS_ReadsOfInsert protocol from the PacBio’s SMRT Portal v2.1.1 and the parameters of “minFullPasses 2” and “minPredictedAccuracy 90”. The read coverage and length distributions are shown in Supplementary Fig. 4a,b, respectively. Data processing was automated using in-house implemented scripts. The scripts used for data processing are available in the GitHub repository (https://github.com/saccharome/CBC). The data analysis considered only CCSs with at least one hit against the Greengenes^18^ high-quality full-length 16S rRNA sequences database (May 2013 release) and/or the CCS dataset. CCSs that did not meet these criteria were considered error-prone and were discarded.

### CCS coverage

The CCSs coverage was calculated as the sum of nucleotides of all subreads related to the CCS (i.e. after removing SMRTbell adapters from raw sequences) divided by the length of the assembled CCS. Numbers were rounded to the nearest integer value.

### Demultiplexing

CCSs were demultiplexed according to their PCR barcodes. The UBLAST algorithm (www.drive5.com/usearch) was used to identify sequences of both forward and reverse Nextera transposase and non-barcoded 1492r primer sequences (14, 15 and 16 nucleotides, respectively). Based on their location, the positions of barcodes were determined. The processing pipeline accepted a maximum of 3 mismatches for the alignments. The high stringency was chosen to ensure the correct traceability of sequences, and CCSs with at least one unassigned barcode were discarded. Barcodes identified on their predicted positions were considered consistent. The “search_oligodb” program was used to assign the barcodes with no mismatch. Both programs used are from the software package USEARCH^24^.

### Sequence reliability filtering

CCSs were aligned to the Greengenes^18^ database using “usearch_global” from USEARCH^23^ software package with a 97% identity threshold (Supplementary Fig. 6a). CCSs with no hit against the database were then aligned to the original CCS dataset using the same parameters. CCSs with no hit against both databases were considered error-prone and discarded.

### Chimera and nonspecific sequences removal

Sequences that contained fragments from different templates were considered chimeras. Chimeras were removed using the UCHIME^28^ program against a high-quality chimera-free reference database (www.drive5.com/uchime/rdp_gold.fa). CCSs larger than expected (>1,600 base pairs) were also discarded as they may also represent chimeras^21^. The dataset that contained cleaned sequences was aligned to the most recent version of the Greengenes^18^ database using the “usearch_global” program from the USEARCH^24^ software package. Sequences with no hit or identity lower than 75% were discarded.

### Clustering

CCS sequences were clustered within each well individually. Clustering was performed with a 97% identity threshold using UPARSE^29^ pipeline in a Perl script to automate the process. OTUs from this step were used for taxonomical classification, cross-referencing of culture collection with culture-independent community analysis and recovery estimate. In order to access the redundancy of microorganisms, OTUs were re-clustered at 97% identity in a second step, using the above-mentioned parameters.

### Cross-referencing of culture-dependent and -independent community analysis and recovery estimate

An adapted culture-independent community profile of sugarcane root, leaf, and stalks using V4 16S ribosomal gene^23^ (Supplementary Note) was used for cross-referencing between culture-dependent and -independent community analysis. Sequences were globally aligned against OTUs from culture-independent data using “usearch_global” from USEARCH^24^ software package and only hits with ≥97% sequence identity were considered. Recovery estimate was calculated by summing the relative abundance of OTUs from the culture-independent data that had at least one hit against a CCS from the culture collection. Cladograms and trees for comparison of culture-dependent and -independent data were constructed using GraPhlAn^30^.

### Taxonomical assignment

The taxonomy of OTUs was assigned using RDP classifier^31^ against the Greengenes^18^ database and UTAX (www.drive5.com/usearch/manual/utax_algo.html) against a trained RDP^19^ database (www.drive5.com/utax/rdp_16s.fa) because both algorithms provide confidence scores.

## Additional Information

**Accession code:** The raw sequence data was deposited in Sequence Read Archive (SRA) under the accession code SRP076522.

**How to cite this article**: Armanhi, J. S. L. *et al.* Multiplex amplicon sequencing for microbe identification in community-based culture collections. *Sci. Rep.* **6**, 29543; doi: 10.1038/srep29543 (2016).

## Supplementary Material

Supplementary Information

## Figures and Tables

**Table 1:** Number of assembled, demultiplexed and filtered CCSs based on CCSs coverage and CCS reliability.

Total number of assembled CCSs (≥2× coverage)	27,220
Demultiplexed CCSs	11,750
Non-demultiplexed CCSs	15,345
CCSs larger than expected (>1,600 bp)	125
Chimeric CCSs	1,444
Non-specific sequences	16
Usable CCSs before quality filtering	10,290
Recovered wells before quality filtering	377 of 480
Usable CCSs after filtering by coverage (≥10× coverage)	7,688
Recovered wells after filtering by coverage (≥10× coverage)	366 of 480
Usable CCSs after filtering by reliability	9,978
CCSs with hits against Greengenes database	9,469
CCSs with no hits against Greengenes and with hit against CCS dataset	509
Error-prone CCSs	312
Recovered wells after filtering by reliability	375 of 480
