# Supplementary material for: Multiplex amplicon sequencing for microbe identification in community-based culture collections
Source: Sci Rep. 2016 Jul 12;6:29543. doi: 10.1038/srep29543 (PMC4941570; doi:10.1038/srep29543)
Supplement: Supplementary Information [file srep29543-s1.pdf]

Multiplex amplicon sequencing for microbe identification in community-based culture collections

Jaderson Silveira Leite Armanhi[1,6], Rafael Soares Correa de Souza[1,6], Laura Migliorini de Araújo[1], Vagner Katsumi Okura[1], Piotr Mieczkowski[2], Juan Imperial[3,4] and Paulo Arruda[1,5*]

[1]Centro de Biologia Molecular e Engenharia Genética, Universidade Estadual de Campinas (UNICAMP), 13083-875, Campinas, SP, Brazil.

[2]Department of Genetics, University of North Carolina, Chapel Hill, North Carolina, USA.

[3]Centro de Biotecnología y Genómica de Plantas, Universidad Politécnica de Madrid (UPM) – Instituto Nacional de Investigación y Tecnología Agraria y Alimentaria (INIA), Campus Montegancedo UPM 28223 – Pozuelo de Alarcón (Madrid), Spain.

[4]Consejo Superior de Investigaciones Científicas, Madrid, Spain.

[5]Departamento de Genética e Evolução, Instituto de Biologia, Universidade Estadual de Campinas (UNICAMP), 13083-970, Campinas, SP, Brazil.

[6]These authors contributed equally to this work.

*Correspondence should be addressed to P.A. (parruda@unicamp.br).

Running title: Community-based culture collections

SUPPLEMENTARY FIGURES

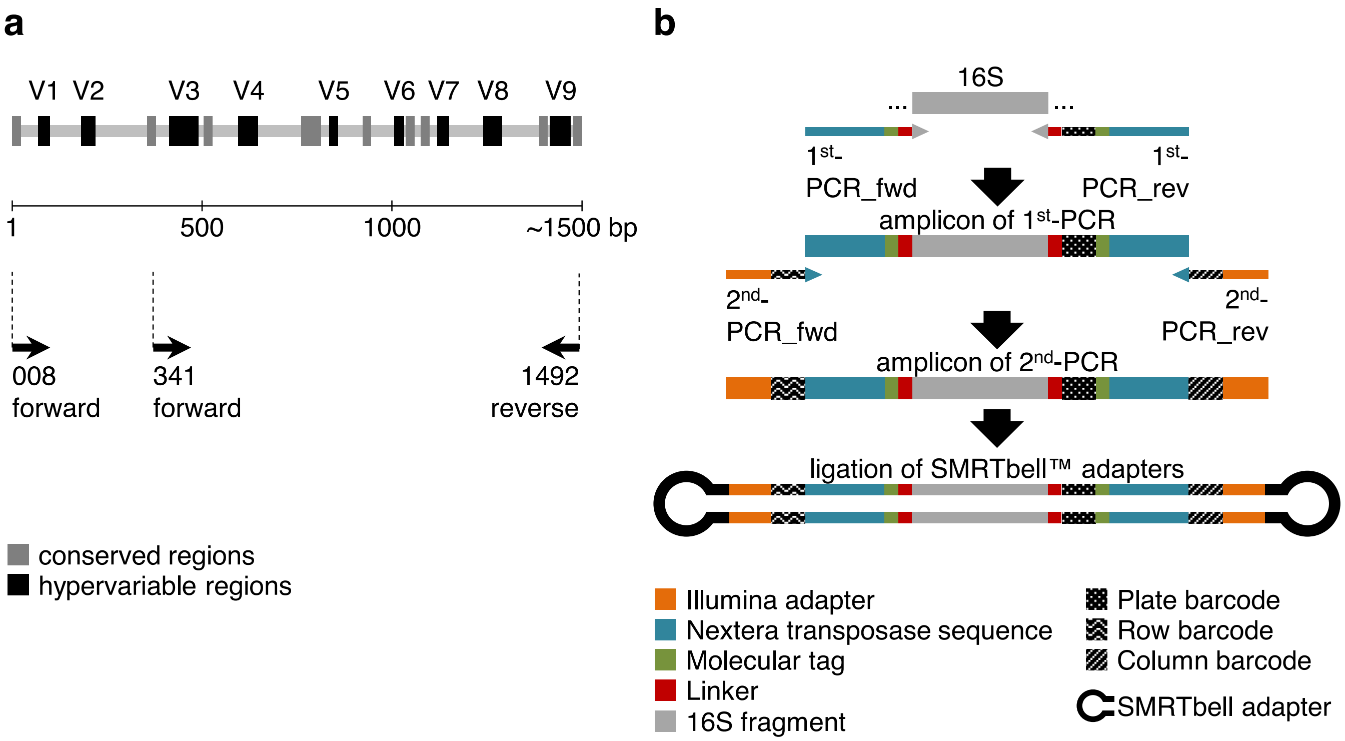

Supplementary Figure 1. Strategy for multiplexing and sequencing pools of samples in a 96-well plate configuration. (**a**) Schematic representation of a full-length 16S rRNA gene with nucleotide positions based on conventional *E. coli* numbering. Hypervariable regions and commonly used primers are shown. The method was initially established using 341f primer and extended to 008f primer for near-full-length sequencing of 16S rRNA gene. (**b**) Schematic representation of the amplification using a two-step PCR. The first-PCR step amplified the 16S region using the 1st-PCR_fwd and 1st-PCR_rev primers and added a plate barcode sequence. The second-PCR step amplified from the first-PCR amplicons using primers for Nextera transposase sequence and added row (2nd-PCR_fwd) and column (2nd-PCR_rev) barcodes. bp: base pair.

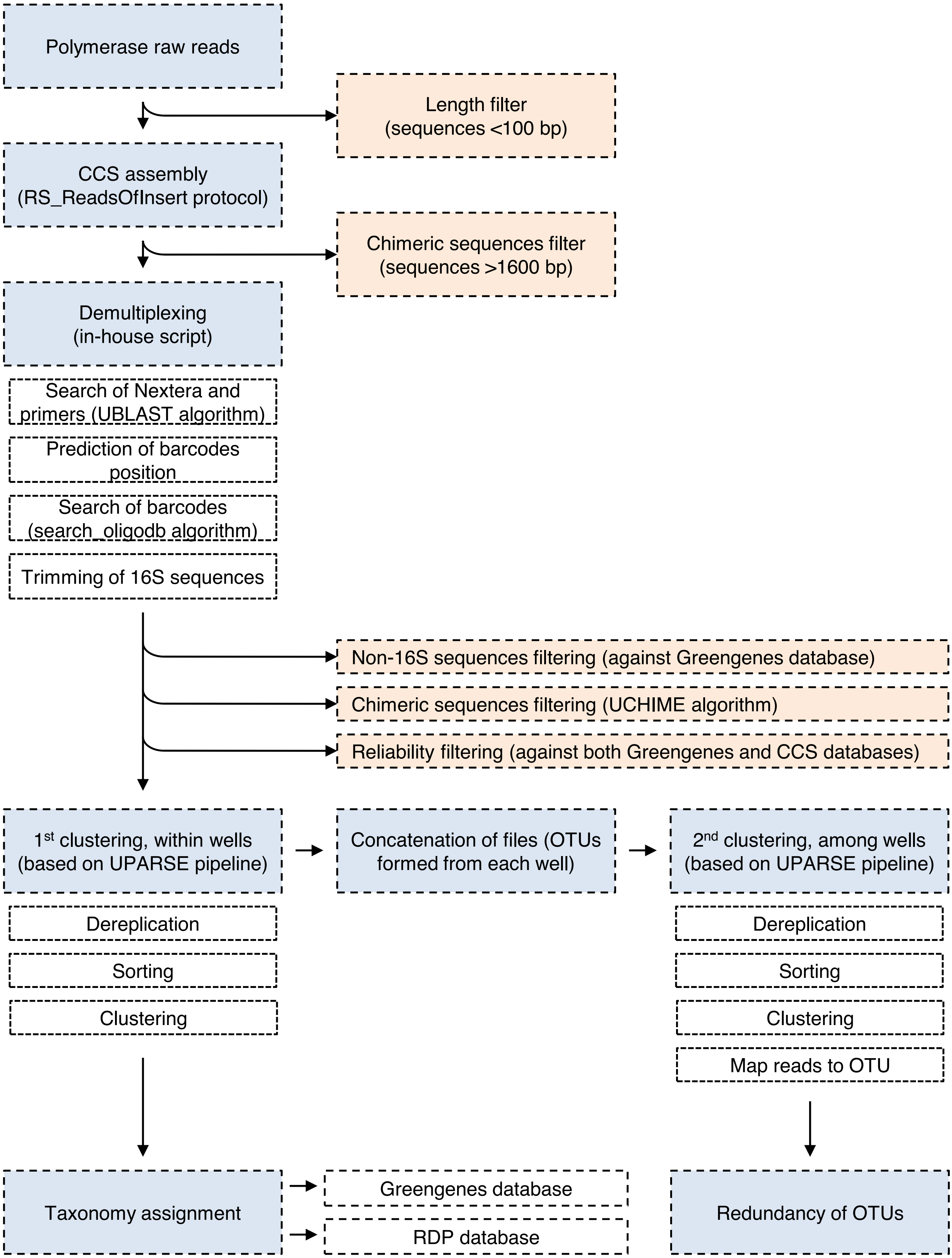

Supplementary Figure 2. Analysis pipeline. Raw reads were demultiplexed and filtered to ensure reliability and quality of CCSs. The first clustering step allows determination of which and how many microorganisms are present, while the second clustering step provides information on the redundancy of OTUs obtained from the first one.

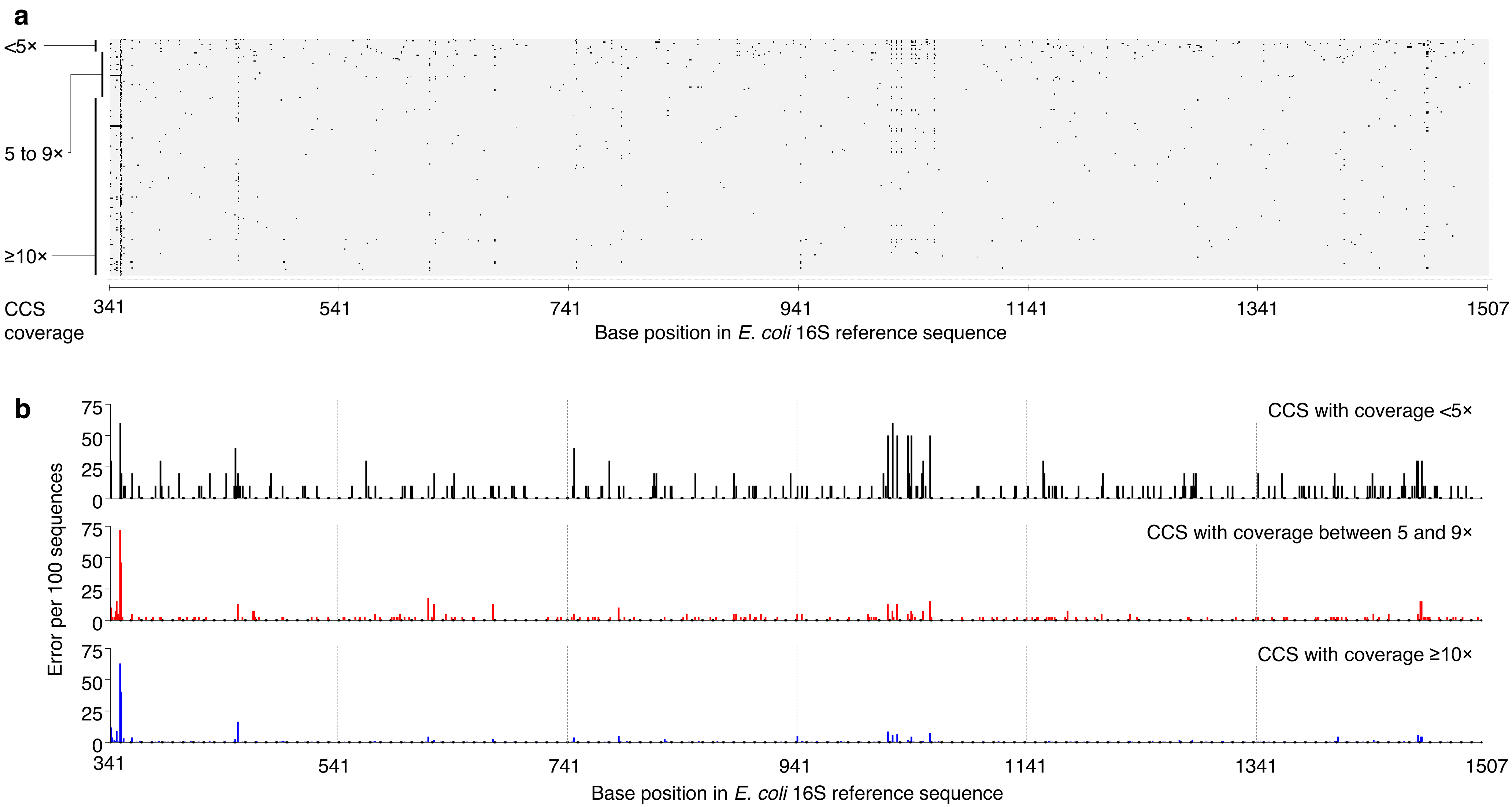

Supplementary Figure 3. Quality assessment of CCSs based on coverage. *E. coli* controls were included during library preparation. CCSs from control wells were aligned against a reference to validate the correlation of quality and CCS coverage. (**a**) Visual representation of per-base error of *E. coli* 16S rRNA CCSs compared to the corresponding gene sequences deposited in GenBank (gene ID 948466). CCSs were organized from lower (top) to higher (bottom) sequence coverage. Black dots indicate mismatches or gaps. (**b**) Percentage of per-base error rate. CCSs were grouped into three classes based on similarity of error rate (CCSs with coverage <5×, CCS with coverage between 5 and 9×, and CCS with coverage ≥10×).

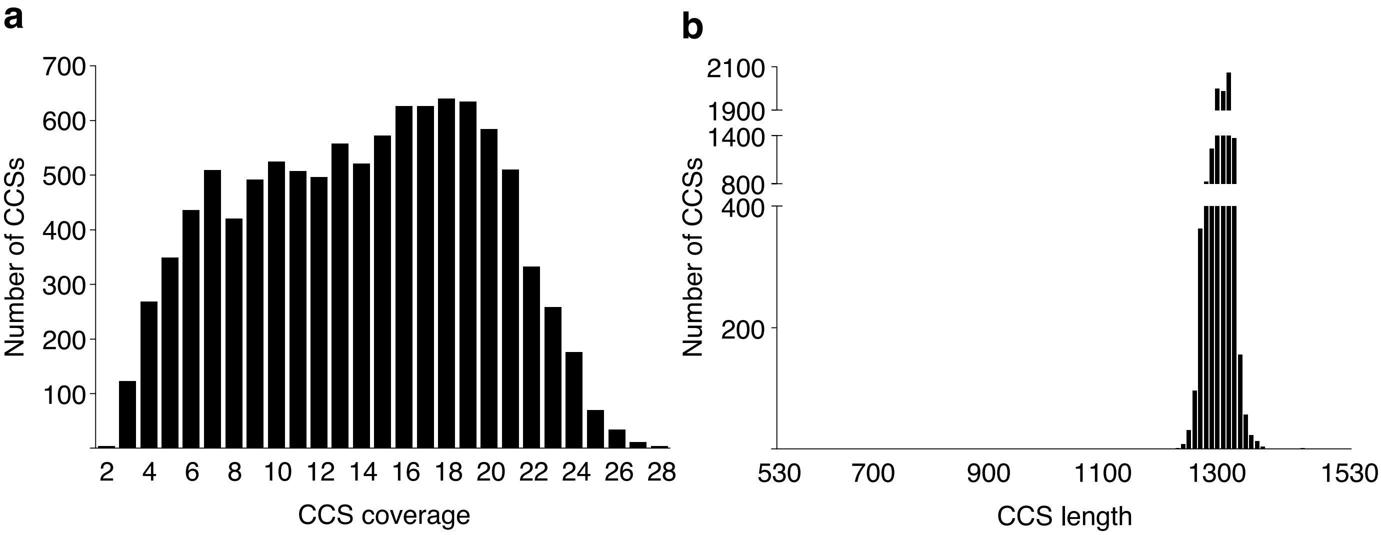

Supplementary Figure 4. Number of circular consensus sequences (CCSs) after demultiplexing, chimera and nonspecific sequences removal. (**a**) Number of CCSs as a function of coverage. (**b**) Number of CCSs as a function of length.

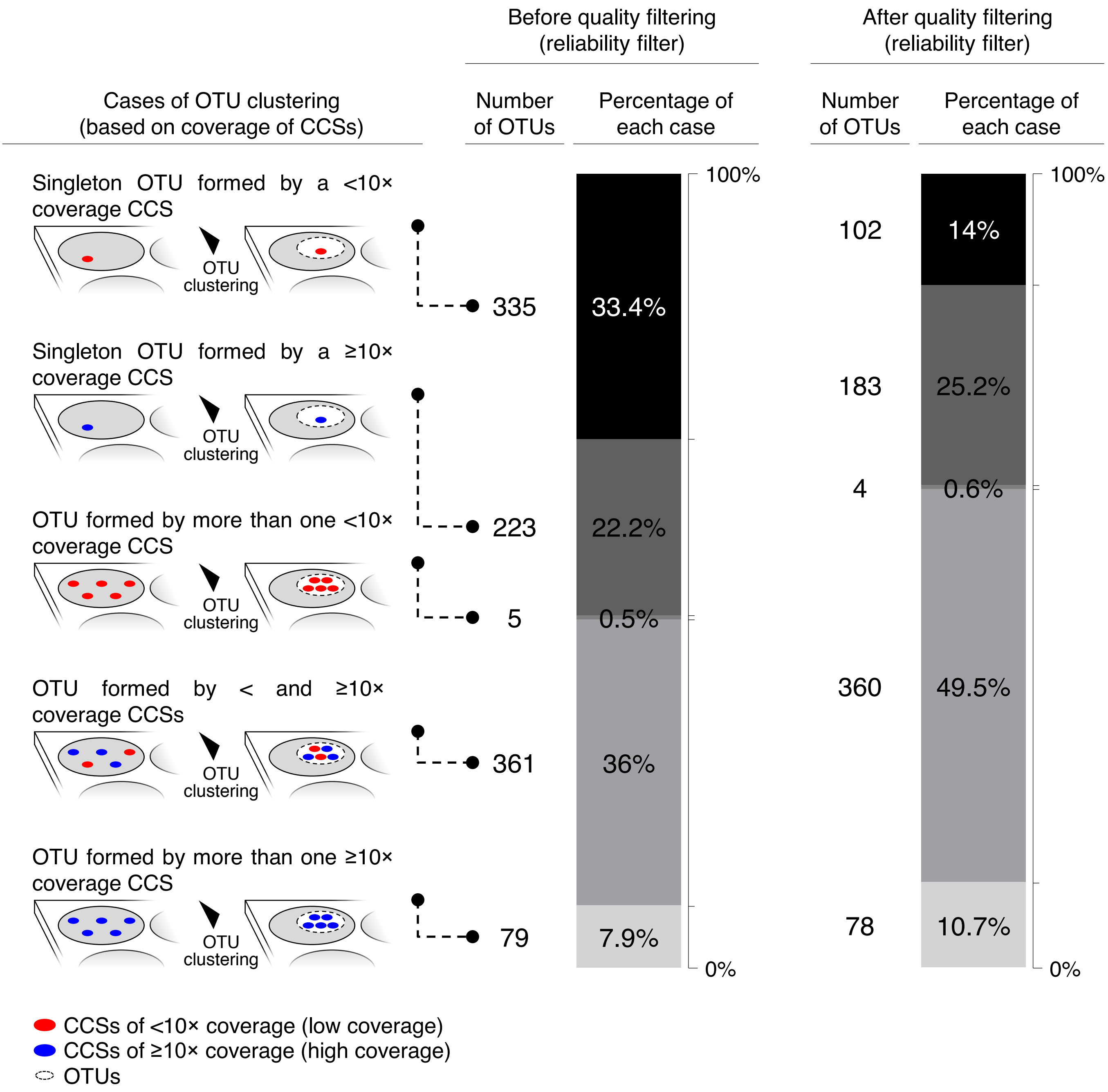

Supplementary Figure 5. Counts of OTUs based on CCS coverage. An OTU has a higher probability of representing a true biological sequence depending on the CCSs involved in the clustering event. OTUs originated from clustering of high-coverage CCSs have a higher chance of representing true biological sequences. All possible combinations of low- and high-coverage CCSs in OTU clustering were counted in order to estimate the overall reliability of OTUs. Before applying the quality filter, a large number of OTUs originated from singletons, low-coverage CCSs. Those OTUs have a higher probability of arising from errors and were removed by the quality filter (reliability filter).

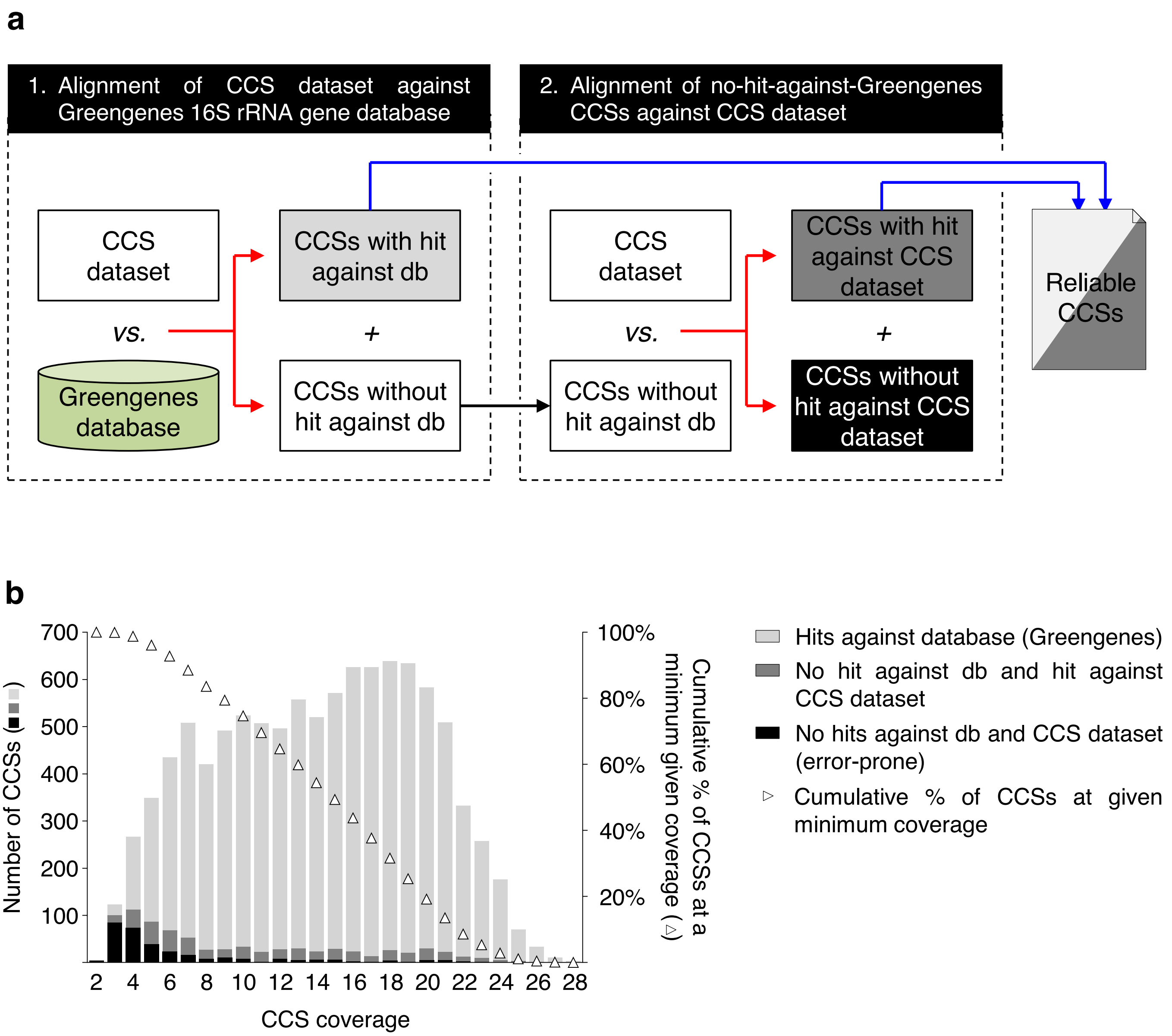

Supplementary Figure 6. Pipeline of reliability filter and advantages of reliability filter over coverage filter. (**a**) (1) Alignment of the CCS dataset against a curated 16S rRNA gene database; CCSs with at least one hit against the database were considered reliable and were saved. (2) CCSs without a hit against the database were re-aligned to the CCS dataset. CCSs with a hit against the CCS dataset were considered reliable and were saved. CCSs without at least one hit were considered error-prone and discarded. (**b**) The graph shows the number of CCSs that have a hit against the Greengenes 16S rRNA gene database (light gray, left axis), the number of CCSs without any hit against the Greengenes database but at least one hit against any other CCS on the dataset (strong gray, left axis), and the number of CCSs without any hit against both the Greengenes and CCS dataset (error-prone CCSs, black, left axis) per CCS coverage. Triangles show the cumulative percentage of CCSs at a given coverage threshold. If one chose to filter CCSs based on coverage, a significant fraction of reliable CCS would be lost. db: database.

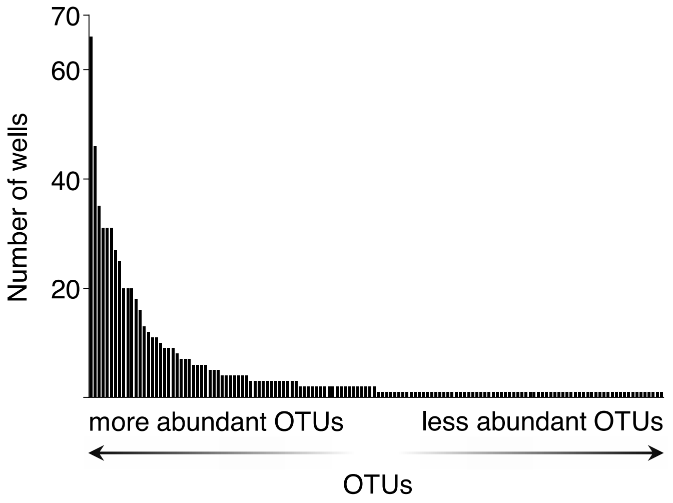

Supplementary Figure 7. Redundancy of OTUs found in the five plates of the sugarcane community-based culture collection (CBC). Distribution of OTUs based on their presence in different wells.

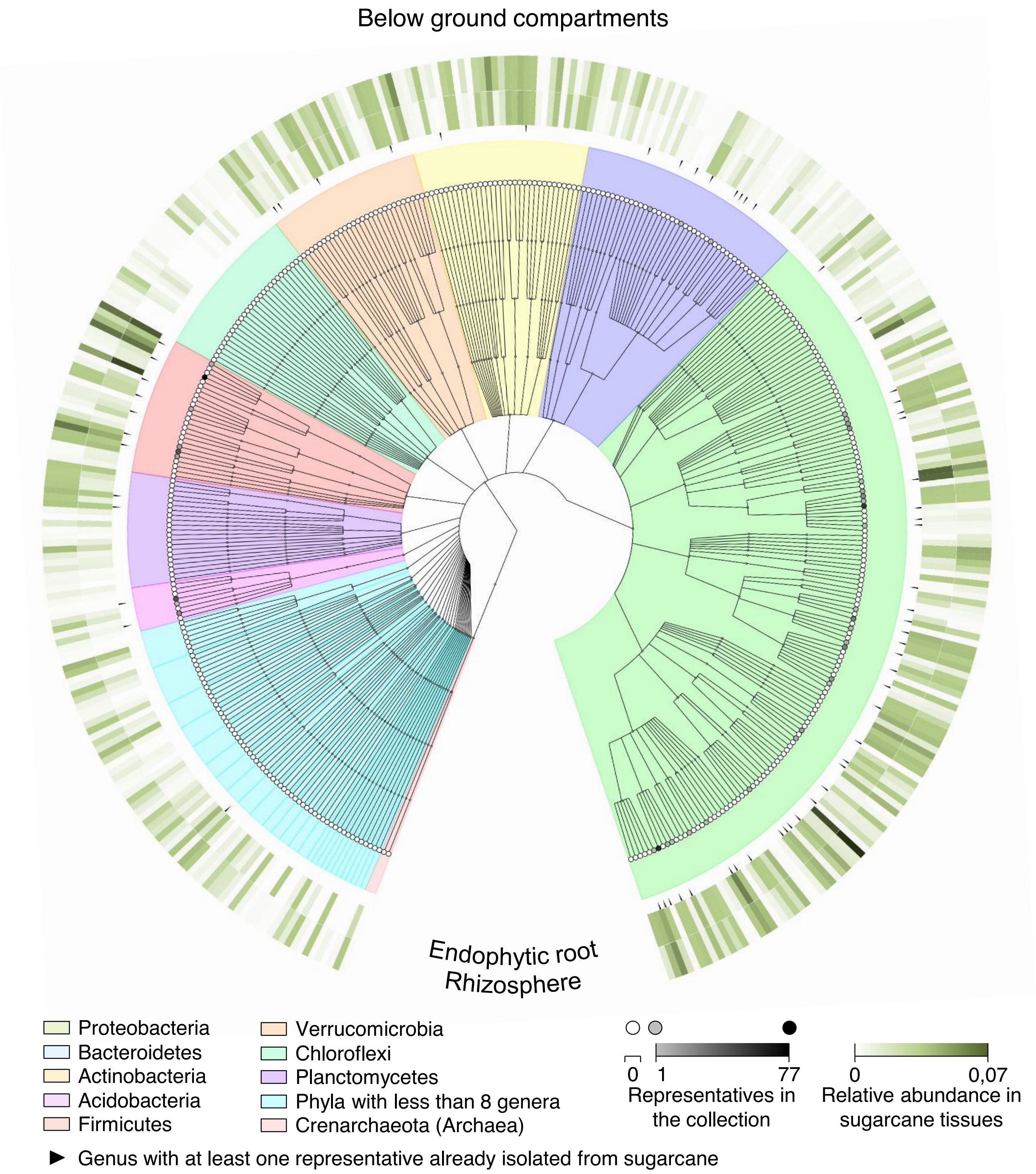

Supplementary Figure 8. Full-version of cladogram for cross-referencing of OTUs in the five plates of the sugarcane community-based culture collection of the root core OTUs from sugarcane microbiome profile presented in Figure 3a.

Multiplex amplicon sequencing for microbe identification in community-based culture collections

Jaderson Silveira Leite Armanhi[1,6], Rafael Soares Correa de Souza[1,6], Laura Migliorini de Araújo[1], Vagner Katsumi Okura[1], Piotr Mieczkowski[2], Juan Imperial[3,4] and Paulo Arruda[1,5*]

[1]Centro de Biologia Molecular e Engenharia Genética, Universidade Estadual de Campinas (UNICAMP), 13083-875, Campinas, SP, Brazil.

[2]Department of Genetics, University of North Carolina, Chapel Hill, North Carolina, USA.

[3]Centro de Biotecnología y Genómica de Plantas, Universidad Politécnica de Madrid (UPM) – Instituto Nacional de Investigación y Tecnología Agraria y Alimentaria (INIA), Campus Montegancedo UPM 28223 – Pozuelo de Alarcón (Madrid), Spain.

[4]Consejo Superior de Investigaciones Científicas, Madrid, Spain.

[5]Departamento de Genética e Evolução, Instituto de Biologia, Universidade Estadual de Campinas (UNICAMP), 13083-970, Campinas, SP, Brazil.

[6]These authors contributed equally to this work.

*Correspondence should be addressed to P.A. (parruda@unicamp.br).

Running title: Community-based culture collections

SUPPLEMENTARY TABLES

Supplementary Table 1. Taxonomical classification of OTUs from community-based culture collection (CBC) of sugarcane. OTUs were classified using two methods: (1) a trained RDP[1] database (www.drive5.com/utax/rdp_16s.fa) and the UTAX algorithm (www.drive5.com/usearch/manual/utax_algo.html; confidence value shown for all the taxonomic levels, between parentheses); (2) against the Greengenes[2] database using the RDP classifier[3] (confidence score shown in the right column).

Well and OTU	UTAX against RDP database (confidence value between parentheses)	RDP classifier against Greengenes database	
		Taxonomy assigned	Confidence score
bc01_A01;OTUwell_1	Bacteria(100.0),"Bacteroidetes"(100.0),"Sphingobacteria"(98.6),"Sphingobacteriales"(98.6),Sphingobacteriaceae(98.6),Pedobacter(98.6)	k__Bacteria;p__Bacteroidetes;c__Sphingobacteriia;o__Sphingobacteriales;f__Sphingobacteriaceae;g__Pedobacter;s__	1,00
bc01_A01;OTUwell_2	Bacteria(100.0),"Bacteroidetes"(100.0),Flavobacteria(98.0),"Flavobacteriales"(98.0),Flavobacteriaceae(98.0),Flavobacterium(93.8)	k__Bacteria;p__Bacteroidetes;c__Flavobacteriia;o__Flavobacteriales;f__Flavobacteriaceae;g__Flavobacterium;s__	1,00
bc01_A01;OTUwell_3	Bacteria(100.0),"Bacteroidetes"(100.0),"Sphingobacteria"(99.8),"Sphingobacteriales"(99.6),Chitinophagaceae(99.6),Chitinophaga(99.6)	k__Bacteria;p__Bacteroidetes;c__[Saprospirae];o__[Saprospirales];f__Chitinophagaceae;g__Chitinophaga;s__	1,00
bc01_A02;OTUwell_1	Bacteria(100.0),"Proteobacteria"(100.0),Gammaproteobacteria(99.8),Xanthomonadales(99.2),Xanthomonadaceae(98.9),Lysobacter(98.9)	k__Bacteria;p__Proteobacteria;c__Gammaproteobacteria;o__Xanthomonadales;f__Xanthomonadaceae;g__Lysobacter;s__	1,00
bc01_A02;OTUwell_2	Bacteria(100.0),"Proteobacteria"(100.0),Alphaproteobacteria(98.7),Rhizobiales(98.7),Rhizobiaceae(98.7),Rhizobium(98.7)	k__Bacteria;p__Proteobacteria;c__Alphaproteobacteria;o__Rhizobiales;f__Rhizobiaceae;g__Rhizobium;s__	1,00
bc01_A02;OTUwell_3	Bacteria(100.0),"Proteobacteria"(100.0),Gammaproteobacteria(99.5),Pseudomonadales(99.3),Pseudomonadaceae(99.1),Pseudomonas(96.3)	k__Bacteria;p__Proteobacteria;c__Gammaproteobacteria;o__Pseudomonadales;f__Pseudomonadaceae	1,00
bc01_A03;OTUwell_1	Bacteria(100.0),"Proteobacteria"(100.0),Gammaproteobacteria(99.7),Xanthomonadales(99.1),Xanthomonadaceae(98.8),Lysobacter(98.8)	k__Bacteria;p__Proteobacteria;c__Gammaproteobacteria;o__Xanthomonadales;f__Xanthomonadaceae;g__Lysobacter;s__	0,99
bc01_A04;OTUwell_1	Bacteria(100.0),"Bacteroidetes"(100.0),Flavobacteria(98.2),"Flavobacteriales"(98.2),Flavobacteriaceae(98.2),Flavobacterium(95.4)	k__Bacteria;p__Bacteroidetes;c__Flavobacteriia;o__Flavobacteriales;f__Flavobacteriaceae;g__Flavobacterium;s__	1,00
bc01_A05;OTUwell_1	Bacteria(100.0),"Proteobacteria"(100.0),Gammaproteobacteria(99.8),Xanthomonadales(99.2),Xanthomonadaceae(98.9),Lysobacter(98.9)	k__Bacteria;p__Proteobacteria;c__Gammaproteobacteria;o__Xanthomonadales;f__Xanthomonadaceae;g__Lysobacter;s__	1,00
bc01_A05;OTUwell_2	Bacteria(100.0),"Bacteroidetes"(100.0),"Sphingobacteria"(98.2),"Sphingobacteriales"(98.2),Sphingobacteriaceae(98.2),Mucilaginibacter(96.3)	k__Bacteria;p__Bacteroidetes;c__Sphingobacteriia;o__Sphingobacteriales;f__Sphingobacteriaceae;g__;s__	1,00
bc01_A06;OTUwell_1	Bacteria(100.0),"Proteobacteria"(100.0),Alphaproteobacteria(99.6),Caulobacterales(99.6),Caulobacteraceae(99.6),Caulobacter(99.2)	k__Bacteria;p__Proteobacteria;c__Alphaproteobacteria;o__Caulobacterales;f__Caulobacteraceae;g__Caulobacter;s__	1,00
bc01_A08;OTUwell_1	Bacteria(100.0),"Proteobacteria"(100.0),Betaproteobacteria(99.6),Burkholderiales(99.5),Burkholderiaceae(99.5),Burkholderia(99.5)	k__Bacteria;p__Proteobacteria;c__Betaproteobacteria;o__Burkholderiales;f__Burkholderiaceae;g__Burkholderia;s__	1,00
bc01_A08;OTUwell_2	Bacteria(100.0),"Proteobacteria"(100.0),Alphaproteobacteria(98.7),Rhizobiales(98.7),Rhizobiaceae(98.7),Rhizobium(98.7)	k__Bacteria;p__Proteobacteria;c__Alphaproteobacteria;o__Rhizobiales;f__Rhizobiaceae;g__Rhizobium;s__	1,00
bc01_A09;OTUwell_1	Bacteria(100.0),"Proteobacteria"(100.0),Alphaproteobacteria(98.5),Sphingomonadales(97.9),Sphingomonadaceae(96.4),Novosphingobium(82.2)	k__Bacteria;p__Proteobacteria;c__Alphaproteobacteria;o__Sphingomonadales;f__Sphingomonadaceae;g__Novosphingobium;s__	0,99
bc01_A09;OTUwell_2	Bacteria(100.0),"Proteobacteria"(100.0),Betaproteobacteria(99.6),Burkholderiales(99.5),Burkholderiaceae(99.5),Burkholderia(99.5)	k__Bacteria;p__Proteobacteria;c__Betaproteobacteria;o__Burkholderiales;f__Burkholderiaceae;g__Burkholderia;s__	1,00
bc01_A10;OTUwell_1	Bacteria(100.0),"Proteobacteria"(100.0),Betaproteobacteria(99.2),Burkholderiales(99.2),Burkholderiaceae(99.0),Burkholderia(98.7)	k__Bacteria;p__Proteobacteria;c__Betaproteobacteria;o__Burkholderiales;f__Burkholderiaceae;g__Burkholderia;s__	0,90
bc01_A11;OTUwell_1	Bacteria(100.0),"Proteobacteria"(100.0),Gammaproteobacteria(99.6),Xanthomonadales(99.4),Xanthomonadaceae(99.4),Dyella(92.4)	k__Bacteria;p__Proteobacteria;c__Gammaproteobacteria;o__Xanthomonadales;f__Xanthomonadaceae;g__Dyella;s__	0,98
bc01_B01;OTUwell_1	Bacteria(100.0),"Bacteroidetes"(100.0),"Sphingobacteria"(98.6),"Sphingobacteriales"(98.6),Sphingobacteriaceae(98.6),Pedobacter(98.6)	k__Bacteria;p__Bacteroidetes;c__Sphingobacteriia;o__Sphingobacteriales;f__Sphingobacteriaceae;g__Pedobacter;s__	1,00
bc01_B01;OTUwell_2	Bacteria(100.0),"Proteobacteria"(100.0),Betaproteobacteria(98.9),Burkholderiales(98.9),Burkholderiaceae(98.6),Burkholderia(97.1)	k__Bacteria;p__Proteobacteria;c__Betaproteobacteria;o__Burkholderiales;f__Burkholderiaceae;g__Burkholderia	0,82
bc01_B02;OTUwell_1	Bacteria(100.0),"Actinobacteria"(100.0),Actinobacteria(100.0),Actinomycetales(99.6),Microbacteriaceae(99.5),Microbacterium(99.0)	k__Bacteria;p__Actinobacteria;c__Actinobacteria;o__Actinomycetales;f__Microbacteriaceae;g__Microbacterium;s__	0,96
bc01_B05;OTUwell_1	Bacteria(100.0),"Bacteroidetes"(100.0),"Sphingobacteria"(99.6),"Sphingobacteriales"(99.6),Chitinophagaceae(99.6),Chitinophaga(99.2)	k__Bacteria;p__Bacteroidetes;c__[Saprospirae];o__[Saprospirales];f__Chitinophagaceae;g__Chitinophaga;s__	1,00
bc01_B05;OTUwell_2	Bacteria(100.0),"Bacteroidetes"(100.0),Flavobacteria(96.2),"Flavobacteriales"(96.2),Flavobacteriaceae(95.7),Flavobacterium(84.7)	k__Bacteria;p__Bacteroidetes;c__Flavobacteriia;o__Flavobacteriales;f__Flavobacteriaceae;g__Flavobacterium;s__	0,98
bc01_B06;OTUwell_1	Bacteria(100.0),"Proteobacteria"(100.0),Gammaproteobacteria(100.0),"Enterobacteriales"(99.1),Enterobacteriaceae(98.7),Cedecea(94.5)	k__Bacteria;p__Proteobacteria;c__Gammaproteobacteria;o__Enterobacteriales;f__Enterobacteriaceae	1,00
bc01_B07;OTUwell_1	Bacteria(100.0),Firmicutes(100.0),Bacilli(99.7),Bacillales(99.5),Bacillaceae_1(99.4),Bacillus(99.4)	k__Bacteria;p__Firmicutes;c__Bacilli;o__Bacillales;f__Bacillaceae;g__;s__	0,98
bc01_B07;OTUwell_2	Bacteria(100.0),"Proteobacteria"(100.0),Gammaproteobacteria(99.7),Xanthomonadales(99.5),Xanthomonadaceae(99.5),Dyella(97.5)	k__Bacteria;p__Proteobacteria;c__Gammaproteobacteria;o__Xanthomonadales;f__Xanthomonadaceae;g__Dyella;s__	1,00
bc01_B07;OTUwell_3	Bacteria(100.0),"Proteobacteria"(100.0),Gammaproteobacteria(99.3),Xanthomonadales(99.2),Xanthomonadaceae(99.0),Dyella(90.8)	k__Bacteria;p__Proteobacteria;c__Gammaproteobacteria;o__Xanthomonadales;f__Xanthomonadaceae;g__Dyella;s__	0,99
bc01_B07;OTUwell_4	Bacteria(100.0),"Bacteroidetes"(100.0),"Sphingobacteria"(98.2),"Sphingobacteriales"(98.2),Sphingobacteriaceae(98.2),Mucilaginibacter(95.9)	k__Bacteria;p__Bacteroidetes;c__Sphingobacteriia;o__Sphingobacteriales;f__Sphingobacteriaceae;g__;s__	1,00
bc01_B09;OTUwell_1	Bacteria(100.0),"Proteobacteria"(100.0),Alphaproteobacteria(98.4),Rhizobiales(98.4),Rhizobiaceae(98.3),Rhizobium(98.3)	k__Bacteria;p__Proteobacteria;c__Alphaproteobacteria;o__Rhizobiales;f__Rhizobiaceae;g__Rhizobium;s__	0,97
bc01_B09;OTUwell_2	Bacteria(100.0),"Proteobacteria"(100.0),Gammaproteobacteria(99.7),Xanthomonadales(99.5),Xanthomonadaceae(99.5),Dyella(98.0)	k__Bacteria;p__Proteobacteria;c__Gammaproteobacteria;o__Xanthomonadales;f__Xanthomonadaceae;g__Dyella;s__	0,99
bc01_B11;OTUwell_1	Bacteria(100.0),"Proteobacteria"(100.0),Betaproteobacteria(99.5),Burkholderiales(99.5),Burkholderiaceae(99.5),Burkholderia(99.5)	k__Bacteria;p__Proteobacteria;c__Betaproteobacteria;o__Burkholderiales;f__Burkholderiaceae;g__Burkholderia;s__	1,00
bc01_B12;OTUwell_1	Bacteria(100.0),"Proteobacteria"(100.0),Gammaproteobacteria(99.8),Pseudomonadales(99.6),Pseudomonadaceae(99.6),Pseudomonas(99.2)	k__Bacteria;p__Proteobacteria;c__Gammaproteobacteria;o__Pseudomonadales;f__Pseudomonadaceae;g__Pseudomonas;s__nitroreducens	0,84
bc01_B12;OTUwell_2	Bacteria(100.0),"Proteobacteria"(100.0),Betaproteobacteria(99.5),Burkholderiales(99.5),Burkholderiaceae(99.5),Burkholderia(99.4)	k__Bacteria;p__Proteobacteria;c__Betaproteobacteria;o__Burkholderiales;f__Burkholderiaceae;g__Burkholderia;s__	1,00
bc01_B12;OTUwell_3	Bacteria(100.0),"Proteobacteria"(100.0),Alphaproteobacteria(97.9),Rhizobiales(97.9),Rhizobiaceae(97.9),Ensifer(94.5)	k__Bacteria;p__Proteobacteria;c__Alphaproteobacteria;o__Rhizobiales;f__Rhizobiaceae	1,00
bc01_B12;OTUwell_4	Bacteria(100.0),Firmicutes(100.0),Bacilli(99.6),Bacillales(99.5),Bacillaceae_1(99.1),Bacillus(99.1)	k__Bacteria;p__Firmicutes;c__Bacilli;o__Bacillales;f__Bacillaceae;g__Bacillus	1,00
bc01_C01;OTUwell_1	Bacteria(100.0),Firmicutes(100.0),Bacilli(99.7),Bacillales(99.5),Bacillaceae_1(99.2),Bacillus(99.2)	k__Bacteria;p__Firmicutes;c__Bacilli;o__Bacillales;f__Bacillaceae;g__Bacillus	1,00
bc01_C01;OTUwell_2	Bacteria(100.0),"Bacteroidetes"(100.0),"Sphingobacteria"(99.9),"Sphingobacteriales"(99.9),Chitinophagaceae(99.9),Chitinophaga(99.9)	k__Bacteria;p__Bacteroidetes;c__[Saprospirae];o__[Saprospirales];f__Chitinophagaceae;g__Chitinophaga;s__	1,00
bc01_C01;OTUwell_3	Bacteria(100.0),Firmicutes(100.0),Bacilli(99.6),Bacillales(99.5),Bacillaceae_1(99.4),Bacillus(99.4)	k__Bacteria;p__Firmicutes;c__Bacilli;o__Bacillales;f__Bacillaceae;g__;s__	1,00
bc01_C01;OTUwell_4	Bacteria(100.0),"Bacteroidetes"(100.0),"Sphingobacteria"(99.3),"Sphingobacteriales"(99.3),Chitinophagaceae(99.3),Chitinophaga(97.5)	k__Bacteria;p__Bacteroidetes;c__[Saprospirae];o__[Saprospirales];f__Chitinophagaceae;g__Chitinophaga;s__	1,00
bc01_C01;OTUwell_5	Bacteria(100.0),"Bacteroidetes"(100.0),Flavobacteria(97.6),"Flavobacteriales"(97.6),Flavobacteriaceae(97.6),Flavobacterium(92.4)	k__Bacteria;p__Bacteroidetes;c__Flavobacteriia;o__Flavobacteriales;f__Flavobacteriaceae;g__Flavobacterium;s__	0,94
bc01_C01;OTUwell_6	Bacteria(100.0),"Proteobacteria"(100.0),Betaproteobacteria(98.6),Burkholderiales(98.6),Burkholderiaceae(98.6),Wautersia(86.9)	k__Bacteria;p__Proteobacteria;c__Betaproteobacteria;o__Burkholderiales;f__Oxalobacteraceae;g__Cupriavidus;s__	1,00
bc01_C01;OTUwell_7	Bacteria(100.0),"Proteobacteria"(100.0),Betaproteobacteria(99.3),Burkholderiales(99.3),Burkholderiaceae(99.3),Wautersia(94.5)	k__Bacteria;p__Proteobacteria;c__Betaproteobacteria;o__Burkholderiales;f__Oxalobacteraceae;g__Cupriavidus;s__	1,00
bc01_C01;OTUwell_8	Bacteria(100.0),"Proteobacteria"(100.0),Alphaproteobacteria(98.6),Rhizobiales(98.6),Rhizobiaceae(98.6),Rhizobium(98.6)	k__Bacteria;p__Proteobacteria;c__Alphaproteobacteria;o__Rhizobiales;f__Rhizobiaceae;g__Rhizobium;s__	0,99
bc01_C01;OTUwell_9	Bacteria(100.0),"Proteobacteria"(100.0),Betaproteobacteria(98.6),Burkholderiales(98.6),Burkholderiaceae(98.6),Wautersia(85.8)	k__Bacteria;p__Proteobacteria;c__Betaproteobacteria;o__Burkholderiales;f__Oxalobacteraceae;g__Cupriavidus;s__	1,00
bc01_C01;OTUwell_10	Bacteria(100.0),Firmicutes(100.0),Bacilli(99.2),Bacillales(99.1),Bacillaceae_1(98.2),Bacillus(97.7)	k__Bacteria;p__Firmicutes;c__Bacilli;o__Bacillales;f__Bacillaceae;g__Bacillus	1,00
bc01_C01;OTUwell_11	Bacteria(100.0),"Bacteroidetes"(100.0),"Sphingobacteria"(99.5),"Sphingobacteriales"(99.5),Chitinophagaceae(99.5),Chitinophaga(98.7)	k__Bacteria;p__Bacteroidetes;c__[Saprospirae];o__[Saprospirales];f__Chitinophagaceae;g__Chitinophaga;s__	1,00
bc01_C02;OTUwell_1	Bacteria(100.0),"Proteobacteria"(100.0),Gammaproteobacteria(99.6),Xanthomonadales(98.5),Xanthomonadaceae(97.5),Pseudoxanthomonas(94.5)	k__Bacteria;p__Proteobacteria;c__Gammaproteobacteria;o__Xanthomonadales;f__Xanthomonadaceae	1,00
bc01_C02;OTUwell_2	Bacteria(100.0),"Proteobacteria"(100.0),Gammaproteobacteria(99.1),Xanthomonadales(95.6),Xanthomonadaceae(94.6),Pseudoxanthomonas(82.2)	k__Bacteria;p__Proteobacteria;c__Gammaproteobacteria;o__Xanthomonadales;f__Xanthomonadaceae;g__Pseudoxanthomonas;s__	0,83
bc01_C02;OTUwell_3	Bacteria(100.0),Firmicutes(100.0),Bacilli(99.2),Bacillales(99.2),Bacillaceae_1(98.7),Bacillus(98.7)	k__Bacteria;p__Firmicutes;c__Bacilli;o__Bacillales;f__Bacillaceae;g__;s__	0,87
bc01_C02;OTUwell_4	Bacteria(100.0),"Proteobacteria"(100.0),Alphaproteobacteria(98.0),Rhizobiales(98.0),Rhizobiaceae(97.8),Rhizobium(96.6)	k__Bacteria;p__Proteobacteria;c__Alphaproteobacteria;o__Rhizobiales;f__Rhizobiaceae;g__Rhizobium;s__	0,97
bc01_C03;OTUwell_1	Bacteria(100.0),"Bacteroidetes"(100.0),"Sphingobacteria"(98.4),"Sphingobacteriales"(98.4),Sphingobacteriaceae(98.4),Pedobacter(98.0)	k__Bacteria;p__Bacteroidetes;c__Sphingobacteriia;o__Sphingobacteriales;f__Sphingobacteriaceae;g__Pedobacter;s__	1,00
bc01_C03;OTUwell_2	Bacteria(100.0),"Proteobacteria"(100.0),Gammaproteobacteria(99.7),Xanthomonadales(99.5),Xanthomonadaceae(99.5),Dyella(97.5)	k__Bacteria;p__Proteobacteria;c__Gammaproteobacteria;o__Xanthomonadales;f__Xanthomonadaceae;g__Dyella;s__	1,00
bc01_C03;OTUwell_3	Bacteria(100.0),Firmicutes(100.0),Bacilli(99.7),Bacillales(99.5),Bacillaceae_1(99.2),Bacillus(99.2)	k__Bacteria;p__Firmicutes;c__Bacilli;o__Bacillales;f__Bacillaceae;g__Bacillus	1,00
bc01_C04;OTUwell_1	Bacteria(100.0),"Bacteroidetes"(100.0),"Sphingobacteria"(98.4),"Sphingobacteriales"(98.4),Sphingobacteriaceae(98.4),Pedobacter(97.7)	k__Bacteria;p__Bacteroidetes;c__Sphingobacteriia;o__Sphingobacteriales;f__Sphingobacteriaceae;g__Pedobacter;s__	1,00
bc01_C04;OTUwell_2	Bacteria(100.0),"Bacteroidetes"(100.0),"Sphingobacteria"(97.2),"Sphingobacteriales"(97.2),Sphingobacteriaceae(97.2),Pedobacter(91.7)	k__Bacteria;p__Bacteroidetes;c__Sphingobacteriia;o__Sphingobacteriales;f__Sphingobacteriaceae;g__Pedobacter;s__	1,00
bc01_C05;OTUwell_1	Bacteria(100.0),"Actinobacteria"(100.0),Actinobacteria(100.0),Actinomycetales(100.0),Microbacteriaceae(99.6),Microbacterium(99.3)	k__Bacteria;p__Actinobacteria;c__Actinobacteria;o__Actinomycetales;f__Microbacteriaceae;g__Microbacterium	1,00
bc01_C05;OTUwell_2	Bacteria(100.0),"Bacteroidetes"(100.0),"Sphingobacteria"(100.0),"Sphingobacteriales"(100.0),Chitinophagaceae(100.0),Chitinophaga(99.7)	k__Bacteria;p__Bacteroidetes;c__[Saprospirae];o__[Saprospirales];f__Chitinophagaceae;g__Chitinophaga;s__	1,00
bc01_C05;OTUwell_3	Bacteria(100.0),"Proteobacteria"(100.0),Alphaproteobacteria(99.4),Rhizobiales(99.4),Bradyrhizobiaceae(99.2),Bosea(99.2)	k__Bacteria;p__Proteobacteria;c__Alphaproteobacteria;o__Rhizobiales;f__Beijerinckiaceae;g__;s__	0,81
bc01_C06;OTUwell_1	Bacteria(100.0),"Proteobacteria"(100.0),Gammaproteobacteria(99.7),Xanthomonadales(99.5),Xanthomonadaceae(99.5),Dyella(97.5)	k__Bacteria;p__Proteobacteria;c__Gammaproteobacteria;o__Xanthomonadales;f__Xanthomonadaceae;g__Dyella;s__	0,99
bc01_C07;OTUwell_1	Bacteria(100.0),"Bacteroidetes"(100.0),"Sphingobacteria"(99.6),"Sphingobacteriales"(99.6),Chitinophagaceae(99.6),Chitinophaga(99.1)	k__Bacteria;p__Bacteroidetes;c__[Saprospirae];o__[Saprospirales];f__Chitinophagaceae;g__Chitinophaga;s__	1,00
bc01_C07;OTUwell_2	Bacteria(100.0),"Bacteroidetes"(100.0),"Sphingobacteria"(99.2),"Sphingobacteriales"(99.2),Chitinophagaceae(99.2),Chitinophaga(95.4)	k__Bacteria;p__Bacteroidetes;c__[Saprospirae];o__[Saprospirales];f__Chitinophagaceae;g__Chitinophaga;s__	1,00
bc01_C08;OTUwell_1	Bacteria(100.0),"Proteobacteria"(100.0),Alphaproteobacteria(99.2),Rhodospirillales(99.2),Rhodospirillaceae(99.1),Inquilinus(99.1)	k__Bacteria;p__Proteobacteria;c__Alphaproteobacteria;o__Rhodospirillales;f__Rhodospirillaceae;g__Inquilinus;s__limosus	1,00
bc01_C09;OTUwell_1	Bacteria(100.0),"Actinobacteria"(100.0),Actinobacteria(100.0),Actinomycetales(99.7),Micrococcaceae(99.2),Arthrobacter(99.0)	k__Bacteria;p__Actinobacteria;c__Actinobacteria;o__Actinomycetales;f__Micrococcaceae;g__;s__	1,00

bc01_C10;OTUwell_1	Bacteria(100.0),Firmicutes(100.0),Bacilli(99.7),Bacillales(99.5),Bacillaceae_1(99.5),Bacillus(99.5)	k__Bacteria;p__Firmicutes;c__Bacilli;o__Bacillales;f__Bacillaceae;g__;s__	1,00
bc01_C11;OTUwell_1	Bacteria(100.0),"Proteobacteria"(100.0),Alphaproteobacteria(99.7),Caulobacterales(99.6),Caulobacteraceae(99.6),Asticcacaulis(99.6)	k__Bacteria;p__Proteobacteria;c__Alphaproteobacteria;o__Caulobacterales;f__Caulobacteraceae;g__Asticcacaulis;s__biprosthecium	1,00
bc01_C12;OTUwell_1	Bacteria(100.0),"Bacteroidetes"(100.0),"Sphingobacteria"(98.6),"Sphingobacteriales"(98.6),Sphingobacteriaceae(98.6),Pedobacter(98.6)	k__Bacteria;p__Bacteroidetes;c__Sphingobacteriia;o__Sphingobacteriales;f__Sphingobacteriaceae;g__Pedobacter;s__	1,00
bc01_C12;OTUwell_2	Bacteria(100.0),"Proteobacteria"(100.0),Gammaproteobacteria(99.8),Xanthomonadales(99.6),Xanthomonadaceae(99.6),Dyella(98.3)	k__Bacteria;p__Proteobacteria;c__Gammaproteobacteria;o__Xanthomonadales;f__Xanthomonadaceae;g__Dyella;s__	0,97
bc01_C12;OTUwell_3	Bacteria(100.0),Firmicutes(100.0),Bacilli(99.6),Bacillales(99.5),Bacillaceae_1(99.2),Bacillus(99.2)	k__Bacteria;p__Firmicutes;c__Bacilli;o__Bacillales;f__Bacillaceae;g__Bacillus	1,00
bc01_D01;OTUwell_1	Bacteria(100.0),"Bacteroidetes"(100.0),"Sphingobacteria"(100.0),"Sphingobacteriales"(100.0),Chitinophagaceae(100.0),Chitinophaga(99.9)	k__Bacteria;p__Bacteroidetes;c__[Saprospirae];o__[Saprospirales];f__Chitinophagaceae;g__Chitinophaga;s__	1,00
bc01_D01;OTUwell_2	Bacteria(100.0),Firmicutes(100.0),Bacilli(99.7),Bacillales(99.5),Bacillaceae_1(99.2),Bacillus(99.2)	k__Bacteria;p__Firmicutes;c__Bacilli;o__Bacillales;f__Bacillaceae;g__Bacillus	1,00
bc01_D01;OTUwell_3	Bacteria(100.0),"Bacteroidetes"(100.0),"Sphingobacteria"(99.6),"Sphingobacteriales"(99.6),Chitinophagaceae(99.6),Chitinophaga(99.2)	k__Bacteria;p__Bacteroidetes;c__[Saprospirae];o__[Saprospirales];f__Chitinophagaceae;g__Chitinophaga;s__	1,00
bc01_D02;OTUwell_1	Bacteria(100.0),"Bacteroidetes"(100.0),"Sphingobacteria"(99.7),"Sphingobacteriales"(99.6),Chitinophagaceae(99.6),Chitinophaga(99.6)	k__Bacteria;p__Bacteroidetes;c__[Saprospirae];o__[Saprospirales];f__Chitinophagaceae;g__Chitinophaga;s__	1,00
bc01_D02;OTUwell_2	Bacteria(100.0),"Bacteroidetes"(100.0),"Sphingobacteria"(99.4),"Sphingobacteriales"(99.4),Chitinophagaceae(99.4),Chitinophaga(98.7)	k__Bacteria;p__Bacteroidetes;c__[Saprospirae];o__[Saprospirales];f__Chitinophagaceae;g__Chitinophaga;s__	1,00
bc01_D02;OTUwell_3	Bacteria(100.0),Firmicutes(100.0),Bacilli(99.6),Bacillales(99.5),Bacillaceae_1(99.1),Bacillus(99.1)	k__Bacteria;p__Firmicutes;c__Bacilli;o__Bacillales;f__Bacillaceae;g__Bacillus	1,00
bc01_D02;OTUwell_4	Bacteria(100.0),"Bacteroidetes"(100.0),"Sphingobacteria"(99.3),"Sphingobacteriales"(99.3),Chitinophagaceae(99.3),Chitinophaga(98.0)	k__Bacteria;p__Bacteroidetes;c__[Saprospirae];o__[Saprospirales];f__Chitinophagaceae;g__Chitinophaga;s__	0,99
bc01_D03;OTUwell_1	Bacteria(100.0),"Bacteroidetes"(100.0),"Sphingobacteria"(99.6),"Sphingobacteriales"(99.6),Chitinophagaceae(99.6),Chitinophaga(99.2)	k__Bacteria;p__Bacteroidetes;c__[Saprospirae];o__[Saprospirales];f__Chitinophagaceae;g__Chitinophaga;s__	1,00
bc01_D03;OTUwell_2	Bacteria(100.0),"Proteobacteria"(100.0),Gammaproteobacteria(99.6),Xanthomonadales(99.3),Xanthomonadaceae(99.3),Dyella(93.2)	k__Bacteria;p__Proteobacteria;c__Gammaproteobacteria;o__Xanthomonadales;f__Xanthomonadaceae;g__Dyella;s__	0,98
bc01_D03;OTUwell_3	Bacteria(100.0),"Bacteroidetes"(100.0),"Sphingobacteria"(99.0),"Sphingobacteriales"(99.0),Chitinophagaceae(99.0),Chitinophaga(94.5)	k__Bacteria;p__Bacteroidetes;c__[Saprospirae];o__[Saprospirales];f__Chitinophagaceae;g__Chitinophaga;s__	1,00
bc01_D03;OTUwell_4	Bacteria(100.0),"Bacteroidetes"(100.0),"Sphingobacteria"(99.1),"Sphingobacteriales"(99.1),Chitinophagaceae(99.1),Chitinophaga(93.8)	k__Bacteria;p__Bacteroidetes;c__[Saprospirae];o__[Saprospirales];f__Chitinophagaceae;g__Chitinophaga;s__	1,00
bc01_D04;OTUwell_1	Bacteria(100.0),"Bacteroidetes"(100.0),"Sphingobacteria"(98.9),"Sphingobacteriales"(98.9),Chitinophagaceae(98.9),Niastella(79.9)	k__Bacteria;p__Bacteroidetes;c__[Saprospirae];o__[Saprospirales];f__Chitinophagaceae;g__;s__	1,00
bc01_D05;OTUwell_1	Bacteria(100.0),"Actinobacteria"(100.0),Actinobacteria(100.0),Actinomycetales(100.0),Microbacteriaceae(99.6),Microbacterium(99.3)	k__Bacteria;p__Actinobacteria;c__Actinobacteria;o__Actinomycetales;f__Microbacteriaceae;g__Microbacterium	1,00
bc01_D06;OTUwell_1	Bacteria(100.0),"Proteobacteria"(100.0),Gammaproteobacteria(99.6),Pseudomonadales(99.4),Pseudomonadaceae(99.4),Pseudomonas(98.8)	k__Bacteria;p__Proteobacteria;c__Gammaproteobacteria;o__Pseudomonadales;f__Pseudomonadaceae;g__Pseudomonas;s__	0,89
bc01_D07;OTUwell_1	Bacteria(100.0),"Proteobacteria"(100.0),Gammaproteobacteria(99.6),Xanthomonadales(99.4),Xanthomonadaceae(99.4),Dyella(93.8)	k__Bacteria;p__Proteobacteria;c__Gammaproteobacteria;o__Xanthomonadales;f__Xanthomonadaceae;g__Dyella;s__	1,00
bc01_D07;OTUwell_2	Bacteria(100.0),"Proteobacteria"(100.0),Gammaproteobacteria(99.0),Xanthomonadales(99.0),Xanthomonadaceae(98.7),Dyella(86.9)	k__Bacteria;p__Proteobacteria;c__Gammaproteobacteria;o__Xanthomonadales;f__Xanthomonadaceae;g__Dyella;s__	1,00
bc01_D07;OTUwell_3	Bacteria(100.0),"Proteobacteria"(100.0),Gammaproteobacteria(99.0),Xanthomonadales(99.0),Xanthomonadaceae(98.6),Dyella(84.7)	k__Bacteria;p__Proteobacteria;c__Gammaproteobacteria;o__Xanthomonadales;f__Xanthomonadaceae;g__Dyella;s__	0,97
bc01_D07;OTUwell_4	Bacteria(100.0),"Proteobacteria"(100.0),Gammaproteobacteria(100.0),"Enterobacteriales"(98.9),Enterobacteriaceae(98.2),Enterobacter(92.4)	k__Bacteria;p__Proteobacteria;c__Gammaproteobacteria;o__Enterobacteriales;f__Enterobacteriaceae;g__;s__	0,95
bc01_D08;OTUwell_1	Bacteria(100.0),"Bacteroidetes"(100.0),"Sphingobacteria"(99.6),"Sphingobacteriales"(99.6),Chitinophagaceae(99.6),Chitinophaga(99.0)	k__Bacteria;p__Bacteroidetes;c__[Saprospirae];o__[Saprospirales];f__Chitinophagaceae;g__Chitinophaga;s__	1,00
bc01_D09;OTUwell_1	Bacteria(100.0),"Proteobacteria"(100.0),Alphaproteobacteria(98.7),Rhizobiales(98.7),Rhizobiaceae(98.7),Rhizobium(98.7)	k__Bacteria;p__Proteobacteria;c__Alphaproteobacteria;o__Rhizobiales;f__Rhizobiaceae;g__Rhizobium;s__	0,97
bc01_D10;OTUwell_1	Bacteria(100.0),"Proteobacteria"(100.0),Gammaproteobacteria(99.8),Xanthomonadales(99.5),Xanthomonadaceae(99.5),Dyella(98.0)	k__Bacteria;p__Proteobacteria;c__Gammaproteobacteria;o__Xanthomonadales;f__Xanthomonadaceae;g__Dyella;s__	1,00
bc01_D11;OTUwell_1	Bacteria(100.0),"Proteobacteria"(100.0),Alphaproteobacteria(99.6),Caulobacterales(99.6),Caulobacteraceae(99.6),Caulobacter(99.3)	k__Bacteria;p__Proteobacteria;c__Alphaproteobacteria;o__Caulobacterales;f__Caulobacteraceae;g__Caulobacter;s__	1,00
bc01_D11;OTUwell_2	Bacteria(100.0),"Proteobacteria"(100.0),Betaproteobacteria(97.4),Burkholderiales(97.4),Oxalobacteraceae(97.4),Herbaspirillum(92.4)	k__Bacteria;p__Proteobacteria;c__Betaproteobacteria;o__Burkholderiales;f__Oxalobacteraceae;g__;s__	0,83
bc01_D11;OTUwell_3	Bacteria(100.0),"Proteobacteria"(100.0),Alphaproteobacteria(98.9),Caulobacterales(98.9),Caulobacteraceae(98.9),Caulobacter(93.8)	k__Bacteria;p__Proteobacteria;c__Alphaproteobacteria;o__Caulobacterales;f__Caulobacteraceae;g__Caulobacter;s__	1,00
bc01_D12;OTUwell_1	Bacteria(100.0),"Proteobacteria"(100.0),Gammaproteobacteria(99.8),Xanthomonadales(99.5),Xanthomonadaceae(99.5),Dyella(98.0)	k__Bacteria;p__Proteobacteria;c__Gammaproteobacteria;o__Xanthomonadales;f__Xanthomonadaceae;g__Dyella;s__	1,00
bc01_D12;OTUwell_2	Bacteria(100.0),Firmicutes(100.0),Bacilli(99.3),Bacillales(99.3),Bacillaceae_1(99.0),Bacillus(99.0)	k__Bacteria;p__Firmicutes;c__Bacilli;o__Bacillales;f__Bacillaceae;g__;s__	0,95
bc01_E01;OTUwell_1	Bacteria(100.0),Firmicutes(100.0),Bacilli(99.7),Bacillales(99.5),Bacillaceae_1(99.2),Bacillus(99.2)	k__Bacteria;p__Firmicutes;c__Bacilli;o__Bacillales;f__Bacillaceae;g__Bacillus	1,00
bc01_E01;OTUwell_2	Bacteria(100.0),"Bacteroidetes"(100.0),"Sphingobacteria"(100.0),"Sphingobacteriales"(100.0),Chitinophagaceae(100.0),Chitinophaga(99.9)	k__Bacteria;p__Bacteroidetes;c__[Saprospirae];o__[Saprospirales];f__Chitinophagaceae;g__Chitinophaga;s__	1,00
bc01_E01;OTUwell_3	Bacteria(100.0),"Bacteroidetes"(100.0),"Sphingobacteria"(99.6),"Sphingobacteriales"(99.6),Chitinophagaceae(99.6),Chitinophaga(99.1)	k__Bacteria;p__Bacteroidetes;c__[Saprospirae];o__[Saprospirales];f__Chitinophagaceae;g__Chitinophaga;s__	1,00
bc01_E01;OTUwell_4	Bacteria(100.0),"Bacteroidetes"(100.0),"Sphingobacteria"(97.8),"Sphingobacteriales"(97.8),Sphingobacteriaceae(97.8),Mucilaginibacter(93.2)	k__Bacteria;p__Bacteroidetes;c__Sphingobacteriia;o__Sphingobacteriales;f__Sphingobacteriaceae;g__;s__	1,00
bc01_E02;OTUwell_1	Bacteria(100.0),"Proteobacteria"(100.0),Gammaproteobacteria(99.7),Xanthomonadales(99.0),Xanthomonadaceae(98.5),Lysobacter(93.8)	k__Bacteria;p__Proteobacteria;c__Gammaproteobacteria;o__Xanthomonadales;f__Xanthomonadaceae;g__Luteimonas;s__	1,00
bc01_E05;OTUwell_1	Bacteria(100.0),"Bacteroidetes"(100.0),"Sphingobacteria"(99.9),"Sphingobacteriales"(99.9),Chitinophagaceae(99.9),Chitinophaga(99.9)	k__Bacteria;p__Bacteroidetes;c__[Saprospirae];o__[Saprospirales];f__Chitinophagaceae;g__Chitinophaga;s__	1,00
bc01_E06;OTUwell_1	Bacteria(100.0),"Bacteroidetes"(100.0),"Sphingobacteria"(99.6),"Sphingobacteriales"(99.6),Chitinophagaceae(99.6),Chitinophaga(99.2)	k__Bacteria;p__Bacteroidetes;c__[Saprospirae];o__[Saprospirales];f__Chitinophagaceae;g__Chitinophaga;s__	1,00
bc01_E07;OTUwell_1	Bacteria(100.0),"Proteobacteria"(100.0),Betaproteobacteria(99.0),Burkholderiales(99.0),Comamonadaceae(97.8),Variovorax(95.9)	k__Bacteria;p__Proteobacteria;c__Betaproteobacteria;o__Burkholderiales;f__Comamonadaceae;g__Variovorax;s__paradoxus	0,95
bc01_E07;OTUwell_2	Bacteria(100.0),"Proteobacteria"(100.0),Betaproteobacteria(99.3),Burkholderiales(99.3),Burkholderiaceae(99.3),Burkholderia(99.0)	k__Bacteria;p__Proteobacteria;c__Betaproteobacteria;o__Burkholderiales;f__Burkholderiaceae;g__Burkholderia;s__	0,96
bc01_E07;OTUwell_3	Bacteria(100.0),"Proteobacteria"(100.0),Betaproteobacteria(99.5),Burkholderiales(99.4),Burkholderiaceae(99.4),Burkholderia(99.1)	k__Bacteria;p__Proteobacteria;c__Betaproteobacteria;o__Burkholderiales;f__Burkholderiaceae;g__Burkholderia;s__	1,00
bc01_E08;OTUwell_1	Bacteria(100.0),"Proteobacteria"(100.0),Betaproteobacteria(98.4),Burkholderiales(98.4),Comamonadaceae(97.5),Polaromonas(94.5)	k__Bacteria;p__Proteobacteria;c__Betaproteobacteria;o__Burkholderiales;f__Comamonadaceae;g__Polaromonas;s__	1,00
bc01_E11;OTUwell_1	Bacteria(100.0),"Proteobacteria"(100.0),Alphaproteobacteria(95.6),Rhizobiales(95.6),Rhizobiaceae(94.9),Rhizobium(86.9)	k__Bacteria;p__Proteobacteria;c__Alphaproteobacteria;o__Rhizobiales;f__Rhizobiaceae;g__Agrobacterium;s__	1,00
bc01_E11;OTUwell_2	Bacteria(100.0),"Proteobacteria"(100.0),Betaproteobacteria(98.8),Burkholderiales(98.8),Burkholderiaceae(98.6),Burkholderia(97.1)	k__Bacteria;p__Proteobacteria;c__Betaproteobacteria;o__Burkholderiales;f__Burkholderiaceae;g__Burkholderia;s__	0,93
bc01_E11;OTUwell_3	Bacteria(100.0),"Proteobacteria"(100.0),Betaproteobacteria(98.7),Burkholderiales(98.7),Comamonadaceae(97.2),Variovorax(93.2)	k__Bacteria;p__Proteobacteria;c__Betaproteobacteria;o__Burkholderiales;f__Comamonadaceae;g__Variovorax;s__paradoxus	0,84
bc01_E12;OTUwell_1	Bacteria(100.0),"Proteobacteria"(100.0),Betaproteobacteria(99.1),Burkholderiales(99.1),Burkholderiaceae(98.8),Burkholderia(98.0)	k__Bacteria;p__Proteobacteria;c__Betaproteobacteria;o__Burkholderiales;f__Burkholderiaceae;g__Burkholderia;s__	0,91
bc01_F01;OTUwell_1	Bacteria(100.0),"Proteobacteria"(100.0),Gammaproteobacteria(99.5),Xanthomonadales(99.3),Xanthomonadaceae(99.2),Fulvimonas(90.1)	k__Bacteria;p__Proteobacteria;c__Gammaproteobacteria;o__Xanthomonadales;f__Xanthomonadaceae;g__Dyella;s__	0,98
bc01_F01;OTUwell_2	Bacteria(100.0),"Bacteroidetes"(100.0),Flavobacteria(98.0),"Flavobacteriales"(98.0),Flavobacteriaceae(98.0),Flavobacterium(94.5)	k__Bacteria;p__Bacteroidetes;c__Flavobacteriia;o__Flavobacteriales;f__Flavobacteriaceae;g__Flavobacterium;s__	1,00
bc01_F03;OTUwell_1	Bacteria(100.0),"Proteobacteria"(100.0),Alphaproteobacteria(98.6),Rhodospirillales(98.6),Rhodospirillaceae(98.5),Inquilinus(98.3)	k__Bacteria;p__Proteobacteria;c__Alphaproteobacteria;o__Rhodospirillales;f__Rhodospirillaceae;g__Inquilinus;s__limosus	1,00
bc01_F04;OTUwell_1	Bacteria(100.0),"Proteobacteria"(100.0),Betaproteobacteria(99.1),Burkholderiales(99.1),Comamonadaceae(97.9),Variovorax(95.4)	k__Bacteria;p__Proteobacteria;c__Betaproteobacteria;o__Burkholderiales;f__Comamonadaceae;g__Variovorax;s__paradoxus	0,91
bc01_F06;OTUwell_1	Bacteria(100.0),"Proteobacteria"(100.0),Alphaproteobacteria(98.5),Rhizobiales(98.5),Rhizobiaceae(98.5),Ensifer(97.5)	k__Bacteria;p__Proteobacteria;c__Alphaproteobacteria;o__Rhizobiales;f__Rhizobiaceae;g__;s__	0,90
bc01_F07;OTUwell_1	Bacteria(100.0),"Proteobacteria"(100.0),Betaproteobacteria(99.0),Burkholderiales(99.0),Comamonadaceae(97.9),Variovorax(96.3)	k__Bacteria;p__Proteobacteria;c__Betaproteobacteria;o__Burkholderiales;f__Comamonadaceae	1,00
bc01_F07;OTUwell_2	Bacteria(100.0),"Proteobacteria"(100.0),Betaproteobacteria(98.6),Burkholderiales(98.6),Comamonadaceae(97.2),Variovorax(93.2)	k__Bacteria;p__Proteobacteria;c__Betaproteobacteria;o__Burkholderiales;f__Comamonadaceae;g__Variovorax	0,88
bc01_F08;OTUwell_1	Bacteria(100.0),"Proteobacteria"(100.0),Gammaproteobacteria(99.3),Xanthomonadales(99.2),Xanthomonadaceae(99.0),Dyella(89.1)	k__Bacteria;p__Proteobacteria;c__Gammaproteobacteria;o__Xanthomonadales;f__Xanthomonadaceae;g__Dyella;s__	1,00
bc01_F08;OTUwell_2	Bacteria(100.0),"Proteobacteria"(100.0),Alphaproteobacteria(99.3),Caulobacterales(99.3),Caulobacteraceae(99.3),Caulobacter(97.5)	k__Bacteria;p__Proteobacteria;c__Alphaproteobacteria;o__Caulobacterales;f__Caulobacteraceae;g__Caulobacter;s__	0,95
bc01_F09;OTUwell_1	Bacteria(100.0),"Proteobacteria"(100.0),Alphaproteobacteria(97.4),Rhizobiales(97.4),Rhizobiaceae(97.4),Rhizobium(95.4)	k__Bacteria;p__Proteobacteria;c__Alphaproteobacteria;o__Rhizobiales;f__Rhizobiaceae;g__Rhizobium	1,00
bc01_F10;OTUwell_1	Bacteria(100.0),"Bacteroidetes"(100.0),"Sphingobacteria"(99.3),"Sphingobacteriales"(99.3),Sphingobacteriaceae(99.3),Mucilaginibacter(99.3)	k__Bacteria;p__Bacteroidetes;c__Sphingobacteriia;o__Sphingobacteriales;f__Sphingobacteriaceae;g__;s__	1,00
bc01_F11;OTUwell_1	Bacteria(100.0),"Bacteroidetes"(100.0),"Sphingobacteria"(99.7),"Sphingobacteriales"(99.6),Chitinophagaceae(99.6),Chitinophaga(99.6)	k__Bacteria;p__Bacteroidetes;c__[Saprospirae];o__[Saprospirales];f__Chitinophagaceae;g__Chitinophaga;s__	1,00
bc01_F12;OTUwell_1	Bacteria(100.0),"Proteobacteria"(100.0),Alphaproteobacteria(98.9),Rhizobiales(98.9),Rhizobiaceae(98.9),Rhizobium(98.9)	k__Bacteria;p__Proteobacteria;c__Alphaproteobacteria;o__Rhizobiales;f__Rhizobiaceae;g__Rhizobium;s__	1,00
bc01_G01;OTUwell_1	Bacteria(100.0),"Proteobacteria"(100.0),Gammaproteobacteria(99.5),Xanthomonadales(99.3),Xanthomonadaceae(99.2),Fulvimonas(90.1)	k__Bacteria;p__Proteobacteria;c__Gammaproteobacteria;o__Xanthomonadales;f__Xanthomonadaceae;g__Dyella;s__	0,97
bc01_G01;OTUwell_2	Bacteria(100.0),"Bacteroidetes"(100.0),Flavobacteria(98.2),"Flavobacteriales"(98.2),Flavobacteriaceae(98.2),Flavobacterium(95.4)	k__Bacteria;p__Bacteroidetes;c__Flavobacteriia;o__Flavobacteriales;f__Flavobacteriaceae;g__Flavobacterium;s__	1,00
bc01_G01;OTUwell_3	Bacteria(100.0),"Proteobacteria"(100.0),Betaproteobacteria(99.4),Burkholderiales(99.4),Burkholderiaceae(99.4),Burkholderia(99.2)	k__Bacteria;p__Proteobacteria;c__Betaproteobacteria;o__Burkholderiales;f__Burkholderiaceae;g__Burkholderia;s__	0,88
bc01_G02;OTUwell_1	Bacteria(100.0),"Proteobacteria"(100.0),Gammaproteobacteria(99.5),Xanthomonadales(99.3),Xanthomonadaceae(99.3),Fulvimonas(90.8)	k__Bacteria;p__Proteobacteria;c__Gammaproteobacteria;o__Xanthomonadales;f__Xanthomonadaceae;g__Dyella;s__	0,90
bc01_G02;OTUwell_2	Bacteria(100.0),"Proteobacteria"(100.0),Alphaproteobacteria(98.7),Rhizobiales(98.7),Xanthobacteraceae(96.7),Starkeya(90.8)	k__Bacteria;p__Proteobacteria;c__Alphaproteobacteria;o__Rhizobiales;f__Xanthobacteraceae;g__;s__	1,00
bc01_G02;OTUwell_3	Bacteria(100.0),"Bacteroidetes"(100.0),Flavobacteria(97.6),"Flavobacteriales"(97.6),Flavobacteriaceae(97.6),Flavobacterium(90.8)	k__Bacteria;p__Bacteroidetes;c__Flavobacteriia;o__Flavobacteriales;f__Flavobacteriaceae;g__Flavobacterium;s__	1,00
bc01_G02;OTUwell_4	Bacteria(100.0),"Bacteroidetes"(100.0),"Sphingobacteria"(99.9),"Sphingobacteriales"(99.9),Chitinophagaceae(99.9),Chitinophaga(99.9)	k__Bacteria;p__Bacteroidetes;c__[Saprospirae];o__[Saprospirales];f__Chitinophagaceae;g__Chitinophaga;s__	1,00
bc01_G03;OTUwell_1	Bacteria(100.0),"Bacteroidetes"(100.0),"Sphingobacteria"(99.6),"Sphingobacteriales"(99.6),Chitinophagaceae(99.6),Chitinophaga(99.2)	k__Bacteria;p__Bacteroidetes;c__[Saprospirae];o__[Saprospirales];f__Chitinophagaceae;g__Chitinophaga;s__	1,00
bc01_G04;OTUwell_1	Bacteria(100.0),"Bacteroidetes"(100.0),"Sphingobacteria"(100.0),"Sphingobacteriales"(100.0),Cytophagaceae(99.5),Dyadobacter(99.5)	k__Bacteria;p__Bacteroidetes;c__Cytophagia;o__Cytophagales;f__Cytophagaceae;g__Dyadobacter;s__	1,00
bc01_G06;OTUwell_1	Bacteria(100.0),"Proteobacteria"(100.0),Betaproteobacteria(99.5),Burkholderiales(99.5),Burkholderiaceae(99.5),Burkholderia(99.5)	k__Bacteria;p__Proteobacteria;c__Betaproteobacteria;o__Burkholderiales;f__Burkholderiaceae;g__Burkholderia;s__	1,00
bc01_G06;OTUwell_2	Bacteria(100.0),"Proteobacteria"(100.0),Alphaproteobacteria(98.8),Rhizobiales(98.8),Rhizobiaceae(98.8),Rhizobium(98.8)	k__Bacteria;p__Proteobacteria;c__Alphaproteobacteria;o__Rhizobiales;f__Rhizobiaceae;g__Rhizobium;s__	0,98
bc01_G07;OTUwell_1	Bacteria(100.0),"Proteobacteria"(100.0),Gammaproteobacteria(99.6),Xanthomonadales(99.4),Xanthomonadaceae(99.4),Dyella(93.8)	k__Bacteria;p__Proteobacteria;c__Gammaproteobacteria;o__Xanthomonadales;f__Xanthomonadaceae;g__Dyella;s__	1,00
bc01_G07;OTUwell_2	Bacteria(100.0),"Actinobacteria"(100.0),Actinobacteria(100.0),Actinomycetales(99.6),Microbacteriaceae(99.4),Microbacterium(98.9)	k__Bacteria;p__Actinobacteria;c__Actinobacteria;o__Actinomycetales;f__Microbacteriaceae;g__Microbacterium;s__	0,93
bc01_G07;OTUwell_3	Bacteria(100.0),"Proteobacteria"(100.0),Gammaproteobacteria(99.2),Xanthomonadales(99.1),Xanthomonadaceae(98.8),Dyella(86.9)	k__Bacteria;p__Proteobacteria;c__Gammaproteobacteria;o__Xanthomonadales;f__Xanthomonadaceae;g__Dyella;s__	1,00
bc01_G07;OTUwell_4	Bacteria(100.0),"Proteobacteria"(100.0),Gammaproteobacteria(99.2),Xanthomonadales(99.2),Xanthomonadaceae(98.9),Dyella(88.0)	k__Bacteria;p__Proteobacteria;c__Gammaproteobacteria;o__Xanthomonadales;f__Xanthomonadaceae;g__Dyella;s__	1,00

bc01_G08;OTUwell_1	Bacteria(100.0),"Proteobacteria"(100.0),Alphaproteobacteria(98.9),Rhizobiales(98.9),Rhizobiaceae(98.9),Rhizobium(98.9)	k__Bacteria;p__Proteobacteria;c__Alphaproteobacteria;o__Rhizobiales;f__Rhizobiaceae;g__Rhizobium;s__	0,99
bc01_G09;OTUwell_1	Bacteria(100.0),"Proteobacteria"(100.0),Alphaproteobacteria(98.8),Rhizobiales(98.8),Rhizobiaceae(98.8),Rhizobium(98.8)	k__Bacteria;p__Proteobacteria;c__Alphaproteobacteria;o__Rhizobiales;f__Rhizobiaceae;g__Rhizobium;s__	1,00
bc01_G10;OTUwell_1	Bacteria(100.0),"Bacteroidetes"(100.0),"Sphingobacteria"(98.2),"Sphingobacteriales"(98.2),Sphingobacteriaceae(98.2),Mucilaginibacter(95.9)	k__Bacteria;p__Bacteroidetes;c__Sphingobacteriia;o__Sphingobacteriales;f__Sphingobacteriaceae;g__;s__	1,00
bc01_G10;OTUwell_2	Bacteria(100.0),"Proteobacteria"(100.0),Betaproteobacteria(97.3),Burkholderiales(95.9),Burkholderiaceae(94.9),Chitinimonas(86.9)	k__Bacteria;p__Proteobacteria;c__Betaproteobacteria;o__Neisseriales;f__Neisseriaceae;g__;s__	1,00
bc01_G10;OTUwell_3	Bacteria(100.0),"Bacteroidetes"(100.0),"Sphingobacteria"(97.2),"Sphingobacteriales"(97.2),Sphingobacteriaceae(97.2),Mucilaginibacter(90.1)	k__Bacteria;p__Bacteroidetes;c__Sphingobacteriia;o__Sphingobacteriales;f__Sphingobacteriaceae;g__;s__	1,00
bc01_G10;OTUwell_4	Bacteria(100.0),"Actinobacteria"(100.0),Actinobacteria(100.0),Actinomycetales(99.6),Microbacteriaceae(99.4),Microbacterium(98.9)	k__Bacteria;p__Actinobacteria;c__Actinobacteria;o__Actinomycetales;f__Microbacteriaceae;g__Microbacterium;s__	0,97
bc01_G11;OTUwell_1	Bacteria(100.0),"Proteobacteria"(100.0),Alphaproteobacteria(98.9),Rhizobiales(98.9),Rhizobiaceae(98.9),Rhizobium(98.9)	k__Bacteria;p__Proteobacteria;c__Alphaproteobacteria;o__Rhizobiales;f__Rhizobiaceae;g__Rhizobium;s__	1,00
bc01_G11;OTUwell_2	Bacteria(100.0),"Proteobacteria"(100.0),Alphaproteobacteria(98.6),Rhizobiales(98.6),Rhizobiaceae(98.6),Rhizobium(98.6)	k__Bacteria;p__Proteobacteria;c__Alphaproteobacteria;o__Rhizobiales;f__Rhizobiaceae	1,00
bc01_G12;OTUwell_1	Bacteria(100.0),"Proteobacteria"(100.0),Alphaproteobacteria(98.8),Rhizobiales(98.8),Rhizobiaceae(98.8),Rhizobium(98.8)	k__Bacteria;p__Proteobacteria;c__Alphaproteobacteria;o__Rhizobiales;f__Rhizobiaceae;g__Rhizobium;s__	1,00
bc01_H02;OTUwell_1	Bacteria(100.0),"Proteobacteria"(100.0),Gammaproteobacteria(99.5),Xanthomonadales(99.3),Xanthomonadaceae(99.3),Fulvimonas(90.8)	k__Bacteria;p__Proteobacteria;c__Gammaproteobacteria;o__Xanthomonadales;f__Xanthomonadaceae;g__Dyella;s__	0,97
bc01_H02;OTUwell_2	Bacteria(100.0),"Proteobacteria"(100.0),Alphaproteobacteria(99.3),Rhizobiales(99.3),Hyphomicrobiaceae(99.3),Hyphomicrobium(98.9)	k__Bacteria;p__Proteobacteria;c__Alphaproteobacteria;o__Rhizobiales;f__Hyphomicrobiaceae;g__Hyphomicrobium;s__	1,00
bc01_H02;OTUwell_3	Bacteria(100.0),"Proteobacteria"(100.0),Gammaproteobacteria(98.7),Xanthomonadales(98.7),Xanthomonadaceae(98.2),Fulvimonas(78.6)	k__Bacteria;p__Proteobacteria;c__Gammaproteobacteria;o__Xanthomonadales;f__Xanthomonadaceae;g__Dyella;s__	0,98
bc01_H02;OTUwell_4	Bacteria(100.0),"Proteobacteria"(100.0),Gammaproteobacteria(98.4),Xanthomonadales(98.4),Xanthomonadaceae(97.7),Fulvimonas(72.5)	k__Bacteria;p__Proteobacteria;c__Gammaproteobacteria;o__Xanthomonadales;f__Xanthomonadaceae;g__Dyella;s__	0,99
bc01_H03;OTUwell_1	Bacteria(100.0),"Acidobacteria"(90.6),Holophagae(64.6),Holophagales(54.4),Holophagaceae(43.1),Holophaga(43.1)	k__Bacteria;p__Acidobacteria;c__Acidobacteriia;o__Acidobacteriales;f__Acidobacteriaceae;g__Terriglobus;s__	0,87
bc01_H04;OTUwell_1	Bacteria(100.0),"Actinobacteria"(100.0),Actinobacteria(99.2),Actinomycetales(99.2),Micrococcaceae(97.0),Arthrobacter(88.0)	k__Bacteria;p__Actinobacteria;c__Actinobacteria;o__Actinomycetales;f__Micrococcaceae;g__;s__	0,98
bc01_H04;OTUwell_2	Bacteria(100.0),"Actinobacteria"(100.0),Actinobacteria(100.0),Actinomycetales(99.6),Microbacteriaceae(99.2),Microbacterium(97.7)	k__Bacteria;p__Actinobacteria;c__Actinobacteria;o__Actinomycetales;f__Microbacteriaceae;g__Microbacterium;s__	0,90
bc01_H04;OTUwell_3	Bacteria(100.0),"Bacteroidetes"(100.0),"Sphingobacteria"(98.1),"Sphingobacteriales"(98.1),Sphingobacteriaceae(98.1),Mucilaginibacter(95.9)	k__Bacteria;p__Bacteroidetes;c__Sphingobacteriia;o__Sphingobacteriales;f__Sphingobacteriaceae;g__;s__	1,00
bc01_H06;OTUwell_1	Bacteria(100.0),"Proteobacteria"(100.0),Betaproteobacteria(99.1),Burkholderiales(99.1),Comamonadaceae(97.7),Variovorax(95.4)	k__Bacteria;p__Proteobacteria;c__Betaproteobacteria;o__Burkholderiales;f__Comamonadaceae;g__Variovorax;s__paradoxus	0,91
bc01_H08;OTUwell_1	Bacteria(100.0),"Proteobacteria"(100.0),Gammaproteobacteria(99.6),Xanthomonadales(99.4),Xanthomonadaceae(99.3),Dyella(93.2)	k__Bacteria;p__Proteobacteria;c__Gammaproteobacteria;o__Xanthomonadales;f__Xanthomonadaceae;g__Dyella;s__	1,00
bc01_H09;OTUwell_1	Bacteria(100.0),"Proteobacteria"(100.0),Gammaproteobacteria(99.6),Xanthomonadales(99.5),Xanthomonadaceae(99.5),Dyella(94.5)	k__Bacteria;p__Proteobacteria;c__Gammaproteobacteria;o__Xanthomonadales;f__Xanthomonadaceae;g__Dyella;s__	1,00
bc01_H10;OTUwell_1	Bacteria(100.0),"Proteobacteria"(100.0),Gammaproteobacteria(100.0),"Enterobacteriales"(99.4),Enterobacteriaceae(99.4),Escherichia/Shigella(98.9)	k__Bacteria;p__Proteobacteria;c__Gammaproteobacteria;o__Enterobacteriales;f__Enterobacteriaceae;g__;s__	1,00
bc01_H10;OTUwell_2	Bacteria(100.0),"Proteobacteria"(100.0),Gammaproteobacteria(99.6),"Enterobacteriales"(98.8),Enterobacteriaceae(98.2),Escherichia/Shigella(92.4)	k__Bacteria;p__Proteobacteria;c__Gammaproteobacteria;o__Enterobacteriales;f__Enterobacteriaceae;g__;s__	1,00
bc01_H11;OTUwell_1	Bacteria(100.0),"Proteobacteria"(100.0),Gammaproteobacteria(100.0),"Enterobacteriales"(99.5),Enterobacteriaceae(99.5),Escherichia/Shigella(99.0)	k__Bacteria;p__Proteobacteria;c__Gammaproteobacteria;o__Enterobacteriales;f__Enterobacteriaceae;g__;s__	1,00
bc01_H12;OTUwell_1	Bacteria(100.0),"Proteobacteria"(100.0),Gammaproteobacteria(100.0),"Enterobacteriales"(99.5),Enterobacteriaceae(99.5),Escherichia/Shigella(99.0)	k__Bacteria;p__Proteobacteria;c__Gammaproteobacteria;o__Enterobacteriales;f__Enterobacteriaceae;g__;s__	1,00
bc02_A01;OTUwell_1	Bacteria(100.0),"Proteobacteria"(100.0),Alphaproteobacteria(98.9),Rhizobiales(98.9),Rhizobiaceae(98.9),Rhizobium(98.9)	k__Bacteria;p__Proteobacteria;c__Alphaproteobacteria;o__Rhizobiales;f__Rhizobiaceae;g__Rhizobium;s__	1,00
bc02_A01;OTUwell_2	Bacteria(100.0),"Bacteroidetes"(100.0),"Sphingobacteria"(98.4),"Sphingobacteriales"(98.4),Sphingobacteriaceae(98.4),Pedobacter(98.0)	k__Bacteria;p__Bacteroidetes;c__Sphingobacteriia;o__Sphingobacteriales;f__Sphingobacteriaceae;g__Pedobacter;s__	1,00
bc02_A02;OTUwell_1	Bacteria(100.0),"Proteobacteria"(100.0),Alphaproteobacteria(98.9),Rhizobiales(98.9),Rhizobiaceae(98.9),Rhizobium(98.9)	k__Bacteria;p__Proteobacteria;c__Alphaproteobacteria;o__Rhizobiales;f__Rhizobiaceae;g__Rhizobium;s__	1,00
bc02_A03;OTUwell_1	Bacteria(100.0),Firmicutes(100.0),Bacilli(99.6),Bacillales(99.5),Bacillaceae_1(99.4),Bacillus(99.4)	k__Bacteria;p__Firmicutes;c__Bacilli;o__Bacillales;f__Bacillaceae;g__;s__	1,00
bc02_A03;OTUwell_2	Bacteria(100.0),Firmicutes(100.0),Bacilli(99.1),Bacillales(99.0),Bacillaceae_1(97.8),Bacillus(96.6)	k__Bacteria;p__Firmicutes;c__Bacilli;o__Bacillales;f__Bacillaceae;g__Bacillus;s__	1,00
bc02_A04;OTUwell_1	Bacteria(100.0),"Proteobacteria"(100.0),Betaproteobacteria(99.2),Burkholderiales(99.2),Comamonadaceae(98.2),Variovorax(96.6)	k__Bacteria;p__Proteobacteria;c__Betaproteobacteria;o__Burkholderiales;f__Comamonadaceae;g__Variovorax;s__paradoxus	0,91
bc02_A07;OTUwell_1	Bacteria(100.0),"Proteobacteria"(100.0),Gammaproteobacteria(100.0),"Enterobacteriales"(99.1),Enterobacteriaceae(98.8),Enterobacter(95.4)	k__Bacteria;p__Proteobacteria;c__Gammaproteobacteria;o__Enterobacteriales;f__Enterobacteriaceae;g__;s__	0,88
bc02_A07;OTUwell_2	Bacteria(100.0),"Proteobacteria"(100.0),Alphaproteobacteria(98.6),Rhizobiales(98.6),Rhizobiaceae(98.6),Rhizobium(98.6)	k__Bacteria;p__Proteobacteria;c__Alphaproteobacteria;o__Rhizobiales;f__Rhizobiaceae;g__Rhizobium;s__	1,00
bc02_A09;OTUwell_1	Bacteria(100.0),Firmicutes(100.0),Bacilli(99.6),Bacillales(99.5),Bacillaceae_1(99.4),Bacillus(99.4)	k__Bacteria;p__Firmicutes;c__Bacilli;o__Bacillales;f__Bacillaceae;g__;s__	0,94
bc02_A09;OTUwell_2	Bacteria(100.0),"Bacteroidetes"(100.0),"Sphingobacteria"(97.9),"Sphingobacteriales"(97.9),Sphingobacteriaceae(97.9),Mucilaginibacter(94.5)	k__Bacteria;p__Bacteroidetes;c__Sphingobacteriia;o__Sphingobacteriales;f__Sphingobacteriaceae;g__;s__	1,00
bc02_A10;OTUwell_1	Bacteria(100.0),Firmicutes(100.0),Bacilli(99.6),Bacillales(99.5),Bacillaceae_1(99.4),Bacillus(99.4)	k__Bacteria;p__Firmicutes;c__Bacilli;o__Bacillales;f__Bacillaceae;g__;s__	1,00
bc02_A10;OTUwell_2	Bacteria(100.0),"Proteobacteria"(100.0),Betaproteobacteria(99.0),Burkholderiales(99.0),Comamonadaceae(97.8),Variovorax(95.9)	k__Bacteria;p__Proteobacteria;c__Betaproteobacteria;o__Burkholderiales;f__Comamonadaceae;g__Variovorax;s__paradoxus	0,88
bc02_A11;OTUwell_1	Bacteria(100.0),"Proteobacteria"(100.0),Alphaproteobacteria(98.8),Rhizobiales(98.8),Rhizobiaceae(98.8),Rhizobium(98.8)	k__Bacteria;p__Proteobacteria;c__Alphaproteobacteria;o__Rhizobiales;f__Rhizobiaceae;g__Rhizobium;s__	1,00
bc02_A11;OTUwell_2	Bacteria(100.0),"Actinobacteria"(100.0),Actinobacteria(100.0),Actinomycetales(99.6),Micrococcineae_incertae_sedis(96.2),Luteimicrobium(91.7)	k__Bacteria;p__Actinobacteria;c__Actinobacteria;o__Actinomycetales;f__Promicromonosporaceae	0,80
bc02_A11;OTUwell_3	Bacteria(100.0),"Proteobacteria"(100.0),Gammaproteobacteria(99.6),Xanthomonadales(99.4),Xanthomonadaceae(99.4),Dyella(92.4)	k__Bacteria;p__Proteobacteria;c__Gammaproteobacteria;o__Xanthomonadales;f__Xanthomonadaceae;g__Dyella;s__	1,00
bc02_B01;OTUwell_1	Bacteria(100.0),"Proteobacteria"(100.0),Betaproteobacteria(99.0),Burkholderiales(99.0),Burkholderiaceae(98.7),Burkholderia(97.7)	k__Bacteria;p__Proteobacteria;c__Betaproteobacteria;o__Burkholderiales;f__Burkholderiaceae;g__Burkholderia	0,80
bc02_B01;OTUwell_2	Bacteria(100.0),"Proteobacteria"(100.0),Betaproteobacteria(97.9),Burkholderiales(97.9),Burkholderiaceae(97.0),Burkholderia(89.1)	k__Bacteria;p__Proteobacteria;c__Betaproteobacteria;o__Burkholderiales;f__Burkholderiaceae	1,00
bc02_B01;OTUwell_3	Bacteria(100.0),"Proteobacteria"(100.0),Alphaproteobacteria(98.7),Rhizobiales(98.7),Rhizobiaceae(98.7),Rhizobium(98.7)	k__Bacteria;p__Proteobacteria;c__Alphaproteobacteria;o__Rhizobiales;f__Rhizobiaceae;g__Rhizobium;s__	1,00
bc02_B03;OTUwell_1	Bacteria(100.0),"Proteobacteria"(100.0),Alphaproteobacteria(98.7),Rhizobiales(98.7),Rhizobiaceae(98.7),Rhizobium(98.7)	k__Bacteria;p__Proteobacteria;c__Alphaproteobacteria;o__Rhizobiales;f__Rhizobiaceae;g__Rhizobium;s__	1,00
bc02_B03;OTUwell_2	Bacteria(100.0),"Proteobacteria"(100.0),Betaproteobacteria(99.2),Burkholderiales(99.2),Burkholderiaceae(99.2),Burkholderia(98.9)	k__Bacteria;p__Proteobacteria;c__Betaproteobacteria;o__Burkholderiales;f__Burkholderiaceae;g__Burkholderia;s__	0,98
bc02_B03;OTUwell_3	Bacteria(100.0),"Proteobacteria"(100.0),Alphaproteobacteria(97.8),Rhizobiales(97.8),Rhizobiaceae(97.4),Rhizobium(95.9)	k__Bacteria;p__Proteobacteria;c__Alphaproteobacteria;o__Rhizobiales;f__Rhizobiaceae;g__Rhizobium;s__	0,91
bc02_B04;OTUwell_1	Bacteria(100.0),"Proteobacteria"(100.0),Gammaproteobacteria(99.5),Xanthomonadales(99.3),Xanthomonadaceae(99.3),Dyella(92.4)	k__Bacteria;p__Proteobacteria;c__Gammaproteobacteria;o__Xanthomonadales;f__Xanthomonadaceae;g__Dyella;s__	1,00
bc02_B05;OTUwell_1	Bacteria(100.0),"Proteobacteria"(100.0),Alphaproteobacteria(98.2),Rhizobiales(98.2),Rhizobiaceae(98.2),Rhizobium(98.2)	k__Bacteria;p__Proteobacteria;c__Alphaproteobacteria;o__Rhizobiales;f__Rhizobiaceae;g__Rhizobium;s__	0,95
bc02_B07;OTUwell_1	Bacteria(100.0),Firmicutes(100.0),Bacilli(99.7),Bacillales(99.5),Bacillaceae_1(99.4),Bacillus(99.4)	k__Bacteria;p__Firmicutes;c__Bacilli;o__Bacillales;f__Bacillaceae;g__;s__	0,98
bc02_B07;OTUwell_2	Bacteria(100.0),"Proteobacteria"(100.0),Betaproteobacteria(99.0),Burkholderiales(99.0),Comamonadaceae(97.5),Variovorax(95.0)	k__Bacteria;p__Proteobacteria;c__Betaproteobacteria;o__Burkholderiales;f__Comamonadaceae;g__Variovorax;s__paradoxus	0,87
bc02_B07;OTUwell_3	Bacteria(100.0),"Bacteroidetes"(100.0),"Sphingobacteria"(98.2),"Sphingobacteriales"(98.2),Sphingobacteriaceae(98.2),Mucilaginibacter(96.3)	k__Bacteria;p__Bacteroidetes;c__Sphingobacteriia;o__Sphingobacteriales;f__Sphingobacteriaceae;g__;s__	1,00
bc02_B08;OTUwell_1	Bacteria(100.0),Firmicutes(100.0),Bacilli(99.7),Bacillales(99.5),Bacillaceae_1(99.4),Bacillus(99.4)	k__Bacteria;p__Firmicutes;c__Bacilli;o__Bacillales;f__Bacillaceae;g__;s__	0,99
bc02_B08;OTUwell_2	Bacteria(100.0),"Proteobacteria"(100.0),Gammaproteobacteria(99.6),Xanthomonadales(99.5),Xanthomonadaceae(99.5),Dyella(95.9)	k__Bacteria;p__Proteobacteria;c__Gammaproteobacteria;o__Xanthomonadales;f__Xanthomonadaceae;g__Dyella;s__	1,00
bc02_B09;OTUwell_1	Bacteria(100.0),"Proteobacteria"(100.0),Gammaproteobacteria(99.7),Xanthomonadales(99.5),Xanthomonadaceae(99.5),Dyella(97.5)	k__Bacteria;p__Proteobacteria;c__Gammaproteobacteria;o__Xanthomonadales;f__Xanthomonadaceae;g__Dyella;s__	1,00
bc02_B09;OTUwell_2	Bacteria(100.0),Firmicutes(100.0),Bacilli(99.6),Bacillales(99.5),Bacillaceae_1(99.4),Bacillus(99.4)	k__Bacteria;p__Firmicutes;c__Bacilli;o__Bacillales;f__Bacillaceae;g__;s__	0,98
bc02_B10;OTUwell_1	Bacteria(100.0),Firmicutes(100.0),Bacilli(99.7),Bacillales(99.5),Bacillaceae_1(99.4),Bacillus(99.4)	k__Bacteria;p__Firmicutes;c__Bacilli;o__Bacillales;f__Bacillaceae;g__;s__	1,00
bc02_B10;OTUwell_2	Bacteria(100.0),"Proteobacteria"(100.0),Betaproteobacteria(99.0),Burkholderiales(99.0),Burkholderiaceae(98.7),Burkholderia(97.7)	k__Bacteria;p__Proteobacteria;c__Betaproteobacteria;o__Burkholderiales;f__Burkholderiaceae;g__Burkholderia;s__	0,88
bc02_B10;OTUwell_3	Bacteria(100.0),Firmicutes(100.0),Bacilli(99.6),Bacillales(99.5),Bacillaceae_1(99.3),Bacillus(99.3)	k__Bacteria;p__Firmicutes;c__Bacilli	1,00
bc02_B12;OTUwell_1	Bacteria(100.0),Firmicutes(100.0),Bacilli(99.7),Bacillales(99.5),Bacillaceae_1(99.3),Bacillus(99.3)	k__Bacteria;p__Firmicutes;c__Bacilli;o__Bacillales;f__Bacillaceae;g__Bacillus	1,00
bc02_B12;OTUwell_2	Bacteria(100.0),"Proteobacteria"(100.0),Alphaproteobacteria(98.5),Rhizobiales(98.5),Rhizobiaceae(98.5),Ensifer(97.5)	k__Bacteria;p__Proteobacteria;c__Alphaproteobacteria;o__Rhizobiales;f__Rhizobiaceae;g__;s__	0,93
bc02_B12;OTUwell_3	Bacteria(100.0),Firmicutes(100.0),Bacilli(99.0),Bacillales(99.0),Bacillaceae_1(98.2),Bacillus(97.7)	k__Bacteria;p__Firmicutes;c__Bacilli;o__Bacillales;f__Bacillaceae	1,00
bc02_C01;OTUwell_1	Bacteria(100.0),Firmicutes(100.0),Bacilli(99.7),Bacillales(99.5),Bacillaceae_1(99.4),Bacillus(99.4)	k__Bacteria;p__Firmicutes;c__Bacilli;o__Bacillales;f__Bacillaceae;g__;s__	0,99
bc02_C01;OTUwell_2	Bacteria(100.0),"Proteobacteria"(100.0),Alphaproteobacteria(98.9),Rhizobiales(98.9),Rhizobiaceae(98.9),Rhizobium(98.9)	k__Bacteria;p__Proteobacteria;c__Alphaproteobacteria;o__Rhizobiales;f__Rhizobiaceae;g__Rhizobium;s__	1,00
bc02_C01;OTUwell_3	Bacteria(100.0),Firmicutes(100.0),Bacilli(98.2),Bacillales(98.1),Bacillaceae_1(94.9),Bacillus(85.8)	k__Bacteria;p__Firmicutes;c__Bacilli;o__Bacillales;f__Bacillaceae;g__Bacillus;s__	0,95
bc02_C01;OTUwell_4	Bacteria(100.0),"Proteobacteria"(100.0),Betaproteobacteria(99.3),Burkholderiales(99.3),Burkholderiaceae(99.3),Wautersia(92.4)	k__Bacteria;p__Proteobacteria;c__Betaproteobacteria;o__Burkholderiales;f__Oxalobacteraceae;g__Cupriavidus;s__	1,00
bc02_C01;OTUwell_5	Bacteria(100.0),"Proteobacteria"(100.0),Betaproteobacteria(98.6),Burkholderiales(98.6),Burkholderiaceae(98.6),Wautersia(86.9)	k__Bacteria;p__Proteobacteria;c__Betaproteobacteria;o__Burkholderiales;f__Oxalobacteraceae;g__Cupriavidus;s__	1,00
bc02_C01;OTUwell_6	Bacteria(100.0),Firmicutes(100.0),Bacilli(98.5),Bacillales(98.4),Bacillaceae_1(95.9),Bacillus(89.1)	k__Bacteria;p__Firmicutes;c__Bacilli;o__Bacillales;f__Bacillaceae;g__Bacillus;s__	0,93
bc02_C01;OTUwell_7	Bacteria(100.0),Firmicutes(100.0),Bacilli(99.4),Bacillales(99.3),Bacillaceae_1(98.8),Bacillus(98.8)	k__Bacteria;p__Firmicutes;c__Bacilli;o__Bacillales;f__Bacillaceae;g__;s__	0,94
bc02_C01;OTUwell_8	Bacteria(100.0),Firmicutes(100.0),Bacilli(99.1),Bacillales(99.0),Bacillaceae_1(97.7),Bacillus(96.3)	k__Bacteria;p__Firmicutes;c__Bacilli;o__Bacillales;f__Bacillaceae;g__Bacillus;s__	0,89
bc02_C01;OTUwell_9	Bacteria(100.0),Firmicutes(100.0),Bacilli(99.5),Bacillales(99.3),Bacillaceae_1(98.4),Bacillus(98.4)	k__Bacteria;p__Firmicutes;c__Bacilli;o__Bacillales;f__Bacillaceae;g__Bacillus;s__	0,81
bc02_C02;OTUwell_1	Bacteria(100.0),Firmicutes(100.0),Bacilli(99.7),Bacillales(99.5),Bacillaceae_1(99.4),Bacillus(99.4)	k__Bacteria;p__Firmicutes;c__Bacilli;o__Bacillales;f__Bacillaceae;g__;s__	0,98
bc02_C02;OTUwell_2	Bacteria(100.0),"Proteobacteria"(100.0),Gammaproteobacteria(99.3),Xanthomonadales(97.9),Xanthomonadaceae(96.5),Pseudoxanthomonas(90.1)	k__Bacteria;p__Proteobacteria;c__Gammaproteobacteria;o__Xanthomonadales;f__Xanthomonadaceae	1,00
bc02_C02;OTUwell_3	Bacteria(100.0),"Proteobacteria"(100.0),Betaproteobacteria(97.6),Burkholderiales(96.8),Burkholderiaceae(95.7),Chitinimonas(89.1)	k__Bacteria;p__Proteobacteria;c__Betaproteobacteria;o__Neisseriales;f__Neisseriaceae;g__;s__	1,00
bc02_C02;OTUwell_4	Bacteria(100.0),"Proteobacteria"(100.0),Gammaproteobacteria(99.4),Xanthomonadales(97.7),Xanthomonadaceae(96.2),Pseudoxanthomonas(88.0)	k__Bacteria;p__Proteobacteria;c__Gammaproteobacteria;o__Xanthomonadales;f__Xanthomonadaceae	1,00
bc02_C02;OTUwell_5	Bacteria(100.0),"Proteobacteria"(100.0),Alphaproteobacteria(98.9),Rhizobiales(98.9),Rhizobiaceae(98.9),Rhizobium(98.9)	k__Bacteria;p__Proteobacteria;c__Alphaproteobacteria;o__Rhizobiales;f__Rhizobiaceae;g__Rhizobium;s__	1,00

bc02_C03;OTUwell_1	Bacteria(100.0),"Proteobacteria"(100.0),Alphaproteobacteria(98.0),Rhizobiales(98.0),Rhizobiaceae(98.0),Rhizobium(97.7)	k__Bacteria;p__Proteobacteria;c__Alphaproteobacteria;o__Rhizobiales;f__Rhizobiaceae;g__Rhizobium;s__	1,00
bc02_C06;OTUwell_1	Bacteria(100.0),Firmicutes(100.0),Bacilli(99.6),Bacillales(99.3),Bacillaceae_1(98.7),Bacillus(98.7)	k__Bacteria;p__Firmicutes;c__Bacilli;o__Bacillales;f__Bacillaceae;g__Bacillus	1,00
bc02_C06;OTUwell_2	Bacteria(100.0),"Proteobacteria"(100.0),Gammaproteobacteria(99.7),Xanthomonadales(99.5),Xanthomonadaceae(99.5),Dyella(97.7)	k__Bacteria;p__Proteobacteria;c__Gammaproteobacteria;o__Xanthomonadales;f__Xanthomonadaceae;g__Dyella;s__	0,98
bc02_C07;OTUwell_1	Bacteria(100.0),Firmicutes(100.0),Bacilli(99.7),Bacillales(99.5),Bacillaceae_1(99.4),Bacillus(99.4)	k__Bacteria;p__Firmicutes;c__Bacilli;o__Bacillales;f__Bacillaceae;g__;s__	1,00
bc02_C07;OTUwell_2	Bacteria(100.0),"Actinobacteria"(100.0),Actinobacteria(100.0),Actinomycetales(99.7),Microbacteriaceae(99.5),Curtobacterium(99.4)	k__Bacteria;p__Actinobacteria;c__Actinobacteria;o__Actinomycetales;f__Microbacteriaceae;g__Curtobacterium;s__	1,00
bc02_C09;OTUwell_1	Bacteria(100.0),"Proteobacteria"(100.0),Alphaproteobacteria(98.6),Sphingomonadales(96.2),Sphingomonadaceae(95.2),Sphingomonas(86.9)	k__Bacteria;p__Proteobacteria;c__Alphaproteobacteria;o__Sphingomonadales;f__Sphingomonadaceae;g__Sphingomonas;s__wittichii	1,00
bc02_C09;OTUwell_2	Bacteria(100.0),"Bacteroidetes"(100.0),"Sphingobacteria"(98.6),"Sphingobacteriales"(98.6),Sphingobacteriaceae(98.6),Pedobacter(98.6)	k__Bacteria;p__Bacteroidetes;c__Sphingobacteriia;o__Sphingobacteriales;f__Sphingobacteriaceae;g__Pedobacter;s__	1,00
bc02_C10;OTUwell_1	Bacteria(100.0),"Proteobacteria"(100.0),Gammaproteobacteria(99.8),Xanthomonadales(99.5),Xanthomonadaceae(99.5),Dyella(98.0)	k__Bacteria;p__Proteobacteria;c__Gammaproteobacteria;o__Xanthomonadales;f__Xanthomonadaceae;g__Dyella;s__	0,99
bc02_C10;OTUwell_2	Bacteria(100.0),Firmicutes(100.0),Bacilli(99.7),Bacillales(99.5),Bacillaceae_1(99.4),Bacillus(99.4)	k__Bacteria;p__Firmicutes;c__Bacilli;o__Bacillales;f__Bacillaceae;g__;s__	0,99
bc02_C10;OTUwell_3	Bacteria(100.0),Firmicutes(100.0),Bacilli(99.3),Bacillales(99.2),Bacillaceae_1(98.7),Bacillus(98.7)	k__Bacteria;p__Firmicutes;c__Bacilli;o__Bacillales;f__Bacillaceae	0,93
bc02_C10;OTUwell_4	Bacteria(100.0),Firmicutes(100.0),Bacilli(99.2),Bacillales(99.2),Bacillaceae_1(98.6),Bacillus(98.6)	k__Bacteria;p__Firmicutes;c__Bacilli;o__Bacillales;f__Bacillaceae;g__;s__	0,94
bc02_C11;OTUwell_1	Bacteria(100.0),Firmicutes(100.0),Bacilli(99.7),Bacillales(99.5),Bacillaceae_1(99.4),Bacillus(99.4)	k__Bacteria;p__Firmicutes;c__Bacilli;o__Bacillales;f__Bacillaceae;g__;s__	0,97
bc02_C11;OTUwell_2	Bacteria(100.0),"Bacteroidetes"(100.0),"Sphingobacteria"(98.2),"Sphingobacteriales"(98.2),Sphingobacteriaceae(98.2),Mucilaginibacter(95.9)	k__Bacteria;p__Bacteroidetes;c__Sphingobacteriia;o__Sphingobacteriales;f__Sphingobacteriaceae;g__;s__	1,00
bc02_C12;OTUwell_1	Bacteria(100.0),Firmicutes(100.0),Bacilli(99.7),Bacillales(99.6),Paenibacillaceae_1(99.5),Paenibacillus(99.4)	k__Bacteria;p__Firmicutes;c__Bacilli;o__Bacillales;f__Paenibacillaceae;g__Paenibacillus;s__	1,00
bc02_C12;OTUwell_2	Bacteria(100.0),Firmicutes(100.0),Bacilli(99.3),Bacillales(99.3),Paenibacillaceae_1(98.7),Paenibacillus(97.5)	k__Bacteria;p__Firmicutes;c__Bacilli;o__Bacillales;f__Paenibacillaceae;g__Paenibacillus	1,00
bc02_C12;OTUwell_3	Bacteria(100.0),Firmicutes(100.0),Bacilli(99.7),Bacillales(99.5),Bacillaceae_1(99.4),Bacillus(99.4)	k__Bacteria;p__Firmicutes;c__Bacilli;o__Bacillales;f__Bacillaceae;g__;s__	0,95
bc02_D01;OTUwell_1	Bacteria(100.0),Firmicutes(100.0),Bacilli(99.7),Bacillales(99.5),Bacillaceae_1(99.4),Bacillus(99.4)	k__Bacteria;p__Firmicutes;c__Bacilli;o__Bacillales;f__Bacillaceae;g__;s__	0,99
bc02_D01;OTUwell_2	Bacteria(100.0),Firmicutes(100.0),Bacilli(98.7),Bacillales(98.7),Bacillaceae_1(96.7),Bacillus(93.2)	k__Bacteria;p__Firmicutes;c__Bacilli;o__Bacillales;f__Bacillaceae;g__Bacillus;s__	0,92
bc02_D03;OTUwell_1	Bacteria(100.0),"Proteobacteria"(100.0),Alphaproteobacteria(99.0),Rhizobiales(99.0),Rhizobiaceae(99.0),Rhizobium(99.0)	k__Bacteria;p__Proteobacteria;c__Alphaproteobacteria;o__Rhizobiales;f__Rhizobiaceae;g__Rhizobium;s__	1,00
bc02_D03;OTUwell_2	Bacteria(100.0),Firmicutes(100.0),Bacilli(99.4),Bacillales(99.2),Bacillaceae_1(98.6),Bacillus(98.6)	k__Bacteria;p__Firmicutes;c__Bacilli;o__Bacillales;f__Bacillaceae;g__Bacillus	1,00
bc02_D03;OTUwell_3	Bacteria(100.0),"Actinobacteria"(100.0),Actinobacteria(100.0),Actinomycetales(99.6),Promicromonosporaceae(98.7),Promicromonospora(98.7)	k__Bacteria;p__Actinobacteria;c__Actinobacteria;o__Actinomycetales;f__Promicromonosporaceae;g__Promicromonospora;s__	1,00
bc02_D03;OTUwell_4	Bacteria(100.0),"Proteobacteria"(100.0),Alphaproteobacteria(97.2),Rhizobiales(97.2),Rhizobiaceae(97.2),Rhizobium(94.5)	k__Bacteria;p__Proteobacteria;c__Alphaproteobacteria;o__Rhizobiales;f__Rhizobiaceae;g__Rhizobium;s__	0,98
bc02_D03;OTUwell_5	Bacteria(100.0),Firmicutes(100.0),Bacilli(99.0),Bacillales(99.0),Bacillaceae_1(97.4),Bacillus(95.9)	k__Bacteria;p__Firmicutes;c__Bacilli;o__Bacillales;f__Bacillaceae;g__Bacillus	1,00
bc02_D03;OTUwell_6	Bacteria(100.0),Firmicutes(100.0),Bacilli(99.6),Bacillales(99.3),Bacillaceae_1(98.6),Bacillus(98.6)	k__Bacteria;p__Firmicutes;c__Bacilli;o__Bacillales;f__Bacillaceae;g__Bacillus	1,00
bc02_D03;OTUwell_7	Bacteria(100.0),"Bacteroidetes"(100.0),"Sphingobacteria"(99.6),"Sphingobacteriales"(99.6),Chitinophagaceae(99.6),Chitinophaga(99.2)	k__Bacteria;p__Bacteroidetes;c__[Saprospirae];o__[Saprospirales];f__Chitinophagaceae;g__Chitinophaga;s__	1,00
bc02_D04;OTUwell_1	Bacteria(100.0),"Proteobacteria"(100.0),Alphaproteobacteria(97.8),Rhizobiales(97.8),Rhizobiaceae(97.8),Rhizobium(97.1)	k__Bacteria;p__Proteobacteria;c__Alphaproteobacteria;o__Rhizobiales;f__Rhizobiaceae;g__Rhizobium;s__	1,00
bc02_D04;OTUwell_2	Bacteria(100.0),"Proteobacteria"(100.0),Gammaproteobacteria(99.6),Xanthomonadales(99.4),Xanthomonadaceae(99.4),Dyella(95.0)	k__Bacteria;p__Proteobacteria;c__Gammaproteobacteria;o__Xanthomonadales;f__Xanthomonadaceae;g__Dyella;s__	1,00
bc02_D04;OTUwell_3	Bacteria(100.0),"Bacteroidetes"(100.0),"Sphingobacteria"(98.9),"Sphingobacteriales"(98.9),Chitinophagaceae(98.9),Niastella(77.6)	k__Bacteria;p__Bacteroidetes;c__[Saprospirae];o__[Saprospirales];f__Chitinophagaceae;g__;s__	1,00
bc02_D04;OTUwell_4	Bacteria(100.0),"Proteobacteria"(100.0),Gammaproteobacteria(98.9),Xanthomonadales(98.9),Xanthomonadaceae(98.4),Dyella(82.2)	k__Bacteria;p__Proteobacteria;c__Gammaproteobacteria;o__Xanthomonadales;f__Xanthomonadaceae;g__Dyella;s__	1,00
bc02_D05;OTUwell_1	Bacteria(100.0),"Proteobacteria"(100.0),Betaproteobacteria(99.3),Burkholderiales(99.3),Burkholderiaceae(99.2),Wautersia(91.7)	k__Bacteria;p__Proteobacteria;c__Betaproteobacteria;o__Burkholderiales;f__Oxalobacteraceae;g__Cupriavidus;s__	1,00
bc02_D05;OTUwell_2	Bacteria(100.0),"Proteobacteria"(100.0),Gammaproteobacteria(99.4),Xanthomonadales(98.8),Xanthomonadaceae(98.2),Lysobacter(97.5)	k__Bacteria;p__Proteobacteria;c__Gammaproteobacteria;o__Xanthomonadales;f__Xanthomonadaceae;g__Lysobacter;s__	1,00
bc02_D06;OTUwell_1	Bacteria(100.0),"Proteobacteria"(100.0),Alphaproteobacteria(99.6),Caulobacterales(99.6),Caulobacteraceae(99.6),Caulobacter(99.2)	k__Bacteria;p__Proteobacteria;c__Alphaproteobacteria;o__Caulobacterales;f__Caulobacteraceae;g__Caulobacter;s__	1,00
bc02_D08;OTUwell_1	Bacteria(100.0),Firmicutes(100.0),Bacilli(99.3),Bacillales(99.3),Bacillaceae_1(98.7),Bacillus(98.7)	k__Bacteria;p__Firmicutes;c__Bacilli;o__Bacillales;f__Bacillaceae;g__;s__	0,96
bc02_D08;OTUwell_2	Bacteria(100.0),Firmicutes(100.0),Bacilli(99.3),Bacillales(99.2),Bacillaceae_1(98.7),Bacillus(98.7)	k__Bacteria;p__Firmicutes;c__Bacilli;o__Bacillales;f__Bacillaceae;g__;s__	0,81
bc02_D08;OTUwell_3	Bacteria(100.0),"Bacteroidetes"(100.0),"Sphingobacteria"(99.0),"Sphingobacteriales"(99.0),Chitinophagaceae(99.0),Chitinophaga(95.4)	k__Bacteria;p__Bacteroidetes;c__[Saprospirae];o__[Saprospirales];f__Chitinophagaceae;g__Chitinophaga;s__	1,00
bc02_D08;OTUwell_4	Bacteria(100.0),"Proteobacteria"(100.0),Betaproteobacteria(99.3),Burkholderiales(99.3),Comamonadaceae(98.3),Variovorax(96.6)	k__Bacteria;p__Proteobacteria;c__Betaproteobacteria;o__Burkholderiales;f__Comamonadaceae;g__Variovorax;s__paradoxus	0,85
bc02_D08;OTUwell_5	Bacteria(100.0),Firmicutes(100.0),Bacilli(99.4),Bacillales(99.4),Bacillaceae_1(99.0),Bacillus(99.0)	k__Bacteria;p__Firmicutes;c__Bacilli;o__Bacillales;f__Bacillaceae;g__;s__	0,93
bc02_D09;OTUwell_1	Bacteria(100.0),"Proteobacteria"(100.0),Gammaproteobacteria(99.6),Xanthomonadales(99.5),Xanthomonadaceae(99.4),Dyella(94.5)	k__Bacteria;p__Proteobacteria;c__Gammaproteobacteria;o__Xanthomonadales;f__Xanthomonadaceae;g__Dyella;s__	0,97
bc02_D10;OTUwell_1	Bacteria(100.0),Firmicutes(100.0),Bacilli(99.7),Bacillales(99.5),Bacillaceae_1(99.4),Bacillus(99.4)	k__Bacteria;p__Firmicutes;c__Bacilli;o__Bacillales;f__Bacillaceae;g__;s__	0,99
bc02_D10;OTUwell_2	Bacteria(100.0),"Bacteroidetes"(100.0),"Sphingobacteria"(98.2),"Sphingobacteriales"(98.2),Sphingobacteriaceae(98.2),Mucilaginibacter(96.3)	k__Bacteria;p__Bacteroidetes;c__Sphingobacteriia;o__Sphingobacteriales;f__Sphingobacteriaceae;g__;s__	1,00
bc02_D11;OTUwell_1	Bacteria(100.0),Firmicutes(100.0),Bacilli(99.7),Bacillales(99.5),Bacillaceae_1(99.5),Bacillus(99.5)	k__Bacteria;p__Firmicutes;c__Bacilli;o__Bacillales;f__Bacillaceae;g__;s__	1,00
bc02_D11;OTUwell_2	Bacteria(100.0),"Bacteroidetes"(100.0),"Sphingobacteria"(98.1),"Sphingobacteriales"(98.1),Sphingobacteriaceae(98.1),Mucilaginibacter(95.4)	k__Bacteria;p__Bacteroidetes;c__Sphingobacteriia;o__Sphingobacteriales;f__Sphingobacteriaceae;g__;s__	1,00
bc02_D11;OTUwell_3	Bacteria(100.0),Firmicutes(100.0),Bacilli(99.5),Bacillales(99.4),Bacillaceae_1(99.2),Bacillus(99.2)	k__Bacteria;p__Firmicutes;c__Bacilli;o__Bacillales;f__Bacillaceae;g__;s__	0,90
bc02_D12;OTUwell_1	Bacteria(100.0),Firmicutes(100.0),Bacilli(99.7),Bacillales(99.5),Bacillaceae_1(99.4),Bacillus(99.4)	k__Bacteria;p__Firmicutes;c__Bacilli;o__Bacillales;f__Bacillaceae;g__;s__	0,99
bc02_D12;OTUwell_2	Bacteria(100.0),"Proteobacteria"(100.0),Betaproteobacteria(98.2),Burkholderiales(98.2),Burkholderiaceae(97.7),Burkholderia(86.9)	k__Bacteria;p__Proteobacteria;c__Betaproteobacteria;o__Burkholderiales;f__Burkholderiaceae;g__Burkholderia	0,95
bc02_E01;OTUwell_1	Bacteria(100.0),"Bacteroidetes"(100.0),"Sphingobacteria"(99.8),"Sphingobacteriales"(99.6),Chitinophagaceae(99.6),Chitinophaga(99.6)	k__Bacteria;p__Bacteroidetes;c__[Saprospirae];o__[Saprospirales];f__Chitinophagaceae;g__Chitinophaga;s__	1,00
bc02_E01;OTUwell_2	Bacteria(100.0),"Bacteroidetes"(100.0),"Sphingobacteria"(99.9),"Sphingobacteriales"(99.9),Chitinophagaceae(99.9),Chitinophaga(99.9)	k__Bacteria;p__Bacteroidetes;c__[Saprospirae];o__[Saprospirales];f__Chitinophagaceae;g__Chitinophaga;s__	1,00
bc02_E02;OTUwell_1	Bacteria(100.0),"Bacteroidetes"(100.0),"Sphingobacteria"(98.2),"Sphingobacteriales"(98.2),Sphingobacteriaceae(98.2),Mucilaginibacter(95.9)	k__Bacteria;p__Bacteroidetes;c__Sphingobacteriia;o__Sphingobacteriales;f__Sphingobacteriaceae;g__;s__	1,00
bc02_E02;OTUwell_2	Bacteria(100.0),"Proteobacteria"(100.0),Gammaproteobacteria(99.5),Xanthomonadales(99.4),Xanthomonadaceae(99.3),Dyella(93.2)	k__Bacteria;p__Proteobacteria;c__Gammaproteobacteria;o__Xanthomonadales;f__Xanthomonadaceae;g__Dyella;s__	1,00
bc02_E03;OTUwell_1	Bacteria(100.0),Firmicutes(100.0),Bacilli(99.7),Bacillales(99.5),Bacillaceae_1(99.4),Bacillus(99.4)	k__Bacteria;p__Firmicutes;c__Bacilli;o__Bacillales;f__Bacillaceae;g__;s__	1,00
bc02_E03;OTUwell_2	Bacteria(100.0),"Proteobacteria"(100.0),Betaproteobacteria(99.0),Burkholderiales(99.0),Burkholderiaceae(98.7),Burkholderia(98.0)	k__Bacteria;p__Proteobacteria;c__Betaproteobacteria;o__Burkholderiales;f__Burkholderiaceae;g__Burkholderia;s__	0,88
bc02_E05;OTUwell_1	Bacteria(100.0),Firmicutes(100.0),Bacilli(99.7),Bacillales(99.5),Bacillaceae_1(99.4),Bacillus(99.4)	k__Bacteria;p__Firmicutes;c__Bacilli;o__Bacillales;f__Bacillaceae;g__;s__	1,00
bc02_E05;OTUwell_2	Bacteria(100.0),"Proteobacteria"(100.0),Alphaproteobacteria(97.2),Rhizobiales(97.2),Rhizobiaceae(96.7),Rhizobium(93.2)	k__Bacteria;p__Proteobacteria;c__Alphaproteobacteria;o__Rhizobiales;f__Rhizobiaceae;g__Rhizobium	0,99
bc02_E05;OTUwell_3	Bacteria(100.0),"Proteobacteria"(100.0),Alphaproteobacteria(98.6),Rhizobiales(98.6),Rhizobiaceae(98.6),Rhizobium(98.6)	k__Bacteria;p__Proteobacteria;c__Alphaproteobacteria;o__Rhizobiales;f__Rhizobiaceae;g__Rhizobium;s__	0,93
bc02_E05;OTUwell_4	Bacteria(100.0),"Bacteroidetes"(100.0),"Sphingobacteria"(99.3),"Sphingobacteriales"(99.3),Sphingobacteriaceae(99.3),Mucilaginibacter(99.3)	k__Bacteria;p__Bacteroidetes;c__Sphingobacteriia;o__Sphingobacteriales;f__Sphingobacteriaceae;g__;s__	1,00
bc02_E06;OTUwell_1	Bacteria(100.0),"Proteobacteria"(100.0),Betaproteobacteria(99.1),Burkholderiales(99.1),Burkholderiaceae(98.8),Burkholderia(97.7)	k__Bacteria;p__Proteobacteria;c__Betaproteobacteria;o__Burkholderiales;f__Burkholderiaceae;g__Burkholderia;s__	1,00
bc02_E07;OTUwell_1	Bacteria(100.0),"Proteobacteria"(100.0),Betaproteobacteria(99.4),Burkholderiales(99.4),Burkholderiaceae(99.4),Burkholderia(99.3)	k__Bacteria;p__Proteobacteria;c__Betaproteobacteria;o__Burkholderiales;f__Burkholderiaceae;g__Burkholderia;s__	1,00
bc02_E07;OTUwell_2	Bacteria(100.0),"Proteobacteria"(100.0),Betaproteobacteria(98.7),Burkholderiales(98.7),Comamonadaceae(97.5),Variovorax(94.5)	k__Bacteria;p__Proteobacteria;c__Betaproteobacteria;o__Burkholderiales;f__Comamonadaceae;g__Variovorax;s__paradoxus	0,91
bc02_E09;OTUwell_1	Bacteria(100.0),"Proteobacteria"(100.0),Betaproteobacteria(99.0),Burkholderiales(99.0),Burkholderiaceae(98.7),Burkholderia(97.7)	k__Bacteria;p__Proteobacteria;c__Betaproteobacteria;o__Burkholderiales;f__Burkholderiaceae;g__Burkholderia;s__	0,83
bc02_E10;OTUwell_1	Bacteria(100.0),Firmicutes(100.0),Bacilli(99.7),Bacillales(99.5),Bacillaceae_1(99.4),Bacillus(99.4)	k__Bacteria;p__Firmicutes;c__Bacilli;o__Bacillales;f__Bacillaceae;g__;s__	0,98
bc02_E11;OTUwell_1	Bacteria(100.0),"Proteobacteria"(100.0),Gammaproteobacteria(99.5),Xanthomonadales(99.3),Xanthomonadaceae(99.3),Fulvimonas(90.8)	k__Bacteria;p__Proteobacteria;c__Gammaproteobacteria;o__Xanthomonadales;f__Xanthomonadaceae;g__Dyella;s__	0,95
bc02_F01;OTUwell_1	Bacteria(100.0),Firmicutes(100.0),Bacilli(99.7),Bacillales(99.5),Bacillaceae_1(99.4),Bacillus(99.4)	k__Bacteria;p__Firmicutes;c__Bacilli;o__Bacillales;f__Bacillaceae;g__;s__	1,00
bc02_F01;OTUwell_2	Bacteria(100.0),"Proteobacteria"(100.0),Betaproteobacteria(99.5),Burkholderiales(99.4),Burkholderiaceae(99.4),Burkholderia(99.2)	k__Bacteria;p__Proteobacteria;c__Betaproteobacteria;o__Burkholderiales;f__Burkholderiaceae;g__Burkholderia;s__	1,00
bc02_F02;OTUwell_1	Bacteria(100.0),"Proteobacteria"(100.0),Betaproteobacteria(99.2),Burkholderiales(99.2),Comamonadaceae(98.2),Variovorax(96.6)	k__Bacteria;p__Proteobacteria;c__Betaproteobacteria;o__Burkholderiales;f__Comamonadaceae;g__Variovorax;s__paradoxus	0,94
bc02_F05;OTUwell_1	Bacteria(100.0),Firmicutes(100.0),Bacilli(99.7),Bacillales(99.5),Bacillaceae_1(99.4),Bacillus(99.4)	k__Bacteria;p__Firmicutes;c__Bacilli;o__Bacillales;f__Bacillaceae;g__;s__	0,97
bc02_F05;OTUwell_2	Bacteria(100.0),"Proteobacteria"(100.0),Gammaproteobacteria(99.5),Xanthomonadales(99.3),Xanthomonadaceae(99.2),Dyella(93.2)	k__Bacteria;p__Proteobacteria;c__Gammaproteobacteria;o__Xanthomonadales;f__Xanthomonadaceae;g__Dyella;s__	1,00
bc02_F05;OTUwell_3	Bacteria(100.0),"Bacteroidetes"(100.0),"Sphingobacteria"(99.9),"Sphingobacteriales"(99.9),Chitinophagaceae(99.9),Chitinophaga(99.9)	k__Bacteria;p__Bacteroidetes;c__[Saprospirae];o__[Saprospirales];f__Chitinophagaceae;g__Chitinophaga;s__	1,00
bc02_F05;OTUwell_4	Bacteria(100.0),"Proteobacteria"(100.0),Gammaproteobacteria(99.4),Xanthomonadales(99.3),Xanthomonadaceae(99.2),Dyella(90.8)	k__Bacteria;p__Proteobacteria;c__Gammaproteobacteria;o__Xanthomonadales;f__Xanthomonadaceae;g__Dyella;s__	1,00
bc02_F06;OTUwell_1	Bacteria(100.0),"Actinobacteria"(100.0),Actinobacteria(100.0),Actinomycetales(100.0),Microbacteriaceae(99.4),Microbacterium(99.0)	k__Bacteria;p__Actinobacteria;c__Actinobacteria;o__Actinomycetales;f__Microbacteriaceae;g__Microbacterium;s__	0,94
bc02_F06;OTUwell_2	Bacteria(100.0),"Proteobacteria"(100.0),Gammaproteobacteria(99.6),Xanthomonadales(99.5),Xanthomonadaceae(99.5),Dyella(95.0)	k__Bacteria;p__Proteobacteria;c__Gammaproteobacteria;o__Xanthomonadales;f__Xanthomonadaceae;g__Dyella;s__	1,00
bc02_F10;OTUwell_1	Bacteria(100.0),Firmicutes(100.0),Bacilli(99.7),Bacillales(99.5),Bacillaceae_1(99.5),Bacillus(99.5)	k__Bacteria;p__Firmicutes;c__Bacilli;o__Bacillales;f__Bacillaceae;g__;s__	1,00
bc02_F12;OTUwell_1	Bacteria(100.0),"Proteobacteria"(100.0),Gammaproteobacteria(99.8),Xanthomonadales(99.2),Xanthomonadaceae(99.0),Lysobacter(99.0)	k__Bacteria;p__Proteobacteria;c__Gammaproteobacteria;o__Xanthomonadales;f__Xanthomonadaceae;g__Lysobacter;s__	1,00
bc02_F12;OTUwell_2	Bacteria(100.0),Firmicutes(100.0),Bacilli(99.7),Bacillales(99.5),Bacillaceae_1(99.4),Bacillus(99.4)	k__Bacteria;p__Firmicutes;c__Bacilli;o__Bacillales;f__Bacillaceae;g__;s__	1,00
bc02_F12;OTUwell_3	Bacteria(100.0),"Proteobacteria"(100.0),Gammaproteobacteria(99.3),Xanthomonadales(99.3),Xanthomonadaceae(99.1),Dyella(90.8)	k__Bacteria;p__Proteobacteria;c__Gammaproteobacteria;o__Xanthomonadales;f__Xanthomonadaceae;g__Dyella;s__	1,00

bc02_F12;OTUwell_4	Bacteria(100.0),"Proteobacteria"(100.0),Gammaproteobacteria(99.5),Xanthomonadales(99.3),Xanthomonadaceae(99.2),Dyella(92.4)	k__Bacteria;p__Proteobacteria;c__Gammaproteobacteria;o__Xanthomonadales;f__Xanthomonadaceae;g__Dyella;s__	1,00
bc02_G01;OTUwell_1	Bacteria(100.0),"Proteobacteria"(100.0),Betaproteobacteria(99.3),Burkholderiales(99.3),Burkholderiaceae(99.3),Burkholderia(99.0)	k__Bacteria;p__Proteobacteria;c__Betaproteobacteria;o__Burkholderiales;f__Burkholderiaceae;g__Burkholderia;s__	0,99
bc02_G01;OTUwell_2	Bacteria(100.0),Firmicutes(100.0),Bacilli(99.6),Bacillales(99.4),Bacillaceae_1(99.2),Bacillus(99.2)	k__Bacteria;p__Firmicutes;c__Bacilli;o__Bacillales;f__Bacillaceae;g__;s__	0,95
bc02_G01;OTUwell_3	Bacteria(100.0),Firmicutes(100.0),Bacilli(99.5),Bacillales(99.4),Bacillaceae_1(99.1),Bacillus(99.1)	k__Bacteria;p__Firmicutes;c__Bacilli;o__Bacillales;f__Bacillaceae;g__;s__	0,85
bc02_G03;OTUwell_1	Bacteria(100.0),"Bacteroidetes"(100.0),"Sphingobacteria"(99.9),"Sphingobacteriales"(99.9),Chitinophagaceae(99.9),Chitinophaga(99.9)	k__Bacteria;p__Bacteroidetes;c__[Saprospirae];o__[Saprospirales];f__Chitinophagaceae;g__Chitinophaga;s__	1,00
bc02_G11;OTUwell_1	Bacteria(100.0),"Bacteroidetes"(100.0),"Sphingobacteria"(98.1),"Sphingobacteriales"(98.1),Sphingobacteriaceae(98.1),Mucilaginibacter(95.4)	k__Bacteria;p__Bacteroidetes;c__Sphingobacteriia;o__Sphingobacteriales;f__Sphingobacteriaceae;g__;s__	1,00
bc02_G12;OTUwell_1	Bacteria(100.0),Firmicutes(100.0),Bacilli(99.7),Bacillales(99.5),Bacillaceae_1(99.4),Bacillus(99.4)	k__Bacteria;p__Firmicutes;c__Bacilli;o__Bacillales;f__Bacillaceae;g__;s__	0,98
bc02_G12;OTUwell_2	Bacteria(100.0),"Bacteroidetes"(100.0),"Sphingobacteria"(99.9),"Sphingobacteriales"(99.9),Chitinophagaceae(99.9),Chitinophaga(99.9)	k__Bacteria;p__Bacteroidetes;c__[Saprospirae];o__[Saprospirales];f__Chitinophagaceae;g__Chitinophaga;s__	1,00
bc02_G12;OTUwell_3	Bacteria(100.0),Firmicutes(100.0),Bacilli(99.0),Bacillales(99.0),Bacillaceae_1(98.6),Bacillus(98.6)	k__Bacteria;p__Firmicutes;c__Bacilli;o__Bacillales;f__Bacillaceae;g__;s__	0,99
bc02_G12;OTUwell_4	Bacteria(100.0),"Proteobacteria"(100.0),Alphaproteobacteria(98.6),Rhizobiales(98.6),Rhizobiaceae(98.6),Rhizobium(98.6)	k__Bacteria;p__Proteobacteria;c__Alphaproteobacteria;o__Rhizobiales;f__Rhizobiaceae;g__Rhizobium;s__	1,00
bc02_G12;OTUwell_5	Bacteria(100.0),"Proteobacteria"(100.0),Gammaproteobacteria(99.5),Xanthomonadales(99.3),Xanthomonadaceae(99.2),Dyella(90.8)	k__Bacteria;p__Proteobacteria;c__Gammaproteobacteria;o__Xanthomonadales;f__Xanthomonadaceae;g__Dyella;s__	1,00
bc02_H02;OTUwell_1	Bacteria(100.0),"Acidobacteria"(84.2),Holophagae(62.5),Holophagales(53.7),Holophagaceae(42.6),Holophaga(42.6)	k__Bacteria;p__Acidobacteria;c__Acidobacteriia;o__Acidobacteriales;f__Acidobacteriaceae;g__Terriglobus;s__	1,00
bc02_H03;OTUwell_1	Bacteria(100.0),"Proteobacteria"(100.0),Gammaproteobacteria(100.0),"Enterobacteriales"(99.1),Enterobacteriaceae(98.8),Enterobacter(95.4)	k__Bacteria;p__Proteobacteria;c__Gammaproteobacteria;o__Enterobacteriales;f__Enterobacteriaceae;g__;s__	0,83
bc02_H04;OTUwell_1	Bacteria(100.0),"Actinobacteria"(100.0),Actinobacteria(99.6),Actinomycetales(99.6),Micrococcaceae(98.6),Arthrobacter(95.4)	k__Bacteria;p__Actinobacteria;c__Actinobacteria;o__Actinomycetales;f__Micrococcaceae;g__;s__	0,99
bc02_H04;OTUwell_2	Bacteria(100.0),"Proteobacteria"(100.0),Gammaproteobacteria(100.0),"Enterobacteriales"(99.1),Enterobacteriaceae(98.6),Enterobacter(93.8)	k__Bacteria;p__Proteobacteria;c__Gammaproteobacteria;o__Enterobacteriales;f__Enterobacteriaceae;g__;s__	0,97
bc02_H04;OTUwell_3	Bacteria(100.0),"Proteobacteria"(100.0),Gammaproteobacteria(100.0),"Enterobacteriales"(98.4),Enterobacteriaceae(97.4),Enterobacter(84.7)	k__Bacteria;p__Proteobacteria;c__Gammaproteobacteria;o__Enterobacteriales;f__Enterobacteriaceae;g__;s__	0,92
bc02_H06;OTUwell_1	Bacteria(100.0),"Proteobacteria"(100.0),Betaproteobacteria(98.2),Burkholderiales(98.2),Burkholderiaceae(97.2),Burkholderia(82.2)	k__Bacteria;p__Proteobacteria;c__Betaproteobacteria;o__Burkholderiales;f__Burkholderiaceae;g__Burkholderia;s__	1,00
bc02_H07;OTUwell_1	Bacteria(100.0),"Proteobacteria"(100.0),Betaproteobacteria(98.7),Burkholderiales(98.7),Burkholderiaceae(98.5),Burkholderia(96.6)	k__Bacteria;p__Proteobacteria;c__Betaproteobacteria;o__Burkholderiales;f__Burkholderiaceae	1,00
bc02_H07;OTUwell_2	Bacteria(100.0),Firmicutes(100.0),Bacilli(99.6),Bacillales(99.4),Planococcaceae(98.7),Sporosarcina(98.0)	k__Bacteria;p__Firmicutes;c__Bacilli;o__Bacillales;f__Planococcaceae;g__Sporosarcina;s__	1,00
bc02_H07;OTUwell_3	Bacteria(100.0),"Proteobacteria"(100.0),Alphaproteobacteria(99.6),Rhizobiales(99.5),Bradyrhizobiaceae(99.4),Bosea(99.4)	k__Bacteria;p__Proteobacteria;c__Alphaproteobacteria;o__Rhizobiales;f__Beijerinckiaceae;g__;s__	0,86
bc02_H08;OTUwell_1	Bacteria(100.0),Firmicutes(100.0),Bacilli(99.7),Bacillales(99.5),Bacillaceae_1(99.4),Bacillus(99.4)	k__Bacteria;p__Firmicutes;c__Bacilli;o__Bacillales;f__Bacillaceae;g__;s__	0,99
bc02_H08;OTUwell_2	Bacteria(100.0),Firmicutes(100.0),Bacilli(99.2),Bacillales(99.2),Bacillaceae_1(98.6),Bacillus(98.6)	k__Bacteria;p__Firmicutes;c__Bacilli;o__Bacillales;f__Bacillaceae;g__;s__	0,87
bc02_H10;OTUwell_1	Bacteria(100.0),"Proteobacteria"(100.0),Gammaproteobacteria(100.0),"Enterobacteriales"(99.4),Enterobacteriaceae(99.4),Escherichia/Shigella(99.0)	k__Bacteria;p__Proteobacteria;c__Gammaproteobacteria;o__Enterobacteriales;f__Enterobacteriaceae;g__;s__	1,00
bc02_H11;OTUwell_1	Bacteria(100.0),"Proteobacteria"(100.0),Gammaproteobacteria(100.0),"Enterobacteriales"(99.4),Enterobacteriaceae(99.4),Escherichia/Shigella(98.8)	k__Bacteria;p__Proteobacteria;c__Gammaproteobacteria;o__Enterobacteriales;f__Enterobacteriaceae;g__;s__	1,00
bc02_H11;OTUwell_2	Bacteria(100.0),"Proteobacteria"(100.0),Gammaproteobacteria(99.2),"Enterobacteriales"(98.3),Enterobacteriaceae(97.4),Escherichia/Shigella(85.8)	k__Bacteria;p__Proteobacteria;c__Gammaproteobacteria;o__Enterobacteriales;f__Enterobacteriaceae;g__;s__	1,00
bc02_H11;OTUwell_3	Bacteria(100.0),"Proteobacteria"(100.0),Gammaproteobacteria(100.0),"Enterobacteriales"(99.0),Enterobacteriaceae(98.5),Escherichia/Shigella(93.8)	k__Bacteria;p__Proteobacteria;c__Gammaproteobacteria;o__Enterobacteriales;f__Enterobacteriaceae;g__;s__	0,98
bc02_H12;OTUwell_1	Bacteria(100.0),"Proteobacteria"(100.0),Gammaproteobacteria(100.0),"Enterobacteriales"(99.4),Enterobacteriaceae(99.4),Escherichia/Shigella(98.9)	k__Bacteria;p__Proteobacteria;c__Gammaproteobacteria;o__Enterobacteriales;f__Enterobacteriaceae;g__;s__	0,99
bc02_H12;OTUwell_2	Bacteria(100.0),"Proteobacteria"(100.0),Gammaproteobacteria(99.7),"Enterobacteriales"(99.1),Enterobacteriaceae(98.7),Escherichia/Shigella(94.5)	k__Bacteria;p__Proteobacteria;c__Gammaproteobacteria;o__Enterobacteriales;f__Enterobacteriaceae;g__;s__	1,00
bc03_A01;OTUwell_1	Bacteria(100.0),"Bacteroidetes"(100.0),"Sphingobacteria"(99.6),"Sphingobacteriales"(99.6),Chitinophagaceae(99.6),Chitinophaga(99.0)	k__Bacteria;p__Bacteroidetes;c__[Saprospirae];o__[Saprospirales];f__Chitinophagaceae;g__Chitinophaga;s__	1,00
bc03_A03;OTUwell_1	Bacteria(100.0),"Proteobacteria"(100.0),Gammaproteobacteria(100.0),"Enterobacteriales"(99.2),Enterobacteriaceae(98.9),Enterobacter(95.9)	k__Bacteria;p__Proteobacteria;c__Gammaproteobacteria;o__Enterobacteriales;f__Enterobacteriaceae;g__;s__	0,94
bc03_A04;OTUwell_1	Bacteria(100.0),"Proteobacteria"(100.0),Betaproteobacteria(99.1),Burkholderiales(99.1),Comamonadaceae(98.2),Variovorax(96.3)	k__Bacteria;p__Proteobacteria;c__Betaproteobacteria;o__Burkholderiales;f__Comamonadaceae;g__Variovorax;s__paradoxus	0,90
bc03_A05;OTUwell_1	Bacteria(100.0),"Actinobacteria"(100.0),Actinobacteria(100.0),Actinomycetales(99.6),Micrococcaceae(99.0),Arthrobacter(98.7)	k__Bacteria;p__Actinobacteria;c__Actinobacteria;o__Actinomycetales;f__Micrococcaceae;g__;s__	1,00
bc03_A06;OTUwell_1	Bacteria(100.0),"Proteobacteria"(100.0),Betaproteobacteria(99.3),Burkholderiales(99.3),Comamonadaceae(98.2),Variovorax(96.6)	k__Bacteria;p__Proteobacteria;c__Betaproteobacteria;o__Burkholderiales;f__Comamonadaceae;g__Variovorax;s__paradoxus	0,90
bc03_A07;OTUwell_1	Bacteria(100.0),"Proteobacteria"(100.0),Gammaproteobacteria(99.5),Xanthomonadales(99.3),Xanthomonadaceae(99.3),Fulvimonas(90.8)	k__Bacteria;p__Proteobacteria;c__Gammaproteobacteria;o__Xanthomonadales;f__Xanthomonadaceae;g__Dyella;s__	0,94
bc03_A07;OTUwell_2	Bacteria(100.0),"Proteobacteria"(100.0),Gammaproteobacteria(100.0),"Enterobacteriales"(99.1),Enterobacteriaceae(98.7),Enterobacter(95.0)	k__Bacteria;p__Proteobacteria;c__Gammaproteobacteria;o__Enterobacteriales;f__Enterobacteriaceae;g__;s__	0,87
bc03_A09;OTUwell_1	Bacteria(100.0),"Bacteroidetes"(100.0),"Sphingobacteria"(98.1),"Sphingobacteriales"(98.1),Sphingobacteriaceae(98.1),Mucilaginibacter(95.9)	k__Bacteria;p__Bacteroidetes;c__Sphingobacteriia;o__Sphingobacteriales;f__Sphingobacteriaceae;g__;s__	1,00
bc03_A11;OTUwell_1	Bacteria(100.0),"Proteobacteria"(100.0),Betaproteobacteria(97.4),Burkholderiales(97.4),Oxalobacteraceae(97.4),Herbaspirillum(91.7)	k__Bacteria;p__Proteobacteria;c__Betaproteobacteria;o__Burkholderiales;f__Oxalobacteraceae;g__;s__	0,96
bc03_A11;OTUwell_2	Bacteria(100.0),"Proteobacteria"(100.0),Betaproteobacteria(96.5),Burkholderiales(96.5),Oxalobacteraceae(95.9),Herbaspirillum(83.4)	k__Bacteria;p__Proteobacteria;c__Betaproteobacteria;o__Burkholderiales;f__Oxalobacteraceae	0,99
bc03_A12;OTUwell_1	Bacteria(100.0),"Bacteroidetes"(100.0),"Sphingobacteria"(99.4),"Sphingobacteriales"(99.4),Sphingobacteriaceae(99.4),Mucilaginibacter(99.4)	k__Bacteria;p__Bacteroidetes;c__Sphingobacteriia;o__Sphingobacteriales;f__Sphingobacteriaceae;g__;s__	1,00
bc03_A12;OTUwell_2	Bacteria(100.0),"Bacteroidetes"(100.0),"Sphingobacteria"(99.4),"Sphingobacteriales"(99.4),Cytophagaceae(99.4),Dyadobacter(99.4)	k__Bacteria;p__Bacteroidetes;c__Cytophagia;o__Cytophagales;f__Cytophagaceae;g__Dyadobacter;s__	1,00
bc03_B02;OTUwell_1	Bacteria(100.0),"Proteobacteria"(100.0),Alphaproteobacteria(98.9),Rhizobiales(98.9),Rhizobiaceae(98.9),Rhizobium(98.9)	k__Bacteria;p__Proteobacteria;c__Alphaproteobacteria;o__Rhizobiales;f__Rhizobiaceae;g__Rhizobium;s__	1,00
bc03_B03;OTUwell_1	Bacteria(100.0),"Bacteroidetes"(100.0),"Sphingobacteria"(98.2),"Sphingobacteriales"(98.2),Sphingobacteriaceae(98.2),Mucilaginibacter(96.3)	k__Bacteria;p__Bacteroidetes;c__Sphingobacteriia;o__Sphingobacteriales;f__Sphingobacteriaceae;g__;s__	1,00
bc03_B04;OTUwell_1	Bacteria(100.0),"Proteobacteria"(100.0),Betaproteobacteria(98.9),Burkholderiales(98.9),Comamonadaceae(97.8),Variovorax(96.3)	k__Bacteria;p__Proteobacteria;c__Betaproteobacteria;o__Burkholderiales;f__Comamonadaceae;g__Variovorax;s__paradoxus	0,93
bc03_B05;OTUwell_1	Bacteria(100.0),"Proteobacteria"(100.0),Betaproteobacteria(99.6),Burkholderiales(99.5),Burkholderiaceae(99.5),Burkholderia(99.5)	k__Bacteria;p__Proteobacteria;c__Betaproteobacteria;o__Burkholderiales;f__Burkholderiaceae;g__Burkholderia;s__	1,00
bc03_B05;OTUwell_2	Bacteria(100.0),"Proteobacteria"(100.0),Alphaproteobacteria(98.9),Rhodospirillales(98.9),Rhodospirillaceae(98.8),Inquilinus(98.8)	k__Bacteria;p__Proteobacteria;c__Alphaproteobacteria;o__Rhodospirillales;f__Rhodospirillaceae;g__Inquilinus;s__limosus	1,00
bc03_B06;OTUwell_1	Bacteria(100.0),"Proteobacteria"(100.0),Betaproteobacteria(99.2),Burkholderiales(99.2),Comamonadaceae(97.8),Variovorax(95.9)	k__Bacteria;p__Proteobacteria;c__Betaproteobacteria;o__Burkholderiales;f__Comamonadaceae;g__Variovorax;s__paradoxus	0,82
bc03_B07;OTUwell_1	Bacteria(100.0),"Bacteroidetes"(100.0),"Sphingobacteria"(98.0),"Sphingobacteriales"(98.0),Sphingobacteriaceae(98.0),Mucilaginibacter(95.0)	k__Bacteria;p__Bacteroidetes;c__Sphingobacteriia;o__Sphingobacteriales;f__Sphingobacteriaceae;g__;s__	1,00
bc03_B07;OTUwell_2	Bacteria(100.0),"Bacteroidetes"(100.0),"Sphingobacteria"(96.4),"Sphingobacteriales"(96.4),Sphingobacteriaceae(95.9),Mucilaginibacter(82.2)	k__Bacteria;p__Bacteroidetes;c__Sphingobacteriia;o__Sphingobacteriales;f__Sphingobacteriaceae;g__;s__	1,00
bc03_B07;OTUwell_3	Bacteria(100.0),"Bacteroidetes"(100.0),"Sphingobacteria"(96.6),"Sphingobacteriales"(96.6),Sphingobacteriaceae(96.4),Mucilaginibacter(84.7)	k__Bacteria;p__Bacteroidetes;c__Sphingobacteriia;o__Sphingobacteriales;f__Sphingobacteriaceae;g__;s__	1,00
bc03_B10;OTUwell_1	Bacteria(100.0),"Proteobacteria"(100.0),Alphaproteobacteria(99.5),Caulobacterales(99.5),Caulobacteraceae(99.5),Caulobacter(98.0)	k__Bacteria;p__Proteobacteria;c__Alphaproteobacteria;o__Caulobacterales;f__Caulobacteraceae;g__Caulobacter;s__	0,98
bc03_B12;OTUwell_1	Bacteria(100.0),"Proteobacteria"(100.0),Alphaproteobacteria(98.5),Rhizobiales(98.5),Rhizobiaceae(98.5),Ensifer(97.5)	k__Bacteria;p__Proteobacteria;c__Alphaproteobacteria;o__Rhizobiales;f__Rhizobiaceae;g__;s__	0,96
bc03_B12;OTUwell_2	Bacteria(100.0),Firmicutes(100.0),Bacilli(99.6),Bacillales(99.5),Bacillaceae_1(99.1),Bacillus(99.1)	k__Bacteria;p__Firmicutes;c__Bacilli;o__Bacillales;f__Bacillaceae;g__Bacillus	1,00
bc03_B12;OTUwell_3	Bacteria(100.0),"Proteobacteria"(100.0),Betaproteobacteria(99.0),Burkholderiales(99.0),Burkholderiaceae(99.0),Burkholderia(98.3)	k__Bacteria;p__Proteobacteria;c__Betaproteobacteria;o__Burkholderiales;f__Burkholderiaceae;g__Burkholderia;s__	1,00
bc03_C01;OTUwell_1	Bacteria(100.0),"Proteobacteria"(100.0),Betaproteobacteria(99.3),Burkholderiales(99.3),Burkholderiaceae(99.3),Wautersia(94.5)	k__Bacteria;p__Proteobacteria;c__Betaproteobacteria;o__Burkholderiales;f__Oxalobacteraceae;g__Cupriavidus;s__	1,00
bc03_C01;OTUwell_2	Bacteria(100.0),Firmicutes(100.0),Bacilli(99.6),Bacillales(99.5),Bacillaceae_1(99.4),Bacillus(99.4)	k__Bacteria;p__Firmicutes;c__Bacilli;o__Bacillales;f__Bacillaceae;g__;s__	0,93
bc03_C01;OTUwell_3	Bacteria(100.0),Firmicutes(100.0),Bacilli(99.5),Bacillales(99.5),Bacillaceae_1(99.3),Bacillus(99.3)	k__Bacteria;p__Firmicutes;c__Bacilli;o__Bacillales;f__Bacillaceae;g__;s__	0,91
bc03_C01;OTUwell_4	Bacteria(100.0),Firmicutes(100.0),Bacilli(99.6),Bacillales(99.4),Bacillaceae_1(99.0),Bacillus(99.0)	k__Bacteria;p__Firmicutes;c__Bacilli;o__Bacillales;f__Bacillaceae;g__Bacillus	1,00
bc03_C01;OTUwell_5	Bacteria(100.0),"Proteobacteria"(100.0),Betaproteobacteria(98.9),Burkholderiales(98.9),Burkholderiaceae(98.7),Wautersia(86.9)	k__Bacteria;p__Proteobacteria;c__Betaproteobacteria;o__Burkholderiales;f__Oxalobacteraceae;g__Cupriavidus;s__	1,00
bc03_C03;OTUwell_1	Bacteria(100.0),"Proteobacteria"(100.0),Gammaproteobacteria(99.4),Xanthomonadales(99.3),Xanthomonadaceae(99.2),Dyella(90.8)	k__Bacteria;p__Proteobacteria;c__Gammaproteobacteria;o__Xanthomonadales;f__Xanthomonadaceae;g__Dyella;s__	1,00
bc03_C03;OTUwell_2	Bacteria(100.0),"Proteobacteria"(100.0),Gammaproteobacteria(99.6),Xanthomonadales(99.5),Xanthomonadaceae(99.4),Dyella(95.0)	k__Bacteria;p__Proteobacteria;c__Gammaproteobacteria;o__Xanthomonadales;f__Xanthomonadaceae;g__Dyella;s__	1,00
bc03_C04;OTUwell_1	Bacteria(100.0),"Proteobacteria"(100.0),Alphaproteobacteria(98.8),Rhizobiales(98.8),Rhizobiaceae(98.8),Rhizobium(98.8)	k__Bacteria;p__Proteobacteria;c__Alphaproteobacteria;o__Rhizobiales;f__Rhizobiaceae;g__Rhizobium;s__	1,00
bc03_C09;OTUwell_1	Bacteria(100.0),"Proteobacteria"(100.0),Alphaproteobacteria(98.1),Sphingomonadales(94.2),Sphingomonadaceae(92.9),Sphingomonas(81.1)	k__Bacteria;p__Proteobacteria;c__Alphaproteobacteria;o__Sphingomonadales;f__Sphingomonadaceae;g__Sphingomonas;s__wittichii	1,00
bc03_C09;OTUwell_2	Bacteria(100.0),"Proteobacteria"(100.0),Alphaproteobacteria(97.3),Sphingomonadales(91.4),Sphingomonadaceae(89.4),Sphingomonas(70.9)	k__Bacteria;p__Proteobacteria;c__Alphaproteobacteria;o__Sphingomonadales;f__Sphingomonadaceae;g__Sphingomonas;s__wittichii	0,93
bc03_D01;OTUwell_1	Bacteria(100.0),"Proteobacteria"(100.0),Betaproteobacteria(99.3),Burkholderiales(99.3),Burkholderiaceae(99.2),Wautersia(91.7)	k__Bacteria;p__Proteobacteria;c__Betaproteobacteria;o__Burkholderiales;f__Oxalobacteraceae;g__Cupriavidus;s__	1,00
bc03_D01;OTUwell_2	Bacteria(100.0),"Bacteroidetes"(100.0),"Sphingobacteria"(99.8),"Sphingobacteriales"(99.6),Chitinophagaceae(99.6),Chitinophaga(99.6)	k__Bacteria;p__Bacteroidetes;c__[Saprospirae];o__[Saprospirales];f__Chitinophagaceae;g__Chitinophaga;s__	1,00
bc03_D02;OTUwell_1	Bacteria(100.0),"Proteobacteria"(100.0),Betaproteobacteria(99.3),Burkholderiales(99.3),Burkholderiaceae(99.3),Wautersia(92.4)	k__Bacteria;p__Proteobacteria;c__Betaproteobacteria;o__Burkholderiales;f__Oxalobacteraceae;g__Cupriavidus;s__	1,00
bc03_D02;OTUwell_2	Bacteria(100.0),"Proteobacteria"(100.0),Alphaproteobacteria(98.5),Rhizobiales(98.5),Rhizobiaceae(98.5),Rhizobium(98.5)	k__Bacteria;p__Proteobacteria;c__Alphaproteobacteria;o__Rhizobiales;f__Rhizobiaceae;g__Rhizobium;s__	0,96
bc03_D02;OTUwell_3	Bacteria(100.0),Firmicutes(100.0),Bacilli(99.5),Bacillales(99.3),Bacillaceae_1(98.6),Bacillus(98.6)	k__Bacteria;p__Firmicutes;c__Bacilli;o__Bacillales;f__Bacillaceae;g__Bacillus	1,00
bc03_D02;OTUwell_4	Bacteria(100.0),"Proteobacteria"(100.0),Gammaproteobacteria(100.0),"Enterobacteriales"(99.1),Enterobacteriaceae(98.7),Enterobacter(95.0)	k__Bacteria;p__Proteobacteria;c__Gammaproteobacteria;o__Enterobacteriales;f__Enterobacteriaceae;g__;s__	0,83
bc03_D03;OTUwell_1	Bacteria(100.0),"Proteobacteria"(100.0),Alphaproteobacteria(98.6),Rhizobiales(98.6),Rhizobiaceae(98.6),Rhizobium(98.6)	k__Bacteria;p__Proteobacteria;c__Alphaproteobacteria;o__Rhizobiales;f__Rhizobiaceae;g__Rhizobium;s__	1,00
bc03_D03;OTUwell_2	Bacteria(100.0),Firmicutes(100.0),Bacilli(99.6),Bacillales(99.5),Bacillaceae_1(99.1),Bacillus(99.1)	k__Bacteria;p__Firmicutes;c__Bacilli;o__Bacillales;f__Bacillaceae;g__Bacillus	1,00
bc03_D04;OTUwell_1	Bacteria(100.0),"Proteobacteria"(100.0),Gammaproteobacteria(98.7),Xanthomonadales(98.7),Xanthomonadaceae(98.3),Fulvimonas(77.6)	k__Bacteria;p__Proteobacteria;c__Gammaproteobacteria;o__Xanthomonadales;f__Xanthomonadaceae;g__Dyella;s__	0,98
bc03_D04;OTUwell_2	Bacteria(100.0),"Proteobacteria"(100.0),Gammaproteobacteria(99.7),Xanthomonadales(99.5),Xanthomonadaceae(99.5),Dyella(96.6)	k__Bacteria;p__Proteobacteria;c__Gammaproteobacteria;o__Xanthomonadales;f__Xanthomonadaceae;g__Dyella;s__	1,00
bc03_D06;OTUwell_1	Bacteria(100.0),"Proteobacteria"(100.0),Alphaproteobacteria(99.6),Caulobacterales(99.6),Caulobacteraceae(99.6),Caulobacter(99.3)	k__Bacteria;p__Proteobacteria;c__Alphaproteobacteria;o__Caulobacterales;f__Caulobacteraceae;g__Caulobacter;s__	1,00

bc03_D06;OTUwell_2	Bacteria(100.0),"Proteobacteria"(100.0),Alphaproteobacteria(98.8),Rhizobiales(98.8),Rhizobiaceae(98.8),Rhizobium(98.8)	k__Bacteria;p__Proteobacteria;c__Alphaproteobacteria;o__Rhizobiales;f__Rhizobiaceae;g__Rhizobium;s__	1,00
bc03_D11;OTUwell_1	Bacteria(100.0),"Bacteroidetes"(100.0),"Sphingobacteria"(99.3),"Sphingobacteriales"(99.3),Sphingobacteriaceae(99.3),Mucilaginibacter(99.3)	k__Bacteria;p__Bacteroidetes;c__Sphingobacteriia;o__Sphingobacteriales;f__Sphingobacteriaceae;g__;s__	1,00
bc03_D11;OTUwell_2	Bacteria(100.0),"Bacteroidetes"(100.0),"Sphingobacteria"(98.2),"Sphingobacteriales"(98.2),Sphingobacteriaceae(98.2),Mucilaginibacter(95.9)	k__Bacteria;p__Bacteroidetes;c__Sphingobacteriia;o__Sphingobacteriales;f__Sphingobacteriaceae;g__;s__	1,00
bc03_D11;OTUwell_3	Bacteria(100.0),"Proteobacteria"(100.0),Alphaproteobacteria(99.6),Caulobacterales(99.6),Caulobacteraceae(99.6),Caulobacter(99.2)	k__Bacteria;p__Proteobacteria;c__Alphaproteobacteria;o__Caulobacterales;f__Caulobacteraceae;g__Caulobacter;s__	1,00
bc03_D12;OTUwell_1	Bacteria(100.0),"Bacteroidetes"(100.0),"Sphingobacteria"(98.7),"Sphingobacteriales"(98.7),Sphingobacteriaceae(98.7),Pedobacter(98.7)	k__Bacteria;p__Bacteroidetes;c__Sphingobacteriia;o__Sphingobacteriales;f__Sphingobacteriaceae;g__Pedobacter;s__	1,00
bc03_D12;OTUwell_2	Bacteria(100.0),"Proteobacteria"(100.0),Betaproteobacteria(99.1),Burkholderiales(99.1),Burkholderiaceae(98.9),Burkholderia(96.3)	k__Bacteria;p__Proteobacteria;c__Betaproteobacteria;o__Burkholderiales;f__Burkholderiaceae;g__Burkholderia;s__	0,98
bc03_E01;OTUwell_1	Bacteria(100.0),"Proteobacteria"(100.0),Betaproteobacteria(99.0),Burkholderiales(99.0),Burkholderiaceae(99.0),Burkholderia(98.0)	k__Bacteria;p__Proteobacteria;c__Betaproteobacteria;o__Burkholderiales;f__Burkholderiaceae;g__Burkholderia	1,00
bc03_E01;OTUwell_2	Bacteria(100.0),"Proteobacteria"(100.0),Betaproteobacteria(99.1),Burkholderiales(99.1),Burkholderiaceae(98.9),Wautersia(89.1)	k__Bacteria;p__Proteobacteria;c__Betaproteobacteria;o__Burkholderiales;f__Oxalobacteraceae;g__Cupriavidus;s__	1,00
bc03_E01;OTUwell_3	Bacteria(100.0),"Bacteroidetes"(100.0),"Sphingobacteria"(99.8),"Sphingobacteriales"(99.6),Chitinophagaceae(99.6),Chitinophaga(99.6)	k__Bacteria;p__Bacteroidetes;c__[Saprospirae];o__[Saprospirales];f__Chitinophagaceae;g__Chitinophaga;s__	1,00
bc03_E01;OTUwell_4	Bacteria(100.0),Firmicutes(100.0),Bacilli(99.2),Bacillales(99.1),Bacillaceae_1(98.6),Bacillus(98.0)	k__Bacteria;p__Firmicutes;c__Bacilli;o__Bacillales;f__Bacillaceae;g__Bacillus;s__safensis	0,80
bc03_E01;OTUwell_5	Bacteria(100.0),"Bacteroidetes"(100.0),"Sphingobacteria"(99.9),"Sphingobacteriales"(99.9),Chitinophagaceae(99.9),Chitinophaga(99.9)	k__Bacteria;p__Bacteroidetes;c__[Saprospirae];o__[Saprospirales];f__Chitinophagaceae;g__Chitinophaga;s__	1,00
bc03_E01;OTUwell_6	Bacteria(100.0),"Bacteroidetes"(100.0),"Sphingobacteria"(98.2),"Sphingobacteriales"(98.2),Sphingobacteriaceae(98.2),Mucilaginibacter(95.9)	k__Bacteria;p__Bacteroidetes;c__Sphingobacteriia;o__Sphingobacteriales;f__Sphingobacteriaceae;g__;s__	1,00
bc03_E02;OTUwell_1	Bacteria(100.0),"Proteobacteria"(100.0),Alphaproteobacteria(98.4),Rhizobiales(98.4),Rhizobiaceae(98.4),Rhizobium(98.4)	k__Bacteria;p__Proteobacteria;c__Alphaproteobacteria;o__Rhizobiales;f__Rhizobiaceae;g__Rhizobium;s__	1,00
bc03_E03;OTUwell_1	Bacteria(100.0),"Bacteroidetes"(100.0),"Sphingobacteria"(100.0),"Sphingobacteriales"(100.0),Chitinophagaceae(100.0),Chitinophaga(99.9)	k__Bacteria;p__Bacteroidetes;c__[Saprospirae];o__[Saprospirales];f__Chitinophagaceae;g__Chitinophaga;s__	1,00
bc03_E04;OTUwell_1	Bacteria(100.0),"Proteobacteria"(100.0),Betaproteobacteria(99.2),Burkholderiales(99.2),Comamonadaceae(98.3),Variovorax(97.1)	k__Bacteria;p__Proteobacteria;c__Betaproteobacteria;o__Burkholderiales;f__Comamonadaceae;g__Variovorax;s__paradoxus	0,98
bc03_E05;OTUwell_1	Bacteria(100.0),"Bacteroidetes"(100.0),"Sphingobacteria"(99.3),"Sphingobacteriales"(99.3),Sphingobacteriaceae(99.3),Mucilaginibacter(99.3)	k__Bacteria;p__Bacteroidetes;c__Sphingobacteriia;o__Sphingobacteriales;f__Sphingobacteriaceae;g__;s__	1,00
bc03_E06;OTUwell_1	Bacteria(100.0),"Bacteroidetes"(100.0),"Sphingobacteria"(98.2),"Sphingobacteriales"(98.2),Sphingobacteriaceae(98.2),Mucilaginibacter(95.9)	k__Bacteria;p__Bacteroidetes;c__Sphingobacteriia;o__Sphingobacteriales;f__Sphingobacteriaceae;g__;s__	1,00
bc03_E08;OTUwell_1	Bacteria(100.0),"Bacteroidetes"(100.0),"Sphingobacteria"(100.0),"Sphingobacteriales"(100.0),Chitinophagaceae(100.0),Chitinophaga(99.9)	k__Bacteria;p__Bacteroidetes;c__[Saprospirae];o__[Saprospirales];f__Chitinophagaceae;g__Chitinophaga;s__	1,00
bc03_E08;OTUwell_2	Bacteria(100.0),"Bacteroidetes"(100.0),"Sphingobacteria"(100.0),"Sphingobacteriales"(100.0),Chitinophagaceae(99.6),Chitinophaga(99.0)	k__Bacteria;p__Bacteroidetes;c__[Saprospirae];o__[Saprospirales];f__Chitinophagaceae;g__Chitinophaga;s__	1,00
bc03_E09;OTUwell_1	Bacteria(100.0),"Proteobacteria"(100.0),Betaproteobacteria(98.9),Burkholderiales(98.9),Comamonadaceae(97.5),Variovorax(95.0)	k__Bacteria;p__Proteobacteria;c__Betaproteobacteria;o__Burkholderiales;f__Comamonadaceae;g__Variovorax;s__paradoxus	0,88
bc03_E10;OTUwell_1	Bacteria(100.0),"Bacteroidetes"(100.0),"Sphingobacteria"(99.3),"Sphingobacteriales"(99.3),Sphingobacteriaceae(99.3),Mucilaginibacter(99.3)	k__Bacteria;p__Bacteroidetes;c__Sphingobacteriia;o__Sphingobacteriales;f__Sphingobacteriaceae;g__;s__	1,00
bc03_E11;OTUwell_1	Bacteria(100.0),"Proteobacteria"(100.0),Alphaproteobacteria(99.5),Caulobacterales(99.5),Caulobacteraceae(99.5),Caulobacter(97.5)	k__Bacteria;p__Proteobacteria;c__Alphaproteobacteria;o__Caulobacterales;f__Caulobacteraceae;g__Caulobacter;s__	1,00
bc03_E12;OTUwell_1	Bacteria(100.0),"Proteobacteria"(100.0),Betaproteobacteria(99.1),Burkholderiales(99.1),Burkholderiaceae(98.9),Burkholderia(96.3)	k__Bacteria;p__Proteobacteria;c__Betaproteobacteria;o__Burkholderiales;f__Burkholderiaceae;g__Burkholderia;s__	0,97
bc03_E12;OTUwell_2	Bacteria(100.0),"Bacteroidetes"(100.0),"Sphingobacteria"(98.6),"Sphingobacteriales"(98.6),Sphingobacteriaceae(98.6),Pedobacter(98.6)	k__Bacteria;p__Bacteroidetes;c__Sphingobacteriia;o__Sphingobacteriales;f__Sphingobacteriaceae;g__Pedobacter;s__	1,00
bc03_E12;OTUwell_3	Bacteria(100.0),"Proteobacteria"(100.0),Betaproteobacteria(99.0),Burkholderiales(99.0),Burkholderiaceae(98.8),Burkholderia(97.7)	k__Bacteria;p__Proteobacteria;c__Betaproteobacteria;o__Burkholderiales;f__Burkholderiaceae;g__Burkholderia	0,90
bc03_F01;OTUwell_1	Bacteria(100.0),"Bacteroidetes"(100.0),"Sphingobacteria"(98.2),"Sphingobacteriales"(98.2),Sphingobacteriaceae(98.2),Mucilaginibacter(95.9)	k__Bacteria;p__Bacteroidetes;c__Sphingobacteriia;o__Sphingobacteriales;f__Sphingobacteriaceae;g__;s__	1,00
bc03_F01;OTUwell_2	Bacteria(100.0),"Bacteroidetes"(100.0),"Sphingobacteria"(99.8),"Sphingobacteriales"(99.6),Chitinophagaceae(99.6),Chitinophaga(99.6)	k__Bacteria;p__Bacteroidetes;c__[Saprospirae];o__[Saprospirales];f__Chitinophagaceae;g__Chitinophaga;s__	1,00
bc03_F01;OTUwell_3	Bacteria(100.0),"Proteobacteria"(100.0),Betaproteobacteria(99.3),Burkholderiales(99.3),Burkholderiaceae(99.3),Burkholderia(99.0)	k__Bacteria;p__Proteobacteria;c__Betaproteobacteria;o__Burkholderiales;f__Burkholderiaceae;g__Burkholderia;s__	1,00
bc03_F03;OTUwell_1	Bacteria(100.0),"Proteobacteria"(100.0),Betaproteobacteria(99.0),Burkholderiales(99.0),Burkholderiaceae(98.7),Burkholderia(98.0)	k__Bacteria;p__Proteobacteria;c__Betaproteobacteria;o__Burkholderiales;f__Burkholderiaceae;g__Burkholderia;s__	0,91
bc03_F03;OTUwell_2	Bacteria(100.0),"Proteobacteria"(100.0),Betaproteobacteria(97.3),Burkholderiales(95.9),Burkholderiaceae(94.6),Chitinimonas(85.8)	k__Bacteria;p__Proteobacteria;c__Betaproteobacteria;o__Neisseriales;f__Neisseriaceae;g__;s__	0,99
bc03_F04;OTUwell_1	Bacteria(100.0),"Proteobacteria"(100.0),Betaproteobacteria(98.9),Burkholderiales(98.9),Comamonadaceae(97.7),Variovorax(95.4)	k__Bacteria;p__Proteobacteria;c__Betaproteobacteria;o__Burkholderiales;f__Comamonadaceae;g__Variovorax;s__paradoxus	0,97
bc03_F05;OTUwell_1	Bacteria(100.0),"Proteobacteria"(100.0),Betaproteobacteria(99.5),Burkholderiales(99.5),Burkholderiaceae(99.5),Burkholderia(99.4)	k__Bacteria;p__Proteobacteria;c__Betaproteobacteria;o__Burkholderiales;f__Burkholderiaceae;g__Burkholderia;s__	0,94
bc03_F06;OTUwell_1	Bacteria(100.0),"Proteobacteria"(100.0),Alphaproteobacteria(98.5),Rhizobiales(98.5),Rhizobiaceae(98.5),Ensifer(97.5)	k__Bacteria;p__Proteobacteria;c__Alphaproteobacteria;o__Rhizobiales;f__Rhizobiaceae;g__;s__	0,90
bc03_F07;OTUwell_1	Bacteria(100.0),"Bacteroidetes"(100.0),"Sphingobacteria"(99.3),"Sphingobacteriales"(99.3),Sphingobacteriaceae(99.3),Mucilaginibacter(99.3)	k__Bacteria;p__Bacteroidetes;c__Sphingobacteriia;o__Sphingobacteriales;f__Sphingobacteriaceae;g__;s__	1,00
bc03_F07;OTUwell_2	Bacteria(100.0),"Proteobacteria"(100.0),Betaproteobacteria(99.2),Burkholderiales(99.2),Comamonadaceae(98.3),Variovorax(97.1)	k__Bacteria;p__Proteobacteria;c__Betaproteobacteria;o__Burkholderiales;f__Comamonadaceae;g__Variovorax	0,85
bc03_F09;OTUwell_1	Bacteria(100.0),"Proteobacteria"(100.0),Betaproteobacteria(98.7),Burkholderiales(98.7),Comamonadaceae(97.4),Variovorax(93.8)	k__Bacteria;p__Proteobacteria;c__Betaproteobacteria;o__Burkholderiales;f__Comamonadaceae;g__Variovorax;s__paradoxus	0,94
bc03_F09;OTUwell_2	Bacteria(100.0),"Proteobacteria"(100.0),Alphaproteobacteria(98.6),Rhizobiales(98.6),Rhizobiaceae(98.6),Rhizobium(98.6)	k__Bacteria;p__Proteobacteria;c__Alphaproteobacteria;o__Rhizobiales;f__Rhizobiaceae;g__Rhizobium;s__	0,89
bc03_F10;OTUwell_1	Bacteria(100.0),"Proteobacteria"(100.0),Gammaproteobacteria(100.0),"Enterobacteriales"(99.1),Enterobacteriaceae(98.8),Enterobacter(95.4)	k__Bacteria;p__Proteobacteria;c__Gammaproteobacteria;o__Enterobacteriales;f__Enterobacteriaceae	1,00
bc03_F10;OTUwell_2	Bacteria(100.0),"Proteobacteria"(100.0),Gammaproteobacteria(100.0),"Enterobacteriales"(98.6),Enterobacteriaceae(97.7),Enterobacter(86.9)	k__Bacteria;p__Proteobacteria;c__Gammaproteobacteria;o__Enterobacteriales;f__Enterobacteriaceae;g__;s__	1,00
bc03_F10;OTUwell_3	Bacteria(100.0),"Proteobacteria"(100.0),Gammaproteobacteria(95.4),Pseudomonadales(95.4),Pseudomonadaceae(94.6),Rhizobacter(84.7)	k__Bacteria;p__Proteobacteria;c__Betaproteobacteria;o__Burkholderiales;f__Comamonadaceae;g__Methylibium;s__	0,97
bc03_F11;OTUwell_1	Bacteria(100.0),"Proteobacteria"(100.0),Betaproteobacteria(99.0),Burkholderiales(99.0),Burkholderiaceae(98.8),Cupriavidus(91.7)	k__Bacteria;p__Proteobacteria;c__Betaproteobacteria;o__Burkholderiales;f__Oxalobacteraceae;g__Cupriavidus;s__	1,00
bc03_F11;OTUwell_2	Bacteria(100.0),"Proteobacteria"(100.0),Betaproteobacteria(98.6),Burkholderiales(98.6),Burkholderiaceae(98.3),Cupriavidus(85.8)	k__Bacteria;p__Proteobacteria;c__Betaproteobacteria;o__Burkholderiales;f__Oxalobacteraceae;g__Cupriavidus;s__	1,00
bc03_F12;OTUwell_1	Bacteria(100.0),"Proteobacteria"(100.0),Betaproteobacteria(99.0),Burkholderiales(99.0),Comamonadaceae(97.5),Variovorax(95.0)	k__Bacteria;p__Proteobacteria;c__Betaproteobacteria;o__Burkholderiales;f__Comamonadaceae	1,00
bc03_F12;OTUwell_2	Bacteria(100.0),"Proteobacteria"(100.0),Gammaproteobacteria(99.8),Xanthomonadales(99.2),Xanthomonadaceae(99.0),Lysobacter(99.0)	k__Bacteria;p__Proteobacteria;c__Gammaproteobacteria;o__Xanthomonadales;f__Xanthomonadaceae;g__Lysobacter;s__	1,00
bc03_F12;OTUwell_3	Bacteria(100.0),"Proteobacteria"(100.0),Alphaproteobacteria(98.9),Rhizobiales(98.9),Rhizobiaceae(98.9),Rhizobium(98.9)	k__Bacteria;p__Proteobacteria;c__Alphaproteobacteria;o__Rhizobiales;f__Rhizobiaceae;g__Rhizobium;s__	1,00
bc03_G01;OTUwell_1	Bacteria(100.0),"Proteobacteria"(100.0),Betaproteobacteria(99.5),Burkholderiales(99.5),Burkholderiaceae(99.5),Burkholderia(99.4)	k__Bacteria;p__Proteobacteria;c__Betaproteobacteria;o__Burkholderiales;f__Burkholderiaceae;g__Burkholderia;s__	0,91
bc03_G01;OTUwell_2	Bacteria(100.0),"Proteobacteria"(100.0),Gammaproteobacteria(99.3),Xanthomonadales(99.2),Xanthomonadaceae(99.0),Fulvimonas(86.9)	k__Bacteria;p__Proteobacteria;c__Gammaproteobacteria;o__Xanthomonadales;f__Xanthomonadaceae;g__Dyella;s__	0,96
bc03_G01;OTUwell_3	Bacteria(100.0),"Proteobacteria"(100.0),Gammaproteobacteria(99.8),Xanthomonadales(99.5),Xanthomonadaceae(99.5),Dokdonella(99.5)	k__Bacteria;p__Proteobacteria;c__Gammaproteobacteria;o__Xanthomonadales;f__Xanthomonadaceae;g__Dokdonella;s__	1,00
bc03_G01;OTUwell_4	Bacteria(100.0),"Proteobacteria"(100.0),Betaproteobacteria(98.5),Burkholderiales(98.5),Burkholderiaceae(98.2),Burkholderia(95.0)	k__Bacteria;p__Proteobacteria;c__Betaproteobacteria;o__Burkholderiales;f__Burkholderiaceae;g__Burkholderia	0,99
bc03_G01;OTUwell_5	Bacteria(100.0),"Bacteroidetes"(100.0),"Sphingobacteria"(99.8),"Sphingobacteriales"(99.7),Chitinophagaceae(99.7),Chitinophaga(99.7)	k__Bacteria;p__Bacteroidetes;c__[Saprospirae];o__[Saprospirales];f__Chitinophagaceae;g__Chitinophaga;s__	1,00
bc03_G01;OTUwell_6	Bacteria(100.0),"Proteobacteria"(100.0),Betaproteobacteria(98.6),Burkholderiales(98.6),Burkholderiaceae(98.3),Burkholderia(95.4)	k__Bacteria;p__Proteobacteria;c__Betaproteobacteria;o__Burkholderiales;f__Burkholderiaceae;g__Burkholderia;s__	1,00
bc03_G01;OTUwell_7	Bacteria(100.0),"Bacteroidetes"(100.0),"Sphingobacteria"(98.2),"Sphingobacteriales"(98.2),Sphingobacteriaceae(98.2),Mucilaginibacter(96.3)	k__Bacteria;p__Bacteroidetes;c__Sphingobacteriia;o__Sphingobacteriales;f__Sphingobacteriaceae;g__;s__	1,00
bc03_G01;OTUwell_8	Bacteria(100.0),"Proteobacteria"(100.0),Betaproteobacteria(98.7),Burkholderiales(98.7),Burkholderiaceae(98.6),Burkholderia(96.3)	k__Bacteria;p__Proteobacteria;c__Betaproteobacteria;o__Burkholderiales;f__Burkholderiaceae;g__Burkholderia;s__	0,88
bc03_G01;OTUwell_9	Bacteria(100.0),"Proteobacteria"(100.0),Alphaproteobacteria(99.1),Rhodospirillales(99.1),Rhodospirillaceae(99.0),Inquilinus(99.0)	k__Bacteria;p__Proteobacteria;c__Alphaproteobacteria;o__Rhodospirillales;f__Rhodospirillaceae;g__Inquilinus;s__limosus	1,00
bc03_G02;OTUwell_1	Bacteria(100.0),"Bacteroidetes"(100.0),"Sphingobacteria"(99.9),"Sphingobacteriales"(99.9),Chitinophagaceae(99.9),Chitinophaga(99.9)	k__Bacteria;p__Bacteroidetes;c__[Saprospirae];o__[Saprospirales];f__Chitinophagaceae;g__Chitinophaga;s__	1,00
bc03_G03;OTUwell_1	Bacteria(100.0),"Proteobacteria"(100.0),Alphaproteobacteria(98.6),Rhizobiales(98.6),Rhizobiaceae(98.5),Kaistia(98.5)	k__Bacteria;p__Proteobacteria;c__Alphaproteobacteria;o__Rhizobiales;f__Rhizobiaceae;g__Kaistia;s__	1,00
bc03_G03;OTUwell_2	Bacteria(100.0),"Proteobacteria"(100.0),Betaproteobacteria(98.7),Burkholderiales(98.7),Burkholderiaceae(98.3),Burkholderia(96.3)	k__Bacteria;p__Proteobacteria;c__Betaproteobacteria;o__Burkholderiales;f__Burkholderiaceae;g__Burkholderia;s__	0,86
bc03_G04;OTUwell_1	Bacteria(100.0),"Proteobacteria"(100.0),Gammaproteobacteria(99.6),Xanthomonadales(99.5),Xanthomonadaceae(99.5),Dyella(95.0)	k__Bacteria;p__Proteobacteria;c__Gammaproteobacteria;o__Xanthomonadales;f__Xanthomonadaceae;g__Dyella;s__	0,98
bc03_G04;OTUwell_2	Bacteria(100.0),"Proteobacteria"(100.0),Gammaproteobacteria(99.0),Xanthomonadales(99.0),Xanthomonadaceae(98.5),Dyella(79.9)	k__Bacteria;p__Proteobacteria;c__Gammaproteobacteria;o__Xanthomonadales;f__Xanthomonadaceae;g__Dyella;s__	1,00
bc03_G04;OTUwell_3	Bacteria(100.0),"Proteobacteria"(100.0),Betaproteobacteria(99.0),Burkholderiales(99.0),Comamonadaceae(97.8),Variovorax(95.9)	k__Bacteria;p__Proteobacteria;c__Betaproteobacteria;o__Burkholderiales;f__Comamonadaceae;g__Variovorax;s__paradoxus	0,92
bc03_G05;OTUwell_1	Bacteria(100.0),"Proteobacteria"(100.0),Gammaproteobacteria(99.6),Xanthomonadales(99.5),Sinobacteraceae(99.5),Singularimonas(92.4)	k__Bacteria;p__Proteobacteria;c__Gammaproteobacteria;o__Xanthomonadales;f__Sinobacteraceae;g__;s__	1,00
bc03_G07;OTUwell_1	Bacteria(100.0),"Proteobacteria"(100.0),Alphaproteobacteria(98.5),Rhizobiales(98.5),Rhizobiaceae(98.5),Rhizobium(98.5)	k__Bacteria;p__Proteobacteria;c__Alphaproteobacteria;o__Rhizobiales;f__Rhizobiaceae;g__Rhizobium;s__	0,99
bc03_G09;OTUwell_1	Bacteria(100.0),"Bacteroidetes"(100.0),"Sphingobacteria"(99.6),"Sphingobacteriales"(99.6),Chitinophagaceae(99.6),Chitinophaga(99.1)	k__Bacteria;p__Bacteroidetes;c__[Saprospirae];o__[Saprospirales];f__Chitinophagaceae;g__Chitinophaga;s__	1,00
bc03_G09;OTUwell_2	Bacteria(100.0),Firmicutes(100.0),Bacilli(99.7),Bacillales(99.5),Bacillaceae_1(99.2),Bacillus(99.2)	k__Bacteria;p__Firmicutes;c__Bacilli;o__Bacillales;f__Bacillaceae;g__Bacillus	1,00
bc03_G10;OTUwell_1	Bacteria(100.0),"Proteobacteria"(100.0),Gammaproteobacteria(99.6),Xanthomonadales(99.4),Xanthomonadaceae(99.4),Dyella(93.2)	k__Bacteria;p__Proteobacteria;c__Gammaproteobacteria;o__Xanthomonadales;f__Xanthomonadaceae;g__Dyella;s__	1,00
bc03_G10;OTUwell_2	Bacteria(100.0),"Bacteroidetes"(100.0),"Sphingobacteria"(98.2),"Sphingobacteriales"(98.2),Sphingobacteriaceae(98.2),Mucilaginibacter(96.3)	k__Bacteria;p__Bacteroidetes;c__Sphingobacteriia;o__Sphingobacteriales;f__Sphingobacteriaceae;g__;s__	1,00
bc03_G11;OTUwell_1	Bacteria(100.0),"Proteobacteria"(100.0),Alphaproteobacteria(98.9),Rhizobiales(98.9),Rhizobiaceae(98.9),Rhizobium(98.9)	k__Bacteria;p__Proteobacteria;c__Alphaproteobacteria;o__Rhizobiales;f__Rhizobiaceae;g__Rhizobium;s__	1,00
bc03_G12;OTUwell_1	Bacteria(100.0),"Proteobacteria"(100.0),Alphaproteobacteria(98.9),Rhizobiales(98.9),Rhizobiaceae(98.9),Rhizobium(98.9)	k__Bacteria;p__Proteobacteria;c__Alphaproteobacteria;o__Rhizobiales;f__Rhizobiaceae;g__Rhizobium;s__	1,00
bc03_G12;OTUwell_2	Bacteria(100.0),"Proteobacteria"(100.0),Betaproteobacteria(99.0),Burkholderiales(99.0),Comamonadaceae(97.5),Variovorax(95.0)	k__Bacteria;p__Proteobacteria;c__Betaproteobacteria;o__Burkholderiales;f__Comamonadaceae	1,00
bc03_H01;OTUwell_1	Bacteria(100.0),"Proteobacteria"(100.0),Betaproteobacteria(99.5),Burkholderiales(99.5),Burkholderiaceae(99.5),Burkholderia(99.3)	k__Bacteria;p__Proteobacteria;c__Betaproteobacteria;o__Burkholderiales;f__Burkholderiaceae;g__Burkholderia;s__	0,93
bc03_H01;OTUwell_2	Bacteria(100.0),"Bacteroidetes"(100.0),"Sphingobacteria"(98.2),"Sphingobacteriales"(98.2),Sphingobacteriaceae(98.2),Mucilaginibacter(95.9)	k__Bacteria;p__Bacteroidetes;c__Sphingobacteriia;o__Sphingobacteriales;f__Sphingobacteriaceae;g__;s__	1,00
bc03_H01;OTUwell_3	Bacteria(100.0),"Bacteroidetes"(100.0),"Sphingobacteria"(99.8),"Sphingobacteriales"(99.6),Chitinophagaceae(99.6),Chitinophaga(99.6)	k__Bacteria;p__Bacteroidetes;c__[Saprospirae];o__[Saprospirales];f__Chitinophagaceae;g__Chitinophaga;s__	1,00
bc03_H01;OTUwell_4	Bacteria(100.0),"Proteobacteria"(100.0),Alphaproteobacteria(96.0),Rhizobiales(96.0),Rhizobiaceae(94.9),Rhizobium(85.8)	k__Bacteria;p__Proteobacteria;c__Alphaproteobacteria;o__Rhizobiales;f__Rhizobiaceae;g__Rhizobium;s__	0,98
bc03_H02;OTUwell_1	Bacteria(100.0),"Proteobacteria"(100.0),Gammaproteobacteria(99.2),Xanthomonadales(99.1),Xanthomonadaceae(98.8),Fulvimonas(84.7)	k__Bacteria;p__Proteobacteria;c__Gammaproteobacteria;o__Xanthomonadales;f__Xanthomonadaceae;g__Dyella;s__	1,00

bc03_H03;OTUwell_1	Bacteria(100.0),"Actinobacteria"(100.0),Actinobacteria(99.6),Actinomycetales(99.6),Micrococcaceae(98.6),Arthrobacter(95.4)	k__Bacteria;p__Actinobacteria;c__Actinobacteria;o__Actinomycetales;f__Micrococcaceae;g__;s__	1,00
bc03_H04;OTUwell_1	Bacteria(100.0),"Bacteroidetes"(100.0),"Sphingobacteria"(99.8),"Sphingobacteriales"(99.6),Chitinophagaceae(99.6),Chitinophaga(99.6)	k__Bacteria;p__Bacteroidetes;c__[Saprospirae];o__[Saprospirales];f__Chitinophagaceae;g__Chitinophaga;s__	1,00
bc03_H04;OTUwell_2	Bacteria(100.0),"Proteobacteria"(100.0),Alphaproteobacteria(99.3),Caulobacterales(99.3),Caulobacteraceae(99.3),Caulobacter(97.1)	k__Bacteria;p__Proteobacteria;c__Alphaproteobacteria;o__Caulobacterales;f__Caulobacteraceae;g__Caulobacter;s__	0,98
bc03_H05;OTUwell_1	Bacteria(100.0),"Bacteroidetes"(100.0),"Sphingobacteria"(98.2),"Sphingobacteriales"(98.2),Sphingobacteriaceae(98.2),Mucilaginibacter(96.3)	k__Bacteria;p__Bacteroidetes;c__Sphingobacteriia;o__Sphingobacteriales;f__Sphingobacteriaceae;g__;s__	1,00
bc03_H05;OTUwell_2	Bacteria(100.0),"Bacteroidetes"(100.0),"Sphingobacteria"(99.6),"Sphingobacteriales"(99.6),Chitinophagaceae(99.6),Chitinophaga(99.2)	k__Bacteria;p__Bacteroidetes;c__[Saprospirae];o__[Saprospirales];f__Chitinophagaceae;g__Chitinophaga;s__	1,00
bc03_H06;OTUwell_1	Bacteria(100.0),"Bacteroidetes"(100.0),"Sphingobacteria"(99.3),"Sphingobacteriales"(99.3),Sphingobacteriaceae(99.3),Mucilaginibacter(99.3)	k__Bacteria;p__Bacteroidetes;c__Sphingobacteriia;o__Sphingobacteriales;f__Sphingobacteriaceae;g__;s__	1,00
bc03_H06;OTUwell_2	Bacteria(100.0),"Bacteroidetes"(100.0),"Sphingobacteria"(100.0),"Sphingobacteriales"(100.0),Chitinophagaceae(100.0),Chitinophaga(99.6)	k__Bacteria;p__Bacteroidetes;c__[Saprospirae];o__[Saprospirales];f__Chitinophagaceae;g__Chitinophaga;s__	1,00
bc03_H07;OTUwell_1	Bacteria(100.0),"Proteobacteria"(100.0),Gammaproteobacteria(99.8),Xanthomonadales(99.2),Xanthomonadaceae(99.0),Lysobacter(99.0)	k__Bacteria;p__Proteobacteria;c__Gammaproteobacteria;o__Xanthomonadales;f__Xanthomonadaceae;g__Lysobacter;s__	1,00
bc03_H10;OTUwell_1	Bacteria(100.0),"Proteobacteria"(100.0),Gammaproteobacteria(100.0),"Enterobacteriales"(99.4),Enterobacteriaceae(99.4),Escherichia/Shigella(99.0)	k__Bacteria;p__Proteobacteria;c__Gammaproteobacteria;o__Enterobacteriales;f__Enterobacteriaceae;g__;s__	1,00
bc03_H11;OTUwell_1	Bacteria(100.0),"Proteobacteria"(100.0),Gammaproteobacteria(100.0),"Enterobacteriales"(99.4),Enterobacteriaceae(99.4),Escherichia/Shigella(98.9)	k__Bacteria;p__Proteobacteria;c__Gammaproteobacteria;o__Enterobacteriales;f__Enterobacteriaceae;g__;s__	1,00
bc03_H12;OTUwell_1	Bacteria(100.0),"Proteobacteria"(100.0),Gammaproteobacteria(100.0),"Enterobacteriales"(99.4),Enterobacteriaceae(99.4),Escherichia/Shigella(99.0)	k__Bacteria;p__Proteobacteria;c__Gammaproteobacteria;o__Enterobacteriales;f__Enterobacteriaceae;g__;s__	1,00
bc04_A01;OTUwell_1	Bacteria(100.0),"Bacteroidetes"(100.0),"Sphingobacteria"(99.6),"Sphingobacteriales"(99.6),Chitinophagaceae(99.6),Chitinophaga(99.1)	k__Bacteria;p__Bacteroidetes;c__[Saprospirae];o__[Saprospirales];f__Chitinophagaceae;g__Chitinophaga;s__	1,00
bc04_A02;OTUwell_1	Bacteria(100.0),"Proteobacteria"(100.0),Gammaproteobacteria(99.6),Pseudomonadales(99.3),Pseudomonadaceae(99.3),Pseudomonas(98.0)	k__Bacteria;p__Proteobacteria;c__Gammaproteobacteria;o__Pseudomonadales;f__Pseudomonadaceae;g__Pseudomonas;s__	0,84
bc04_A02;OTUwell_2	Bacteria(100.0),"Proteobacteria"(100.0),Gammaproteobacteria(99.2),Pseudomonadales(99.1),Pseudomonadaceae(98.7),Pseudomonas(94.5)	k__Bacteria;p__Proteobacteria;c__Gammaproteobacteria;o__Pseudomonadales;f__Pseudomonadaceae;g__Pseudomonas;s__	0,88
bc04_A02;OTUwell_3	Bacteria(100.0),"Proteobacteria"(100.0),Alphaproteobacteria(98.7),Rhizobiales(98.7),Rhizobiaceae(98.7),Rhizobium(98.7)	k__Bacteria;p__Proteobacteria;c__Alphaproteobacteria;o__Rhizobiales;f__Rhizobiaceae;g__Rhizobium;s__	0,99
bc04_A05;OTUwell_1	Bacteria(100.0),"Bacteroidetes"(100.0),"Sphingobacteria"(100.0),"Sphingobacteriales"(100.0),Chitinophagaceae(100.0),Chitinophaga(99.9)	k__Bacteria;p__Bacteroidetes;c__[Saprospirae];o__[Saprospirales];f__Chitinophagaceae;g__Chitinophaga;s__	1,00
bc04_A06;OTUwell_1	Bacteria(100.0),"Bacteroidetes"(100.0),Flavobacteria(97.9),"Flavobacteriales"(97.9),Flavobacteriaceae(97.9),Flavobacterium(97.1)	k__Bacteria;p__Bacteroidetes;c__Flavobacteriia;o__Flavobacteriales;f__Flavobacteriaceae;g__Flavobacterium;s__succinicans	0,89
bc04_A08;OTUwell_1	Bacteria(100.0),"Proteobacteria"(100.0),Gammaproteobacteria(100.0),"Enterobacteriales"(99.1),Enterobacteriaceae(98.7),Enterobacter(95.0)	k__Bacteria;p__Proteobacteria;c__Gammaproteobacteria;o__Enterobacteriales;f__Enterobacteriaceae	1,00
bc04_A09;OTUwell_1	Bacteria(100.0),"Proteobacteria"(100.0),Gammaproteobacteria(99.6),Xanthomonadales(99.4),Xanthomonadaceae(99.4),Dyella(93.8)	k__Bacteria;p__Proteobacteria;c__Gammaproteobacteria;o__Xanthomonadales;f__Xanthomonadaceae;g__Dyella;s__	1,00
bc04_A10;OTUwell_1	Bacteria(100.0),"Proteobacteria"(100.0),Betaproteobacteria(99.0),Burkholderiales(99.0),Comamonadaceae(97.8),Variovorax(95.9)	k__Bacteria;p__Proteobacteria;c__Betaproteobacteria;o__Burkholderiales;f__Comamonadaceae;g__Variovorax;s__paradoxus	0,91
bc04_A11;OTUwell_1	Bacteria(100.0),"Proteobacteria"(100.0),Alphaproteobacteria(98.6),Rhizobiales(98.6),Rhizobiaceae(98.6),Rhizobium(98.6)	k__Bacteria;p__Proteobacteria;c__Alphaproteobacteria;o__Rhizobiales;f__Rhizobiaceae;g__Rhizobium;s__	1,00
bc04_A11;OTUwell_2	Bacteria(100.0),"Proteobacteria"(100.0),Alphaproteobacteria(98.3),Rhizobiales(98.3),Rhizobiaceae(98.3),Rhizobium(98.3)	k__Bacteria;p__Proteobacteria;c__Alphaproteobacteria;o__Rhizobiales;f__Rhizobiaceae;g__Rhizobium;s__	0,89
bc04_A11;OTUwell_3	Bacteria(100.0),"Proteobacteria"(100.0),Betaproteobacteria(97.9),Burkholderiales(97.9),Oxalobacteraceae(97.8),Herbaspirillum(93.8)	k__Bacteria;p__Proteobacteria;c__Betaproteobacteria;o__Burkholderiales;f__Oxalobacteraceae;g__;s__	0,98
bc04_A11;OTUwell_4	Bacteria(100.0),"Bacteroidetes"(100.0),"Sphingobacteria"(100.0),"Sphingobacteriales"(100.0),Chitinophagaceae(100.0),Chitinophaga(99.5)	k__Bacteria;p__Bacteroidetes;c__[Saprospirae];o__[Saprospirales];f__Chitinophagaceae;g__Chitinophaga;s__	1,00
bc04_B02;OTUwell_1	Bacteria(100.0),"Proteobacteria"(100.0),Betaproteobacteria(99.0),Burkholderiales(99.0),Comamonadaceae(97.9),Variovorax(96.3)	k__Bacteria;p__Proteobacteria;c__Betaproteobacteria;o__Burkholderiales;f__Comamonadaceae;g__Variovorax;s__paradoxus	0,89
bc04_B03;OTUwell_1	Bacteria(100.0),"Proteobacteria"(100.0),Betaproteobacteria(99.4),Burkholderiales(99.4),Burkholderiaceae(99.4),Burkholderia(99.3)	k__Bacteria;p__Proteobacteria;c__Betaproteobacteria;o__Burkholderiales;f__Burkholderiaceae;g__Burkholderia;s__	1,00
bc04_B03;OTUwell_2	Bacteria(100.0),"Bacteroidetes"(100.0),"Sphingobacteria"(98.1),"Sphingobacteriales"(98.1),Sphingobacteriaceae(98.1),Mucilaginibacter(95.9)	k__Bacteria;p__Bacteroidetes;c__Sphingobacteriia;o__Sphingobacteriales;f__Sphingobacteriaceae;g__;s__	1,00
bc04_B04;OTUwell_1	Bacteria(100.0),"Proteobacteria"(100.0),Gammaproteobacteria(99.7),Xanthomonadales(99.5),Xanthomonadaceae(99.5),Dyella(98.0)	k__Bacteria;p__Proteobacteria;c__Gammaproteobacteria;o__Xanthomonadales;f__Xanthomonadaceae;g__Dyella;s__	1,00
bc04_B04;OTUwell_2	Bacteria(100.0),"Proteobacteria"(100.0),Gammaproteobacteria(99.2),Xanthomonadales(99.1),Xanthomonadaceae(98.8),Dyella(88.0)	k__Bacteria;p__Proteobacteria;c__Gammaproteobacteria;o__Xanthomonadales;f__Xanthomonadaceae;g__Dyella;s__	1,00
bc04_B05;OTUwell_1	Bacteria(100.0),"Bacteroidetes"(100.0),Flavobacteria(97.2),"Flavobacteriales"(97.2),Flavobacteriaceae(97.2),Flavobacterium(90.8)	k__Bacteria;p__Bacteroidetes;c__Flavobacteriia;o__Flavobacteriales;f__Flavobacteriaceae;g__Flavobacterium;s__	1,00
bc04_B06;OTUwell_1	Bacteria(100.0),"Bacteroidetes"(100.0),"Sphingobacteria"(99.6),"Sphingobacteriales"(99.6),Chitinophagaceae(99.6),Chitinophaga(99.0)	k__Bacteria;p__Bacteroidetes;c__[Saprospirae];o__[Saprospirales];f__Chitinophagaceae;g__Chitinophaga;s__	1,00
bc04_B07;OTUwell_1	Bacteria(100.0),"Bacteroidetes"(100.0),"Sphingobacteria"(99.2),"Sphingobacteriales"(99.2),Sphingobacteriaceae(99.2),Mucilaginibacter(99.2)	k__Bacteria;p__Bacteroidetes;c__Sphingobacteriia;o__Sphingobacteriales;f__Sphingobacteriaceae;g__;s__	1,00
bc04_B09;OTUwell_1	Bacteria(100.0),"Bacteroidetes"(100.0),Flavobacteria(98.2),"Flavobacteriales"(98.2),Flavobacteriaceae(98.2),Flavobacterium(95.4)	k__Bacteria;p__Bacteroidetes;c__Flavobacteriia;o__Flavobacteriales;f__Flavobacteriaceae;g__Flavobacterium;s__	1,00
bc04_B11;OTUwell_1	Bacteria(100.0),"Bacteroidetes"(100.0),"Sphingobacteria"(99.9),"Sphingobacteriales"(99.9),Chitinophagaceae(99.9),Chitinophaga(99.9)	k__Bacteria;p__Bacteroidetes;c__[Saprospirae];o__[Saprospirales];f__Chitinophagaceae;g__Chitinophaga;s__	1,00
bc04_C01;OTUwell_1	Bacteria(100.0),"Bacteroidetes"(100.0),Flavobacteria(98.0),"Flavobacteriales"(98.0),Flavobacteriaceae(98.0),Flavobacterium(95.0)	k__Bacteria;p__Bacteroidetes;c__Flavobacteriia;o__Flavobacteriales;f__Flavobacteriaceae;g__Flavobacterium;s__	1,00
bc04_C01;OTUwell_2	Bacteria(100.0),"Proteobacteria"(100.0),Betaproteobacteria(99.3),Burkholderiales(99.3),Burkholderiaceae(99.3),Wautersia(93.8)	k__Bacteria;p__Proteobacteria;c__Betaproteobacteria;o__Burkholderiales;f__Oxalobacteraceae;g__Cupriavidus;s__	1,00
bc04_C01;OTUwell_3	Bacteria(100.0),"Bacteroidetes"(100.0),"Sphingobacteria"(99.8),"Sphingobacteriales"(99.7),Chitinophagaceae(99.7),Chitinophaga(99.7)	k__Bacteria;p__Bacteroidetes;c__[Saprospirae];o__[Saprospirales];f__Chitinophagaceae;g__Chitinophaga;s__	1,00
bc04_C02;OTUwell_1	Bacteria(100.0),"Proteobacteria"(100.0),Alphaproteobacteria(99.1),Rhodospirillales(99.1),Rhodospirillaceae(99.1),Inquilinus(99.1)	k__Bacteria;p__Proteobacteria;c__Alphaproteobacteria;o__Rhodospirillales;f__Rhodospirillaceae;g__Inquilinus;s__limosus	1,00
bc04_C03;OTUwell_1	Bacteria(100.0),"Proteobacteria"(100.0),Alphaproteobacteria(99.6),Sphingomonadales(99.0),Sphingomonadaceae(98.5),Sphingomonas(98.5)	k__Bacteria;p__Proteobacteria;c__Alphaproteobacteria;o__Sphingomonadales;f__Sphingomonadaceae;g__Sphingomonas;s__	0,99
bc04_C04;OTUwell_1	Bacteria(100.0),"Proteobacteria"(100.0),Alphaproteobacteria(98.7),Rhizobiales(98.7),Rhizobiaceae(98.7),Rhizobium(98.7)	k__Bacteria;p__Proteobacteria;c__Alphaproteobacteria;o__Rhizobiales;f__Rhizobiaceae;g__Rhizobium;s__	0,99
bc04_C04;OTUwell_2	Bacteria(100.0),"Proteobacteria"(100.0),Gammaproteobacteria(99.5),Xanthomonadales(99.3),Xanthomonadaceae(99.2),Dyella(91.7)	k__Bacteria;p__Proteobacteria;c__Gammaproteobacteria;o__Xanthomonadales;f__Xanthomonadaceae;g__Dyella;s__	0,98
bc04_C05;OTUwell_1	Bacteria(100.0),"Proteobacteria"(100.0),Gammaproteobacteria(99.6),Xanthomonadales(99.4),Xanthomonadaceae(99.4),Dyella(94.5)	k__Bacteria;p__Proteobacteria;c__Gammaproteobacteria;o__Xanthomonadales;f__Xanthomonadaceae;g__Dyella;s__	1,00
bc04_C05;OTUwell_2	Bacteria(100.0),"Proteobacteria"(100.0),Alphaproteobacteria(99.4),Rhizobiales(99.4),Bradyrhizobiaceae(99.2),Bosea(99.2)	k__Bacteria;p__Proteobacteria;c__Alphaproteobacteria;o__Rhizobiales	1,00
bc04_C05;OTUwell_3	Bacteria(100.0),"Proteobacteria"(100.0),Gammaproteobacteria(98.7),Xanthomonadales(98.7),Sinobacteraceae(98.6),Sinobacter(77.6)	k__Bacteria;p__Proteobacteria;c__Gammaproteobacteria;o__Xanthomonadales;f__Sinobacteraceae;g__;s__	0,96
bc04_C05;OTUwell_4	Bacteria(100.0),"Bacteroidetes"(100.0),"Sphingobacteria"(100.0),"Sphingobacteriales"(100.0),Chitinophagaceae(100.0),Chitinophaga(99.6)	k__Bacteria;p__Bacteroidetes;c__[Saprospirae];o__[Saprospirales];f__Chitinophagaceae;g__Chitinophaga;s__	1,00
bc04_C05;OTUwell_5	Bacteria(100.0),"Proteobacteria"(100.0),Gammaproteobacteria(99.0),Xanthomonadales(99.0),Xanthomonadaceae(98.7),Dyella(83.4)	k__Bacteria;p__Proteobacteria;c__Gammaproteobacteria;o__Xanthomonadales;f__Xanthomonadaceae;g__Dyella;s__	1,00
bc04_C06;OTUwell_1	Bacteria(100.0),Firmicutes(100.0),Bacilli(99.6),Bacillales(99.4),Bacillaceae_1(99.0),Bacillus(99.0)	k__Bacteria;p__Firmicutes;c__Bacilli;o__Bacillales;f__Bacillaceae;g__Bacillus	1,00
bc04_C07;OTUwell_1	Bacteria(100.0),"Proteobacteria"(100.0),Gammaproteobacteria(99.7),Xanthomonadales(99.5),Xanthomonadaceae(99.5),Dyella(97.5)	k__Bacteria;p__Proteobacteria;c__Gammaproteobacteria;o__Xanthomonadales;f__Xanthomonadaceae;g__Dyella;s__	0,99
bc04_C07;OTUwell_2	Bacteria(100.0),"Bacteroidetes"(100.0),"Sphingobacteria"(99.6),"Sphingobacteriales"(99.6),Chitinophagaceae(99.6),Chitinophaga(99.2)	k__Bacteria;p__Bacteroidetes;c__[Saprospirae];o__[Saprospirales];f__Chitinophagaceae;g__Chitinophaga;s__	1,00
bc04_C08;OTUwell_1	Bacteria(100.0),"Bacteroidetes"(100.0),"Sphingobacteria"(98.7),"Sphingobacteriales"(98.7),Sphingobacteriaceae(98.7),Pedobacter(98.7)	k__Bacteria;p__Bacteroidetes;c__Sphingobacteriia;o__Sphingobacteriales;f__Sphingobacteriaceae;g__Pedobacter;s__	1,00
bc04_C08;OTUwell_2	Bacteria(100.0),"Proteobacteria"(100.0),Alphaproteobacteria(99.1),Rhodospirillales(99.1),Rhodospirillaceae(99.0),Inquilinus(99.0)	k__Bacteria;p__Proteobacteria;c__Alphaproteobacteria;o__Rhodospirillales;f__Rhodospirillaceae;g__Inquilinus;s__limosus	1,00
bc04_C09;OTUwell_1	Bacteria(100.0),"Proteobacteria"(100.0),Alphaproteobacteria(98.8),Rhizobiales(98.8),Rhizobiaceae(98.8),Rhizobium(98.8)	k__Bacteria;p__Proteobacteria;c__Alphaproteobacteria;o__Rhizobiales;f__Rhizobiaceae;g__Rhizobium;s__	0,99
bc04_C10;OTUwell_1	Bacteria(100.0),"Proteobacteria"(100.0),Alphaproteobacteria(99.1),Rhizobiales(99.1),Bradyrhizobiaceae(98.7),Bosea(98.7)	k__Bacteria;p__Proteobacteria;c__Alphaproteobacteria;o__Rhizobiales	1,00
bc04_C10;OTUwell_2	Bacteria(100.0),Firmicutes(100.0),Bacilli(98.9),Bacillales(98.9),Bacillaceae_1(98.3),Bacillus(98.3)	k__Bacteria;p__Firmicutes;c__Bacilli;o__Bacillales;f__Bacillaceae	0,88
bc04_C10;OTUwell_3	Bacteria(100.0),"Proteobacteria"(100.0),Alphaproteobacteria(98.9),Rhizobiales(98.9),Bradyrhizobiaceae(98.6),Bosea(98.6)	k__Bacteria;p__Proteobacteria;c__Alphaproteobacteria;o__Rhizobiales	1,00
bc04_C11;OTUwell_1	Bacteria(100.0),"Proteobacteria"(100.0),Gammaproteobacteria(99.6),Xanthomonadales(99.4),Xanthomonadaceae(99.4),Dyella(92.4)	k__Bacteria;p__Proteobacteria;c__Gammaproteobacteria;o__Xanthomonadales;f__Xanthomonadaceae;g__Dyella;s__	1,00
bc04_C11;OTUwell_2	Bacteria(100.0),"Proteobacteria"(100.0),Gammaproteobacteria(98.6),Xanthomonadales(98.6),Xanthomonadaceae(98.0),Dyella(75.9)	k__Bacteria;p__Proteobacteria;c__Gammaproteobacteria;o__Xanthomonadales;f__Xanthomonadaceae;g__Dyella;s__	1,00
bc04_C12;OTUwell_1	Bacteria(100.0),"Bacteroidetes"(100.0),"Sphingobacteria"(99.6),"Sphingobacteriales"(99.6),Chitinophagaceae(99.6),Chitinophaga(99.2)	k__Bacteria;p__Bacteroidetes;c__[Saprospirae];o__[Saprospirales];f__Chitinophagaceae;g__Chitinophaga;s__	1,00
bc04_D01;OTUwell_1	Bacteria(100.0),"Bacteroidetes"(100.0),"Sphingobacteria"(100.0),"Sphingobacteriales"(100.0),Cytophagaceae(99.5),Dyadobacter(99.5)	k__Bacteria;p__Bacteroidetes;c__Cytophagia;o__Cytophagales;f__Cytophagaceae;g__Dyadobacter;s__	1,00
bc04_D02;OTUwell_1	Bacteria(100.0),"Proteobacteria"(100.0),Gammaproteobacteria(100.0),"Enterobacteriales"(99.1),Enterobacteriaceae(98.7),Enterobacter(95.0)	k__Bacteria;p__Proteobacteria;c__Gammaproteobacteria;o__Enterobacteriales;f__Enterobacteriaceae;g__;s__	0,91
bc04_D03;OTUwell_1	Bacteria(100.0),"Actinobacteria"(100.0),Actinobacteria(100.0),Actinomycetales(99.6),Promicromonosporaceae(98.9),Promicromonospora(98.9)	k__Bacteria;p__Actinobacteria;c__Actinobacteria;o__Actinomycetales;f__Promicromonosporaceae;g__Promicromonospora;s__	1,00
bc04_D04;OTUwell_1	Bacteria(100.0),"Proteobacteria"(100.0),Alphaproteobacteria(99.4),Caulobacterales(99.4),Caulobacteraceae(99.4),Caulobacter(97.1)	k__Bacteria;p__Proteobacteria;c__Alphaproteobacteria;o__Caulobacterales;f__Caulobacteraceae;g__Caulobacter;s__	0,90
bc04_D05;OTUwell_1	Bacteria(100.0),"Proteobacteria"(100.0),Gammaproteobacteria(99.7),Xanthomonadales(99.1),Xanthomonadaceae(98.8),Lysobacter(98.8)	k__Bacteria;p__Proteobacteria;c__Gammaproteobacteria;o__Xanthomonadales;f__Xanthomonadaceae;g__Lysobacter;s__	1,00
bc04_D06;OTUwell_1	Bacteria(100.0),"Proteobacteria"(100.0),Alphaproteobacteria(98.9),Rhizobiales(98.9),Rhizobiaceae(98.9),Rhizobium(98.9)	k__Bacteria;p__Proteobacteria;c__Alphaproteobacteria;o__Rhizobiales;f__Rhizobiaceae;g__Rhizobium;s__	1,00
bc04_D06;OTUwell_2	Bacteria(100.0),"Proteobacteria"(100.0),Alphaproteobacteria(99.2),Caulobacterales(99.2),Caulobacteraceae(99.2),Caulobacter(97.7)	k__Bacteria;p__Proteobacteria;c__Alphaproteobacteria;o__Caulobacterales;f__Caulobacteraceae;g__Caulobacter;s__	1,00
bc04_D06;OTUwell_3	Bacteria(100.0),"Proteobacteria"(100.0),Gammaproteobacteria(99.6),Pseudomonadales(99.3),Pseudomonadaceae(99.3),Pseudomonas(97.7)	k__Bacteria;p__Proteobacteria;c__Gammaproteobacteria;o__Pseudomonadales;f__Pseudomonadaceae;g__Pseudomonas;s__	0,84
bc04_D07;OTUwell_1	Bacteria(100.0),"Proteobacteria"(100.0),Gammaproteobacteria(100.0),"Enterobacteriales"(99.1),Enterobacteriaceae(98.7),Enterobacter(95.0)	k__Bacteria;p__Proteobacteria;c__Gammaproteobacteria;o__Enterobacteriales;f__Enterobacteriaceae;g__;s__	0,85
bc04_D07;OTUwell_2	Bacteria(100.0),"Proteobacteria"(100.0),Gammaproteobacteria(100.0),"Enterobacteriales"(98.6),Enterobacteriaceae(97.7),Enterobacter(85.8)	k__Bacteria;p__Proteobacteria;c__Gammaproteobacteria;o__Enterobacteriales;f__Enterobacteriaceae;g__;s__	0,98
bc04_D07;OTUwell_3	Bacteria(100.0),Firmicutes(100.0),Bacilli(99.7),Bacillales(99.5),Bacillaceae_1(99.2),Bacillus(99.2)	k__Bacteria;p__Firmicutes;c__Bacilli;o__Bacillales;f__Bacillaceae;g__Bacillus	1,00
bc04_D07;OTUwell_4	Bacteria(100.0),"Proteobacteria"(100.0),Gammaproteobacteria(99.4),"Enterobacteriales"(98.1),Enterobacteriaceae(96.7),Enterobacter(84.7)	k__Bacteria;p__Proteobacteria;c__Gammaproteobacteria;o__Enterobacteriales;f__Enterobacteriaceae;g__;s__	0,98
bc04_D08;OTUwell_1	Bacteria(100.0),"Bacteroidetes"(100.0),"Sphingobacteria"(99.6),"Sphingobacteriales"(99.6),Chitinophagaceae(99.6),Chitinophaga(99.2)	k__Bacteria;p__Bacteroidetes;c__[Saprospirae];o__[Saprospirales];f__Chitinophagaceae;g__Chitinophaga;s__	1,00
bc04_D09;OTUwell_1	Bacteria(100.0),"Bacteroidetes"(100.0),"Sphingobacteria"(99.6),"Sphingobacteriales"(99.6),Chitinophagaceae(99.6),Chitinophaga(99.0)	k__Bacteria;p__Bacteroidetes;c__[Saprospirae];o__[Saprospirales];f__Chitinophagaceae;g__Chitinophaga;s__	1,00
bc04_D09;OTUwell_2	Bacteria(100.0),"Proteobacteria"(100.0),Betaproteobacteria(98.9),Burkholderiales(98.9),Burkholderiaceae(98.6),Burkholderia(97.7)	k__Bacteria;p__Proteobacteria;c__Betaproteobacteria;o__Burkholderiales;f__Burkholderiaceae	1,00
bc04_D12;OTUwell_1	Bacteria(100.0),"Proteobacteria"(100.0),Alphaproteobacteria(99.6),Sphingomonadales(99.1),Sphingomonadaceae(98.7),Sphingomonas(98.7)	k__Bacteria;p__Proteobacteria;c__Alphaproteobacteria;o__Sphingomonadales;f__Sphingomonadaceae;g__Sphingomonas;s__	1,00

bc04_D12;OTUwell_2	Bacteria(100.0),Firmicutes(100.0),Bacilli(99.7),Bacillales(99.5),Bacillaceae_1(99.4),Bacillus(99.4)	k__Bacteria;p__Firmicutes;c__Bacilli;o__Bacillales;f__Bacillaceae;g__;s__	0,99
bc04_E02;OTUwell_1	Bacteria(100.0),"Proteobacteria"(100.0),Alphaproteobacteria(98.8),Rhizobiales(98.8),Rhizobiaceae(98.8),Rhizobium(98.8)	k__Bacteria;p__Proteobacteria;c__Alphaproteobacteria;o__Rhizobiales;f__Rhizobiaceae;g__Rhizobium;s__	1,00
bc04_E02;OTUwell_2	Bacteria(100.0),"Proteobacteria"(100.0),Gammaproteobacteria(99.6),Xanthomonadales(98.7),Xanthomonadaceae(97.9),Lysobacter(89.1)	k__Bacteria;p__Proteobacteria;c__Gammaproteobacteria;o__Xanthomonadales;f__Xanthomonadaceae;g__Luteimonas;s__	0,94
bc04_E02;OTUwell_3	Bacteria(100.0),"Proteobacteria"(100.0),Gammaproteobacteria(99.4),Xanthomonadales(99.2),Xanthomonadaceae(99.1),Dyella(90.8)	k__Bacteria;p__Proteobacteria;c__Gammaproteobacteria;o__Xanthomonadales;f__Xanthomonadaceae;g__Dyella;s__	1,00
bc04_E03;OTUwell_1	Bacteria(100.0),"Proteobacteria"(100.0),Gammaproteobacteria(99.7),Pseudomonadales(99.5),Pseudomonadaceae(99.5),Pseudomonas(99.0)	k__Bacteria;p__Proteobacteria;c__Gammaproteobacteria;o__Pseudomonadales;f__Pseudomonadaceae;g__Pseudomonas;s__	0,89
bc04_E03;OTUwell_2	Bacteria(100.0),"Proteobacteria"(100.0),Betaproteobacteria(98.7),Burkholderiales(98.7),Burkholderiaceae(98.5),Burkholderia(96.6)	k__Bacteria;p__Proteobacteria;c__Betaproteobacteria;o__Burkholderiales;f__Burkholderiaceae;g__Burkholderia	0,84
bc04_E04;OTUwell_1	Bacteria(100.0),"Bacteroidetes"(100.0),"Sphingobacteria"(100.0),"Sphingobacteriales"(100.0),Cytophagaceae(99.5),Dyadobacter(99.5)	k__Bacteria;p__Bacteroidetes;c__Cytophagia;o__Cytophagales;f__Cytophagaceae;g__Dyadobacter;s__	1,00
bc04_E05;OTUwell_1	Bacteria(100.0),"Proteobacteria"(100.0),Alphaproteobacteria(98.9),Rhizobiales(98.9),Rhizobiaceae(98.9),Rhizobium(98.9)	k__Bacteria;p__Proteobacteria;c__Alphaproteobacteria;o__Rhizobiales;f__Rhizobiaceae;g__Rhizobium;s__	1,00
bc04_E06;OTUwell_1	Bacteria(100.0),"Proteobacteria"(100.0),Betaproteobacteria(99.1),Burkholderiales(99.1),Burkholderiaceae(98.9),Burkholderia(97.7)	k__Bacteria;p__Proteobacteria;c__Betaproteobacteria;o__Burkholderiales;f__Burkholderiaceae;g__Burkholderia;s__	0,99
bc04_E07;OTUwell_1	Bacteria(100.0),"Proteobacteria"(100.0),Alphaproteobacteria(98.6),Rhizobiales(98.6),Phyllobacteriaceae(98.6),Mesorhizobium(98.6)	k__Bacteria;p__Proteobacteria;c__Alphaproteobacteria;o__Rhizobiales;f__Phyllobacteriaceae;g__Mesorhizobium;s__	1,00
bc04_E08;OTUwell_1	Bacteria(100.0),"Proteobacteria"(100.0),Betaproteobacteria(97.2),Burkholderiales(95.3),Burkholderiaceae(94.2),Chitinimonas(84.7)	k__Bacteria;p__Proteobacteria;c__Betaproteobacteria;o__Neisseriales;f__Neisseriaceae;g__;s__	1,00
bc04_E09;OTUwell_1	Bacteria(100.0),"Proteobacteria"(100.0),Alphaproteobacteria(98.9),Rhizobiales(98.9),Rhizobiaceae(98.9),Rhizobium(98.9)	k__Bacteria;p__Proteobacteria;c__Alphaproteobacteria;o__Rhizobiales;f__Rhizobiaceae;g__Rhizobium;s__	1,00
bc04_E10;OTUwell_1	Bacteria(100.0),Firmicutes(100.0),Bacilli(99.6),Bacillales(99.5),Bacillaceae_1(99.4),Bacillus(99.4)	k__Bacteria;p__Firmicutes;c__Bacilli;o__Bacillales;f__Bacillaceae;g__Bacillus;s__flexus	1,00
bc04_E10;OTUwell_2	Bacteria(100.0),"Proteobacteria"(100.0),Alphaproteobacteria(99.2),Sphingomonadales(99.0),Sphingomonadaceae(98.5),Sphingobium(91.7)	k__Bacteria;p__Proteobacteria;c__Alphaproteobacteria;o__Sphingomonadales;f__Sphingomonadaceae;g__Sphingobium;s__	1,00
bc04_E10;OTUwell_3	Bacteria(100.0),Firmicutes(100.0),Bacilli(99.6),Bacillales(99.5),Bacillaceae_1(99.4),Bacillus(99.4)	k__Bacteria;p__Firmicutes;c__Bacilli;o__Bacillales;f__Bacillaceae;g__;s__	1,00
bc04_E10;OTUwell_4	Bacteria(100.0),Firmicutes(100.0),Bacilli(99.2),Bacillales(99.2),Bacillaceae_1(98.7),Bacillus(98.7)	k__Bacteria;p__Firmicutes;c__Bacilli;o__Bacillales;f__Bacillaceae;g__;s__	0,91
bc04_E11;OTUwell_1	Bacteria(100.0),"Proteobacteria"(100.0),Gammaproteobacteria(99.8),Xanthomonadales(99.5),Xanthomonadaceae(99.5),Dokdonella(99.5)	k__Bacteria;p__Proteobacteria;c__Gammaproteobacteria;o__Xanthomonadales;f__Xanthomonadaceae;g__Dokdonella;s__	1,00
bc04_E12;OTUwell_1	Bacteria(100.0),"Proteobacteria"(100.0),Betaproteobacteria(99.0),Burkholderiales(99.0),Comamonadaceae(97.8),Variovorax(95.9)	k__Bacteria;p__Proteobacteria;c__Betaproteobacteria;o__Burkholderiales;f__Comamonadaceae;g__Variovorax;s__paradoxus	0,90
bc04_F01;OTUwell_1	Bacteria(100.0),"Proteobacteria"(100.0),Alphaproteobacteria(98.8),Rhizobiales(98.8),Xanthobacteraceae(97.9),Ancylobacter(93.8)	k__Bacteria;p__Proteobacteria;c__Alphaproteobacteria;o__Rhizobiales;f__Xanthobacteraceae;g__;s__	0,90
bc04_F03;OTUwell_1	Bacteria(100.0),"Proteobacteria"(100.0),Gammaproteobacteria(96.9),Pseudomonadales(96.9),Pseudomonadaceae(96.5),Rhizobacter(93.2)	k__Bacteria;p__Proteobacteria;c__Betaproteobacteria;o__Burkholderiales;f__Comamonadaceae;g__Methylibium;s__	1,00
bc04_F04;OTUwell_1	Bacteria(100.0),"Proteobacteria"(100.0),Betaproteobacteria(99.0),Burkholderiales(99.0),Comamonadaceae(97.4),Variovorax(95.0)	k__Bacteria;p__Proteobacteria;c__Betaproteobacteria;o__Burkholderiales;f__Comamonadaceae;g__Variovorax;s__paradoxus	0,92
bc04_F04;OTUwell_2	Bacteria(100.0),"Proteobacteria"(100.0),Gammaproteobacteria(100.0),"Enterobacteriales"(99.1),Enterobacteriaceae(98.7),Enterobacter(94.5)	k__Bacteria;p__Proteobacteria;c__Gammaproteobacteria;o__Enterobacteriales;f__Enterobacteriaceae;g__;s__	0,81
bc04_F05;OTUwell_1	Bacteria(100.0),"Proteobacteria"(100.0),Gammaproteobacteria(99.4),Xanthomonadales(99.3),Xanthomonadaceae(99.2),Dyella(90.8)	k__Bacteria;p__Proteobacteria;c__Gammaproteobacteria;o__Xanthomonadales;f__Xanthomonadaceae;g__Dyella;s__	1,00
bc04_F05;OTUwell_2	Bacteria(100.0),Firmicutes(100.0),Bacilli(99.7),Bacillales(99.5),Bacillaceae_1(99.4),Bacillus(99.4)	k__Bacteria;p__Firmicutes;c__Bacilli;o__Bacillales;f__Bacillaceae;g__;s__	1,00
bc04_F05;OTUwell_3	Bacteria(100.0),Firmicutes(100.0),Bacilli(99.3),Bacillales(99.3),Bacillaceae_1(99.0),Bacillus(99.0)	k__Bacteria;p__Firmicutes;c__Bacilli;o__Bacillales;f__Bacillaceae;g__;s__	0,88
bc04_F05;OTUwell_4	Bacteria(100.0),"Proteobacteria"(100.0),Gammaproteobacteria(99.5),Xanthomonadales(99.3),Xanthomonadaceae(99.3),Dyella(92.4)	k__Bacteria;p__Proteobacteria;c__Gammaproteobacteria;o__Xanthomonadales;f__Xanthomonadaceae;g__Dyella;s__	1,00
bc04_F06;OTUwell_1	Bacteria(100.0),"Proteobacteria"(100.0),Alphaproteobacteria(97.2),Rhizobiales(97.2),Rhizobiaceae(97.0),Rhizobium(94.5)	k__Bacteria;p__Proteobacteria;c__Alphaproteobacteria;o__Rhizobiales;f__Rhizobiaceae;g__Agrobacterium;s__	1,00
bc04_F07;OTUwell_1	Bacteria(100.0),"Proteobacteria"(100.0),Gammaproteobacteria(99.3),Xanthomonadales(99.3),Xanthomonadaceae(99.1),Dyella(90.1)	k__Bacteria;p__Proteobacteria;c__Gammaproteobacteria;o__Xanthomonadales;f__Xanthomonadaceae;g__Dyella;s__	1,00
bc04_F07;OTUwell_2	Bacteria(100.0),"Proteobacteria"(100.0),Gammaproteobacteria(99.3),Xanthomonadales(99.3),Xanthomonadaceae(99.1),Dyella(90.8)	k__Bacteria;p__Proteobacteria;c__Gammaproteobacteria;o__Xanthomonadales;f__Xanthomonadaceae;g__Dyella;s__	1,00
bc04_F07;OTUwell_3	Bacteria(100.0),"Proteobacteria"(100.0),Betaproteobacteria(99.0),Burkholderiales(99.0),Comamonadaceae(97.8),Variovorax(96.6)	k__Bacteria;p__Proteobacteria;c__Betaproteobacteria;o__Burkholderiales;f__Comamonadaceae;g__Variovorax	0,83
bc04_F08;OTUwell_1	Bacteria(100.0),"Proteobacteria"(100.0),Betaproteobacteria(99.3),Burkholderiales(99.3),Burkholderiaceae(99.3),Burkholderia(99.2)	k__Bacteria;p__Proteobacteria;c__Betaproteobacteria;o__Burkholderiales;f__Burkholderiaceae;g__Burkholderia;s__	1,00
bc04_F08;OTUwell_2	Bacteria(100.0),"Proteobacteria"(100.0),Betaproteobacteria(98.5),Burkholderiales(98.5),Burkholderiaceae(98.5),Burkholderia(95.9)	k__Bacteria;p__Proteobacteria;c__Betaproteobacteria;o__Burkholderiales;f__Burkholderiaceae;g__Burkholderia;s__	0,91
bc04_F09;OTUwell_1	Bacteria(100.0),"Proteobacteria"(100.0),Alphaproteobacteria(98.6),Rhizobiales(98.6),Rhizobiaceae(98.4),Kaistia(98.4)	k__Bacteria;p__Proteobacteria;c__Alphaproteobacteria;o__Rhizobiales;f__Rhizobiaceae;g__Kaistia;s__	1,00
bc04_F11;OTUwell_1	Bacteria(100.0),"Bacteroidetes"(100.0),"Sphingobacteria"(99.9),"Sphingobacteriales"(99.9),Chitinophagaceae(99.9),Chitinophaga(99.9)	k__Bacteria;p__Bacteroidetes;c__[Saprospirae];o__[Saprospirales];f__Chitinophagaceae;g__Chitinophaga;s__	1,00
bc04_F11;OTUwell_2	Bacteria(100.0),"Bacteroidetes"(100.0),"Sphingobacteria"(99.8),"Sphingobacteriales"(99.6),Chitinophagaceae(99.6),Chitinophaga(99.6)	k__Bacteria;p__Bacteroidetes;c__[Saprospirae];o__[Saprospirales];f__Chitinophagaceae;g__Chitinophaga;s__	1,00
bc04_F12;OTUwell_1	Bacteria(100.0),"Proteobacteria"(100.0),Betaproteobacteria(99.3),Burkholderiales(99.3),Burkholderiaceae(99.3),Burkholderia(99.0)	k__Bacteria;p__Proteobacteria;c__Betaproteobacteria;o__Burkholderiales;f__Burkholderiaceae;g__Burkholderia;s__	0,99
bc04_F12;OTUwell_2	Bacteria(100.0),"Bacteroidetes"(100.0),"Sphingobacteria"(99.6),"Sphingobacteriales"(99.6),Chitinophagaceae(99.6),Filimonas(99.0)	k__Bacteria;p__Bacteroidetes;c__[Saprospirae];o__[Saprospirales];f__Chitinophagaceae;g__;s__	1,00
bc04_G01;OTUwell_1	Bacteria(100.0),"Bacteroidetes"(100.0),"Sphingobacteria"(98.2),"Sphingobacteriales"(98.2),Sphingobacteriaceae(98.2),Mucilaginibacter(95.9)	k__Bacteria;p__Bacteroidetes;c__Sphingobacteriia;o__Sphingobacteriales;f__Sphingobacteriaceae;g__;s__	1,00
bc04_G01;OTUwell_2	Bacteria(100.0),"Proteobacteria"(100.0),Gammaproteobacteria(99.8),Xanthomonadales(99.5),Xanthomonadaceae(99.5),Dokdonella(99.5)	k__Bacteria;p__Proteobacteria;c__Gammaproteobacteria;o__Xanthomonadales;f__Xanthomonadaceae;g__Dokdonella;s__	1,00
bc04_G02;OTUwell_1	Bacteria(100.0),"Proteobacteria"(100.0),Alphaproteobacteria(98.6),Rhizobiales(98.6),Xanthobacteraceae(96.4),Starkeya(90.1)	k__Bacteria;p__Proteobacteria;c__Alphaproteobacteria;o__Rhizobiales;f__Xanthobacteraceae;g__;s__	1,00
bc04_G03;OTUwell_1	Bacteria(100.0),Firmicutes(100.0),Bacilli(99.7),Bacillales(99.5),Bacillaceae_1(99.2),Bacillus(99.2)	k__Bacteria;p__Firmicutes;c__Bacilli;o__Bacillales;f__Bacillaceae;g__Bacillus	1,00
bc04_G03;OTUwell_2	Bacteria(100.0),"Bacteroidetes"(100.0),"Sphingobacteria"(99.6),"Sphingobacteriales"(99.6),Chitinophagaceae(99.6),Chitinophaga(99.2)	k__Bacteria;p__Bacteroidetes;c__[Saprospirae];o__[Saprospirales];f__Chitinophagaceae;g__Chitinophaga;s__	1,00
bc04_G03;OTUwell_3	Bacteria(100.0),Firmicutes(100.0),Bacilli(99.0),Bacillales(99.0),Bacillaceae_1(97.9),Bacillus(97.1)	k__Bacteria;p__Firmicutes;c__Bacilli;o__Bacillales;f__Bacillaceae;g__Bacillus	1,00
bc04_G04;OTUwell_1	Bacteria(100.0),"Proteobacteria"(100.0),Gammaproteobacteria(100.0),"Enterobacteriales"(99.1),Enterobacteriaceae(98.7),Enterobacter(94.5)	k__Bacteria;p__Proteobacteria;c__Gammaproteobacteria;o__Enterobacteriales;f__Enterobacteriaceae;g__;s__	0,85
bc04_G04;OTUwell_2	Bacteria(100.0),"Proteobacteria"(100.0),Betaproteobacteria(99.0),Burkholderiales(99.0),Comamonadaceae(97.9),Variovorax(95.9)	k__Bacteria;p__Proteobacteria;c__Betaproteobacteria;o__Burkholderiales;f__Comamonadaceae;g__Variovorax;s__paradoxus	0,87
bc04_G04;OTUwell_3	Bacteria(100.0),"Proteobacteria"(100.0),Gammaproteobacteria(100.0),"Enterobacteriales"(98.8),Enterobacteriaceae(98.0),Enterobacter(89.1)	k__Bacteria;p__Proteobacteria;c__Gammaproteobacteria;o__Enterobacteriales;f__Enterobacteriaceae;g__;s__	1,00
bc04_G05;OTUwell_1	Bacteria(100.0),"Proteobacteria"(100.0),Gammaproteobacteria(99.5),Xanthomonadales(99.4),Sinobacteraceae(99.4),Singularimonas(88.0)	k__Bacteria;p__Proteobacteria;c__Gammaproteobacteria;o__Xanthomonadales;f__Sinobacteraceae;g__;s__	0,97
bc04_G05;OTUwell_2	Bacteria(100.0),"Proteobacteria"(100.0),Alphaproteobacteria(98.4),Rhizobiales(98.4),Xanthobacteraceae(97.5),Ancylobacter(89.1)	k__Bacteria;p__Proteobacteria;c__Alphaproteobacteria;o__Rhizobiales;f__Xanthobacteraceae;g__;s__	1,00
bc04_G06;OTUwell_1	Bacteria(100.0),"Proteobacteria"(100.0),Alphaproteobacteria(98.9),Rhizobiales(98.9),Rhizobiaceae(98.9),Rhizobium(98.9)	k__Bacteria;p__Proteobacteria;c__Alphaproteobacteria;o__Rhizobiales;f__Rhizobiaceae;g__Rhizobium;s__	1,00
bc04_G06;OTUwell_2	Bacteria(100.0),"Proteobacteria"(100.0),Betaproteobacteria(99.5),Burkholderiales(99.5),Burkholderiaceae(99.5),Burkholderia(99.4)	k__Bacteria;p__Proteobacteria;c__Betaproteobacteria;o__Burkholderiales;f__Burkholderiaceae;g__Burkholderia;s__	1,00
bc04_G07;OTUwell_1	Bacteria(100.0),"Proteobacteria"(100.0),Alphaproteobacteria(98.9),Rhizobiales(98.9),Rhizobiaceae(98.9),Rhizobium(98.9)	k__Bacteria;p__Proteobacteria;c__Alphaproteobacteria;o__Rhizobiales;f__Rhizobiaceae;g__Rhizobium;s__	0,99
bc04_G08;OTUwell_1	Bacteria(100.0),"Proteobacteria"(100.0),Gammaproteobacteria(99.3),Xanthomonadales(99.2),Xanthomonadaceae(99.1),Dyella(90.8)	k__Bacteria;p__Proteobacteria;c__Gammaproteobacteria;o__Xanthomonadales;f__Xanthomonadaceae;g__Dyella;s__	1,00
bc04_G08;OTUwell_2	Bacteria(100.0),"Proteobacteria"(100.0),Gammaproteobacteria(99.5),Xanthomonadales(99.3),Xanthomonadaceae(99.2),Dyella(91.7)	k__Bacteria;p__Proteobacteria;c__Gammaproteobacteria;o__Xanthomonadales;f__Xanthomonadaceae;g__Dyella;s__	1,00
bc04_G08;OTUwell_3	Bacteria(100.0),"Proteobacteria"(100.0),Betaproteobacteria(99.2),Burkholderiales(99.2),Burkholderiaceae(99.2),Burkholderia(99.0)	k__Bacteria;p__Proteobacteria;c__Betaproteobacteria;o__Burkholderiales;f__Burkholderiaceae;g__Burkholderia;s__	0,99
bc04_G08;OTUwell_4	Bacteria(100.0),"Proteobacteria"(100.0),Alphaproteobacteria(97.6),Rhizobiales(97.6),Rhizobiaceae(97.6),Ensifer(93.8)	k__Bacteria;p__Proteobacteria;c__Alphaproteobacteria;o__Rhizobiales;f__Rhizobiaceae	0,94
bc04_G08;OTUwell_5	Bacteria(100.0),"Proteobacteria"(100.0),Gammaproteobacteria(99.4),Xanthomonadales(99.3),Xanthomonadaceae(99.2),Dyella(90.8)	k__Bacteria;p__Proteobacteria;c__Gammaproteobacteria;o__Xanthomonadales;f__Xanthomonadaceae;g__Dyella;s__	1,00
bc04_G08;OTUwell_6	Bacteria(100.0),"Proteobacteria"(100.0),Gammaproteobacteria(99.0),Xanthomonadales(99.0),Xanthomonadaceae(98.6),Dyella(84.7)	k__Bacteria;p__Proteobacteria;c__Gammaproteobacteria;o__Xanthomonadales;f__Xanthomonadaceae;g__Dyella;s__	1,00
bc04_G08;OTUwell_7	Bacteria(100.0),"Bacteroidetes"(100.0),"Sphingobacteria"(99.7),"Sphingobacteriales"(99.6),Chitinophagaceae(99.6),Chitinophaga(99.2)	k__Bacteria;p__Bacteroidetes;c__[Saprospirae];o__[Saprospirales];f__Chitinophagaceae;g__Chitinophaga;s__	1,00
bc04_G09;OTUwell_1	Bacteria(100.0),Firmicutes(100.0),Bacilli(99.7),Bacillales(99.5),Bacillaceae_1(99.2),Bacillus(99.2)	k__Bacteria;p__Firmicutes;c__Bacilli;o__Bacillales;f__Bacillaceae;g__Bacillus	1,00
bc04_G10;OTUwell_1	Bacteria(100.0),"Proteobacteria"(100.0),Betaproteobacteria(97.6),Burkholderiales(96.6),Burkholderiaceae(95.4),Chitinimonas(89.1)	k__Bacteria;p__Proteobacteria;c__Betaproteobacteria;o__Neisseriales;f__Neisseriaceae;g__;s__	1,00
bc04_G11;OTUwell_1	Bacteria(100.0),"Bacteroidetes"(100.0),"Sphingobacteria"(98.1),"Sphingobacteriales"(98.1),Chitinophagaceae(98.1),Filimonas(67.8)	k__Bacteria;p__Bacteroidetes;c__[Saprospirae];o__[Saprospirales];f__Chitinophagaceae;g__;s__	0,96
bc04_G12;OTUwell_1	Bacteria(100.0),"Bacteroidetes"(100.0),"Sphingobacteria"(100.0),"Sphingobacteriales"(100.0),Chitinophagaceae(100.0),Chitinophaga(99.9)	k__Bacteria;p__Bacteroidetes;c__[Saprospirae];o__[Saprospirales];f__Chitinophagaceae;g__Chitinophaga;s__	1,00
bc04_G12;OTUwell_2	Bacteria(100.0),"Proteobacteria"(100.0),Betaproteobacteria(97.3),Burkholderiales(97.3),Burkholderiales_incertae_sedis(95.9),Mitsuaria(90.8)	k__Bacteria;p__Proteobacteria;c__Betaproteobacteria;o__Burkholderiales;f__Comamonadaceae	1,00
bc04_G12;OTUwell_3	Bacteria(100.0),Firmicutes(100.0),Bacilli(99.6),Bacillales(99.4),Bacillaceae_1(99.2),Bacillus(99.2)	k__Bacteria;p__Firmicutes;c__Bacilli;o__Bacillales;f__Bacillaceae;g__Bacillus;s__flexus	1,00
bc04_H01;OTUwell_1	Bacteria(100.0),"Bacteroidetes"(100.0),"Sphingobacteria"(99.7),"Sphingobacteriales"(99.6),Chitinophagaceae(99.6),Chitinophaga(99.6)	k__Bacteria;p__Bacteroidetes;c__[Saprospirae];o__[Saprospirales];f__Chitinophagaceae;g__Chitinophaga;s__	1,00
bc04_H01;OTUwell_2	Bacteria(100.0),"Proteobacteria"(100.0),Alphaproteobacteria(99.2),Rhodospirillales(99.2),Rhodospirillaceae(99.1),Inquilinus(99.1)	k__Bacteria;p__Proteobacteria;c__Alphaproteobacteria;o__Rhodospirillales;f__Rhodospirillaceae;g__Inquilinus;s__limosus	1,00
bc04_H01;OTUwell_3	Bacteria(100.0),"Proteobacteria"(100.0),Alphaproteobacteria(97.6),Rhizobiales(97.6),Rhizobiaceae(97.5),Rhizobium(95.9)	k__Bacteria;p__Proteobacteria;c__Alphaproteobacteria;o__Rhizobiales;f__Rhizobiaceae;g__Rhizobium;s__	1,00
bc04_H02;OTUwell_1	Bacteria(100.0),"Actinobacteria"(100.0),Actinobacteria(100.0),Actinomycetales(99.7),Streptomycetaceae(99.7),Streptomyces(99.5)	k__Bacteria;p__Actinobacteria;c__Actinobacteria;o__Actinomycetales;f__Streptomycetaceae	1,00
bc04_H02;OTUwell_2	Bacteria(100.0),"Proteobacteria"(100.0),Gammaproteobacteria(99.5),Xanthomonadales(99.3),Xanthomonadaceae(99.2),Fulvimonas(90.1)	k__Bacteria;p__Proteobacteria;c__Gammaproteobacteria;o__Xanthomonadales;f__Xanthomonadaceae;g__Dyella;s__	0,97
bc04_H03;OTUwell_1	Bacteria(100.0),"Bacteroidetes"(100.0),"Sphingobacteria"(97.6),"Sphingobacteriales"(97.6),Sphingobacteriaceae(97.6),Mucilaginibacter(91.7)	k__Bacteria;p__Bacteroidetes;c__Sphingobacteriia;o__Sphingobacteriales;f__Sphingobacteriaceae;g__;s__	1,00
bc04_H03;OTUwell_2	Bacteria(100.0),"Bacteroidetes"(100.0),"Sphingobacteria"(100.0),"Sphingobacteriales"(100.0),Chitinophagaceae(100.0),Chitinophaga(99.9)	k__Bacteria;p__Bacteroidetes;c__[Saprospirae];o__[Saprospirales];f__Chitinophagaceae;g__Chitinophaga;s__	1,00
bc04_H04;OTUwell_1	Bacteria(100.0),"Proteobacteria"(100.0),Alphaproteobacteria(98.7),Rhizobiales(98.7),Rhizobiaceae(98.7),Rhizobium(98.7)	k__Bacteria;p__Proteobacteria;c__Alphaproteobacteria;o__Rhizobiales;f__Rhizobiaceae;g__Rhizobium;s__	1,00
bc04_H04;OTUwell_2	Bacteria(100.0),"Proteobacteria"(100.0),Alphaproteobacteria(98.4),Rhizobiales(98.4),Rhizobiaceae(98.4),Rhizobium(98.4)	k__Bacteria;p__Proteobacteria;c__Alphaproteobacteria;o__Rhizobiales;f__Rhizobiaceae;g__Rhizobium;s__	0,94
bc04_H04;OTUwell_3	Bacteria(100.0),"Actinobacteria"(100.0),Actinobacteria(99.6),Actinomycetales(99.5),Micrococcaceae(98.2),Arthrobacter(93.8)	k__Bacteria;p__Actinobacteria;c__Actinobacteria;o__Actinomycetales;f__Micrococcaceae;g__;s__	0,97

bc04_H04;OTUwell_4	Bacteria(100.0),"Bacteroidetes"(100.0),"Sphingobacteria"(99.8),"Sphingobacteriales"(99.6),Chitinophagaceae(99.6),Chitinophaga(99.6)	k__Bacteria;p__Bacteroidetes;c__[Saprospirae];o__[Saprospirales];f__Chitinophagaceae;g__Chitinophaga;s__	1,00
bc04_H05;OTUwell_1	Bacteria(100.0),"Proteobacteria"(100.0),Gammaproteobacteria(99.5),Xanthomonadales(99.3),Xanthomonadaceae(99.3),Fulvimonas(90.8)	k__Bacteria;p__Proteobacteria;c__Gammaproteobacteria;o__Xanthomonadales;f__Xanthomonadaceae;g__Dyella;s__	0,91
bc04_H06;OTUwell_1	Bacteria(100.0),"Bacteroidetes"(100.0),"Sphingobacteria"(100.0),"Sphingobacteriales"(100.0),Chitinophagaceae(99.8),Chitinophaga(99.6)	k__Bacteria;p__Bacteroidetes;c__[Saprospirae];o__[Saprospirales];f__Chitinophagaceae;g__Chitinophaga;s__	1,00
bc04_H07;OTUwell_1	Bacteria(100.0),Firmicutes(100.0),Bacilli(99.5),Bacillales(99.3),Planococcaceae(98.3),Sporosarcina(96.3)	k__Bacteria;p__Firmicutes;c__Bacilli;o__Bacillales;f__Planococcaceae;g__Sporosarcina;s__	0,95
bc04_H07;OTUwell_2	Bacteria(100.0),Firmicutes(100.0),Bacilli(99.6),Bacillales(99.4),Planococcaceae(98.7),Sporosarcina(98.0)	k__Bacteria;p__Firmicutes;c__Bacilli;o__Bacillales;f__Planococcaceae;g__Sporosarcina;s__	1,00
bc04_H08;OTUwell_1	Bacteria(100.0),"Bacteroidetes"(100.0),"Sphingobacteria"(99.6),"Sphingobacteriales"(99.5),Chitinophagaceae(99.5),Chitinophaga(98.7)	k__Bacteria;p__Bacteroidetes;c__[Saprospirae];o__[Saprospirales];f__Chitinophagaceae;g__Chitinophaga;s__	1,00
bc04_H08;OTUwell_2	Bacteria(100.0),Firmicutes(100.0),Bacilli(99.5),Bacillales(99.4),Bacillaceae_1(99.1),Bacillus(99.1)	k__Bacteria;p__Firmicutes;c__Bacilli;o__Bacillales;f__Bacillaceae;g__;s__	0,97
bc04_H08;OTUwell_3	Bacteria(100.0),"Bacteroidetes"(100.0),"Sphingobacteria"(100.0),"Sphingobacteriales"(100.0),Chitinophagaceae(99.5),Chitinophaga(97.7)	k__Bacteria;p__Bacteroidetes;c__[Saprospirae];o__[Saprospirales];f__Chitinophagaceae;g__Chitinophaga;s__	1,00
bc04_H09;OTUwell_1	Bacteria(100.0),"Proteobacteria"(100.0),Gammaproteobacteria(99.6),Xanthomonadales(99.4),Xanthomonadaceae(99.4),Dyella(93.8)	k__Bacteria;p__Proteobacteria;c__Gammaproteobacteria;o__Xanthomonadales;f__Xanthomonadaceae;g__Dyella;s__	1,00
bc04_H10;OTUwell_1	Bacteria(100.0),"Proteobacteria"(100.0),Gammaproteobacteria(100.0),"Enterobacteriales"(99.5),Enterobacteriaceae(99.4),Escherichia/Shigella(99.0)	k__Bacteria;p__Proteobacteria;c__Gammaproteobacteria;o__Enterobacteriales;f__Enterobacteriaceae;g__;s__	1,00
bc04_H11;OTUwell_1	Bacteria(100.0),"Proteobacteria"(100.0),Gammaproteobacteria(100.0),"Enterobacteriales"(99.5),Enterobacteriaceae(99.5),Escherichia/Shigella(99.1)	k__Bacteria;p__Proteobacteria;c__Gammaproteobacteria;o__Enterobacteriales;f__Enterobacteriaceae;g__;s__	1,00
bc04_H12;OTUwell_1	Bacteria(100.0),"Proteobacteria"(100.0),Gammaproteobacteria(100.0),"Enterobacteriales"(99.5),Enterobacteriaceae(99.5),Escherichia/Shigella(99.0)	k__Bacteria;p__Proteobacteria;c__Gammaproteobacteria;o__Enterobacteriales;f__Enterobacteriaceae;g__;s__	1,00
bc05_A01;OTUwell_1	Bacteria(100.0),"Proteobacteria"(100.0),Alphaproteobacteria(98.9),Rhizobiales(98.9),Rhizobiaceae(98.9),Rhizobium(98.9)	k__Bacteria;p__Proteobacteria;c__Alphaproteobacteria;o__Rhizobiales;f__Rhizobiaceae;g__Rhizobium;s__	1,00
bc05_A02;OTUwell_1	Bacteria(100.0),"Proteobacteria"(100.0),Alphaproteobacteria(98.9),Rhizobiales(98.9),Rhizobiaceae(98.9),Rhizobium(98.9)	k__Bacteria;p__Proteobacteria;c__Alphaproteobacteria;o__Rhizobiales;f__Rhizobiaceae;g__Rhizobium;s__	1,00
bc05_A03;OTUwell_1	Bacteria(100.0),Firmicutes(100.0),Bacilli(99.6),Bacillales(99.3),Bacillaceae_1(98.8),Bacillus(98.8)	k__Bacteria;p__Firmicutes;c__Bacilli;o__Bacillales;f__Bacillaceae;g__Bacillus	1,00
bc05_A03;OTUwell_2	Bacteria(100.0),Firmicutes(100.0),Bacilli(99.3),Bacillales(99.2),Bacillaceae_1(98.6),Bacillus(98.6)	k__Bacteria;p__Firmicutes;c__Bacilli;o__Bacillales;f__Bacillaceae;g__Bacillus	1,00
bc05_A04;OTUwell_1	Bacteria(100.0),"Proteobacteria"(100.0),Gammaproteobacteria(99.7),Xanthomonadales(99.5),Xanthomonadaceae(99.5),Dyella(97.5)	k__Bacteria;p__Proteobacteria;c__Gammaproteobacteria;o__Xanthomonadales;f__Xanthomonadaceae;g__Dyella;s__	0,99
bc05_A04;OTUwell_2	Bacteria(100.0),"Proteobacteria"(100.0),Betaproteobacteria(98.9),Burkholderiales(98.9),Comamonadaceae(97.9),Variovorax(95.0)	k__Bacteria;p__Proteobacteria;c__Betaproteobacteria;o__Burkholderiales;f__Comamonadaceae;g__Variovorax;s__paradoxus	0,86
bc05_A04;OTUwell_3	Bacteria(100.0),"Bacteroidetes"(100.0),Flavobacteria(97.9),"Flavobacteriales"(97.9),Flavobacteriaceae(97.9),Flavobacterium(92.4)	k__Bacteria;p__Bacteroidetes;c__Flavobacteriia;o__Flavobacteriales;f__Flavobacteriaceae;g__Flavobacterium;s__	1,00
bc05_A05;OTUwell_1	Bacteria(100.0),"Proteobacteria"(100.0),Gammaproteobacteria(99.6),Xanthomonadales(99.0),Xanthomonadaceae(98.6),Lysobacter(98.3)	k__Bacteria;p__Proteobacteria;c__Gammaproteobacteria;o__Xanthomonadales;f__Xanthomonadaceae;g__Lysobacter;s__	0,95
bc05_A05;OTUwell_2	Bacteria(100.0),"Actinobacteria"(100.0),Actinobacteria(100.0),Actinomycetales(99.7),Micrococcaceae(99.2),Arthrobacter(99.1)	k__Bacteria;p__Actinobacteria;c__Actinobacteria;o__Actinomycetales;f__Micrococcaceae;g__;s__	1,00
bc05_A05;OTUwell_3	Bacteria(100.0),"Proteobacteria"(100.0),Gammaproteobacteria(99.6),Xanthomonadales(98.8),Xanthomonadaceae(98.2),Lysobacter(97.1)	k__Bacteria;p__Proteobacteria;c__Gammaproteobacteria;o__Xanthomonadales;f__Xanthomonadaceae;g__Lysobacter;s__	0,94
bc05_A05;OTUwell_4	Bacteria(100.0),"Bacteroidetes"(100.0),"Sphingobacteria"(100.0),"Sphingobacteriales"(100.0),Chitinophagaceae(100.0),Chitinophaga(99.9)	k__Bacteria;p__Bacteroidetes;c__[Saprospirae];o__[Saprospirales];f__Chitinophagaceae;g__Chitinophaga;s__	1,00
bc05_A06;OTUwell_1	Bacteria(100.0),"Proteobacteria"(100.0),Gammaproteobacteria(99.8),Xanthomonadales(99.2),Xanthomonadaceae(98.9),Lysobacter(98.9)	k__Bacteria;p__Proteobacteria;c__Gammaproteobacteria;o__Xanthomonadales;f__Xanthomonadaceae;g__Lysobacter;s__	1,00
bc05_A06;OTUwell_2	Bacteria(100.0),"Proteobacteria"(100.0),Betaproteobacteria(99.2),Burkholderiales(99.2),Comamonadaceae(98.2),Variovorax(95.4)	k__Bacteria;p__Proteobacteria;c__Betaproteobacteria;o__Burkholderiales;f__Comamonadaceae;g__Variovorax;s__paradoxus	0,97
bc05_A07;OTUwell_1	Bacteria(100.0),"Proteobacteria"(100.0),Alphaproteobacteria(98.6),Rhizobiales(98.6),Rhizobiaceae(98.6),Rhizobium(98.6)	k__Bacteria;p__Proteobacteria;c__Alphaproteobacteria;o__Rhizobiales;f__Rhizobiaceae;g__Rhizobium;s__	1,00
bc05_A07;OTUwell_2	Bacteria(100.0),"Proteobacteria"(100.0),Alphaproteobacteria(97.8),Rhizobiales(97.8),Rhizobiaceae(97.7),Rhizobium(96.3)	k__Bacteria;p__Proteobacteria;c__Alphaproteobacteria;o__Rhizobiales;f__Rhizobiaceae;g__Rhizobium;s__	0,90
bc05_A08;OTUwell_1	Bacteria(100.0),"Proteobacteria"(100.0),Alphaproteobacteria(98.9),Rhizobiales(98.9),Rhizobiaceae(98.9),Rhizobium(98.9)	k__Bacteria;p__Proteobacteria;c__Alphaproteobacteria;o__Rhizobiales;f__Rhizobiaceae;g__Rhizobium;s__	1,00
bc05_A08;OTUwell_2	Bacteria(100.0),"Proteobacteria"(100.0),Betaproteobacteria(99.1),Burkholderiales(99.1),Burkholderiaceae(99.1),Burkholderia(98.8)	k__Bacteria;p__Proteobacteria;c__Betaproteobacteria;o__Burkholderiales;f__Burkholderiaceae;g__Burkholderia;s__	1,00
bc05_A11;OTUwell_1	Bacteria(100.0),"Bacteroidetes"(100.0),"Sphingobacteria"(100.0),"Sphingobacteriales"(100.0),Chitinophagaceae(100.0),Chitinophaga(99.7)	k__Bacteria;p__Bacteroidetes;c__[Saprospirae];o__[Saprospirales];f__Chitinophagaceae;g__Chitinophaga;s__	1,00
bc05_A12;OTUwell_1	Bacteria(100.0),"Bacteroidetes"(100.0),"Sphingobacteria"(100.0),"Sphingobacteriales"(100.0),Cytophagaceae(99.5),Dyadobacter(99.5)	k__Bacteria;p__Bacteroidetes;c__Cytophagia;o__Cytophagales;f__Cytophagaceae;g__Dyadobacter;s__	1,00
bc05_A12;OTUwell_2	Bacteria(100.0),"Bacteroidetes"(100.0),"Sphingobacteria"(99.1),"Sphingobacteriales"(99.1),Sphingobacteriaceae(99.1),Mucilaginibacter(99.1)	k__Bacteria;p__Bacteroidetes;c__Sphingobacteriia;o__Sphingobacteriales;f__Sphingobacteriaceae;g__;s__	1,00
bc05_B01;OTUwell_1	Bacteria(100.0),"Proteobacteria"(100.0),Alphaproteobacteria(98.9),Rhizobiales(98.9),Rhizobiaceae(98.9),Rhizobium(98.9)	k__Bacteria;p__Proteobacteria;c__Alphaproteobacteria;o__Rhizobiales;f__Rhizobiaceae;g__Rhizobium;s__	1,00
bc05_B02;OTUwell_1	Bacteria(100.0),"Proteobacteria"(100.0),Betaproteobacteria(99.0),Burkholderiales(99.0),Burkholderiaceae(98.7),Burkholderia(97.5)	k__Bacteria;p__Proteobacteria;c__Betaproteobacteria;o__Burkholderiales;f__Burkholderiaceae;g__Burkholderia;s__	0,89
bc05_B02;OTUwell_2	Bacteria(100.0),"Proteobacteria"(100.0),Alphaproteobacteria(98.9),Rhizobiales(98.9),Rhizobiaceae(98.9),Rhizobium(98.9)	k__Bacteria;p__Proteobacteria;c__Alphaproteobacteria;o__Rhizobiales;f__Rhizobiaceae;g__Rhizobium;s__	1,00
bc05_B03;OTUwell_1	Bacteria(100.0),"Actinobacteria"(100.0),Actinobacteria(99.6),Actinomycetales(99.5),Micrococcaceae(98.3),Arthrobacter(94.5)	k__Bacteria;p__Actinobacteria;c__Actinobacteria;o__Actinomycetales;f__Micrococcaceae;g__;s__	1,00
bc05_B04;OTUwell_1	Bacteria(100.0),"Proteobacteria"(100.0),Alphaproteobacteria(98.2),Rhizobiales(98.2),Rhizobiaceae(98.2),Rhizobium(98.0)	k__Bacteria;p__Proteobacteria;c__Alphaproteobacteria;o__Rhizobiales;f__Rhizobiaceae;g__Rhizobium;s__	0,86
bc05_B04;OTUwell_2	Bacteria(100.0),"Proteobacteria"(100.0),Alphaproteobacteria(98.4),Rhizobiales(98.4),Rhizobiaceae(98.4),Rhizobium(98.4)	k__Bacteria;p__Proteobacteria;c__Alphaproteobacteria;o__Rhizobiales;f__Rhizobiaceae;g__Rhizobium;s__	1,00
bc05_B05;OTUwell_1	Bacteria(100.0),"Proteobacteria"(100.0),Alphaproteobacteria(98.9),Rhodospirillales(98.9),Rhodospirillaceae(98.8),Inquilinus(98.8)	k__Bacteria;p__Proteobacteria;c__Alphaproteobacteria;o__Rhodospirillales;f__Rhodospirillaceae;g__Inquilinus;s__limosus	1,00
bc05_B05;OTUwell_2	Bacteria(100.0),"Proteobacteria"(100.0),Alphaproteobacteria(96.8),Rhodospirillales(96.8),Rhodospirillaceae(96.2),Inquilinus(91.7)	k__Bacteria;p__Proteobacteria;c__Alphaproteobacteria;o__Rhodospirillales;f__Rhodospirillaceae;g__Inquilinus;s__limosus	1,00
bc05_B06;OTUwell_1	Bacteria(100.0),"Actinobacteria"(100.0),Actinobacteria(100.0),Actinomycetales(99.7),Mycobacteriaceae(99.3),Mycobacterium(99.3)	k__Bacteria;p__Actinobacteria;c__Actinobacteria;o__Actinomycetales;f__Mycobacteriaceae;g__Mycobacterium;s__	1,00
bc05_B06;OTUwell_2	Bacteria(100.0),"Bacteroidetes"(100.0),"Sphingobacteria"(99.6),"Sphingobacteriales"(99.5),Chitinophagaceae(99.5),Chitinophaga(98.9)	k__Bacteria;p__Bacteroidetes;c__[Saprospirae];o__[Saprospirales];f__Chitinophagaceae;g__Chitinophaga;s__	1,00
bc05_B07;OTUwell_1	Bacteria(100.0),"Proteobacteria"(100.0),Gammaproteobacteria(99.7),Xanthomonadales(99.5),Xanthomonadaceae(99.5),Dyella(97.7)	k__Bacteria;p__Proteobacteria;c__Gammaproteobacteria;o__Xanthomonadales;f__Xanthomonadaceae;g__Dyella;s__	0,99
bc05_B08;OTUwell_1	Bacteria(100.0),Firmicutes(100.0),Bacilli(99.7),Bacillales(99.5),Bacillaceae_1(99.4),Bacillus(99.4)	k__Bacteria;p__Firmicutes;c__Bacilli;o__Bacillales;f__Bacillaceae;g__;s__	0,98
bc05_B08;OTUwell_2	Bacteria(100.0),"Proteobacteria"(100.0),Gammaproteobacteria(99.6),Xanthomonadales(99.4),Xanthomonadaceae(99.4),Dyella(95.0)	k__Bacteria;p__Proteobacteria;c__Gammaproteobacteria;o__Xanthomonadales;f__Xanthomonadaceae;g__Dyella;s__	1,00
bc05_B08;OTUwell_3	Bacteria(100.0),"Proteobacteria"(100.0),Gammaproteobacteria(99.2),Xanthomonadales(99.1),Xanthomonadaceae(98.8),Dyella(86.9)	k__Bacteria;p__Proteobacteria;c__Gammaproteobacteria;o__Xanthomonadales;f__Xanthomonadaceae;g__Dyella;s__	1,00
bc05_B09;OTUwell_1	Bacteria(100.0),"Proteobacteria"(100.0),Alphaproteobacteria(98.9),Rhizobiales(98.9),Rhizobiaceae(98.9),Rhizobium(98.9)	k__Bacteria;p__Proteobacteria;c__Alphaproteobacteria;o__Rhizobiales;f__Rhizobiaceae;g__Rhizobium;s__	1,00
bc05_B10;OTUwell_1	Bacteria(100.0),"Proteobacteria"(100.0),Betaproteobacteria(97.4),Burkholderiales(96.2),Burkholderiaceae(95.2),Chitinimonas(86.9)	k__Bacteria;p__Proteobacteria;c__Betaproteobacteria;o__Neisseriales;f__Neisseriaceae;g__;s__	1,00
bc05_C02;OTUwell_1	Bacteria(100.0),"Proteobacteria"(100.0),Alphaproteobacteria(98.9),Rhizobiales(98.9),Rhizobiaceae(98.9),Rhizobium(98.9)	k__Bacteria;p__Proteobacteria;c__Alphaproteobacteria;o__Rhizobiales;f__Rhizobiaceae;g__Rhizobium;s__	1,00
bc05_C02;OTUwell_2	Bacteria(100.0),Firmicutes(100.0),Bacilli(99.7),Bacillales(99.5),Bacillaceae_1(99.4),Bacillus(99.4)	k__Bacteria;p__Firmicutes;c__Bacilli;o__Bacillales;f__Bacillaceae;g__;s__	0,99
bc05_C03;OTUwell_1	Bacteria(100.0),"Proteobacteria"(100.0),Betaproteobacteria(99.3),Burkholderiales(99.3),Burkholderiaceae(99.2),Burkholderia(98.9)	k__Bacteria;p__Proteobacteria;c__Betaproteobacteria;o__Burkholderiales;f__Burkholderiaceae;g__Salinispora;s__	0,99
bc05_C03;OTUwell_2	Bacteria(100.0),"Proteobacteria"(100.0),Gammaproteobacteria(99.7),Xanthomonadales(99.5),Xanthomonadaceae(99.5),Dyella(97.1)	k__Bacteria;p__Proteobacteria;c__Gammaproteobacteria;o__Xanthomonadales;f__Xanthomonadaceae;g__Dyella;s__	1,00
bc05_C03;OTUwell_3	Bacteria(100.0),"Bacteroidetes"(100.0),"Sphingobacteria"(98.1),"Sphingobacteriales"(98.1),Sphingobacteriaceae(98.1),Pedobacter(96.3)	k__Bacteria;p__Bacteroidetes;c__Sphingobacteriia;o__Sphingobacteriales;f__Sphingobacteriaceae;g__Pedobacter;s__	1,00
bc05_C04;OTUwell_1	Bacteria(100.0),"Proteobacteria"(100.0),Gammaproteobacteria(99.6),Xanthomonadales(99.5),Xanthomonadaceae(99.4),Dyella(94.5)	k__Bacteria;p__Proteobacteria;c__Gammaproteobacteria;o__Xanthomonadales;f__Xanthomonadaceae;g__Dyella;s__	1,00
bc05_C04;OTUwell_2	Bacteria(100.0),"Proteobacteria"(100.0),Alphaproteobacteria(98.4),Rhizobiales(98.4),Rhizobiaceae(98.4),Rhizobium(98.4)	k__Bacteria;p__Proteobacteria;c__Alphaproteobacteria;o__Rhizobiales;f__Rhizobiaceae;g__Rhizobium;s__	0,98
bc05_C07;OTUwell_1	Bacteria(100.0),"Proteobacteria"(100.0),Betaproteobacteria(99.0),Burkholderiales(99.0),Burkholderiaceae(98.8),Wautersia(90.1)	k__Bacteria;p__Proteobacteria;c__Betaproteobacteria;o__Burkholderiales;f__Oxalobacteraceae;g__Cupriavidus;s__	1,00
bc05_C08;OTUwell_1	Bacteria(100.0),"Proteobacteria"(100.0),Betaproteobacteria(98.9),Burkholderiales(98.9),Comamonadaceae(97.4),Variovorax(94.5)	k__Bacteria;p__Proteobacteria;c__Betaproteobacteria;o__Burkholderiales;f__Comamonadaceae	1,00
bc05_C09;OTUwell_1	Bacteria(100.0),"Bacteroidetes"(100.0),"Sphingobacteria"(98.6),"Sphingobacteriales"(98.6),Sphingobacteriaceae(98.6),Pedobacter(98.6)	k__Bacteria;p__Bacteroidetes;c__Sphingobacteriia;o__Sphingobacteriales;f__Sphingobacteriaceae;g__Pedobacter;s__	1,00
bc05_C09;OTUwell_2	Bacteria(100.0),"Proteobacteria"(100.0),Alphaproteobacteria(99.6),Caulobacterales(99.6),Caulobacteraceae(99.6),Asticcacaulis(99.4)	k__Bacteria;p__Proteobacteria;c__Alphaproteobacteria;o__Caulobacterales;f__Caulobacteraceae;g__Asticcacaulis;s__biprosthecium	1,00
bc05_C10;OTUwell_1	Bacteria(100.0),"Proteobacteria"(100.0),Gammaproteobacteria(99.3),Xanthomonadales(99.3),Sinobacteraceae(99.3),Sinobacter(83.4)	k__Bacteria;p__Proteobacteria;c__Gammaproteobacteria;o__Xanthomonadales;f__Sinobacteraceae;g__;s__	1,00
bc05_C12;OTUwell_1	Bacteria(100.0),"Proteobacteria"(100.0),Gammaproteobacteria(99.7),Xanthomonadales(99.5),Xanthomonadaceae(99.5),Dyella(98.0)	k__Bacteria;p__Proteobacteria;c__Gammaproteobacteria;o__Xanthomonadales;f__Xanthomonadaceae;g__Dyella;s__	0,98
bc05_D01;OTUwell_1	Bacteria(100.0),"Proteobacteria"(100.0),Gammaproteobacteria(100.0),"Enterobacteriales"(98.8),Enterobacteriaceae(98.2),Enterobacter(89.1)	k__Bacteria;p__Proteobacteria;c__Gammaproteobacteria;o__Enterobacteriales;f__Enterobacteriaceae;g__;s__	0,97
bc05_D01;OTUwell_2	Bacteria(100.0),"Proteobacteria"(100.0),Gammaproteobacteria(100.0),"Enterobacteriales"(99.1),Enterobacteriaceae(98.7),Enterobacter(95.0)	k__Bacteria;p__Proteobacteria;c__Gammaproteobacteria;o__Enterobacteriales;f__Enterobacteriaceae;g__;s__	0,86
bc05_D02;OTUwell_1	Bacteria(100.0),"Proteobacteria"(100.0),Alphaproteobacteria(98.8),Rhizobiales(98.8),Rhizobiaceae(98.8),Rhizobium(98.8)	k__Bacteria;p__Proteobacteria;c__Alphaproteobacteria;o__Rhizobiales;f__Rhizobiaceae;g__Rhizobium;s__	1,00
bc05_D03;OTUwell_1	Bacteria(100.0),Firmicutes(100.0),Bacilli(99.7),Bacillales(99.5),Bacillaceae_1(99.3),Bacillus(99.3)	k__Bacteria;p__Firmicutes;c__Bacilli;o__Bacillales;f__Bacillaceae;g__Bacillus	1,00
bc05_D03;OTUwell_2	Bacteria(100.0),"Proteobacteria"(100.0),Alphaproteobacteria(98.5),Sphingomonadales(98.1),Sphingomonadaceae(96.4),Novosphingobium(82.2)	k__Bacteria;p__Proteobacteria;c__Alphaproteobacteria;o__Sphingomonadales;f__Sphingomonadaceae;g__Novosphingobium;s__	0,97
bc05_D04;OTUwell_1	Bacteria(100.0),"Bacteroidetes"(100.0),"Sphingobacteria"(98.9),"Sphingobacteriales"(98.9),Chitinophagaceae(98.9),Niastella(79.9)	k__Bacteria;p__Bacteroidetes;c__[Saprospirae];o__[Saprospirales];f__Chitinophagaceae;g__;s__	1,00
bc05_D05;OTUwell_1	Bacteria(100.0),"Proteobacteria"(100.0),Gammaproteobacteria(99.7),Xanthomonadales(99.5),Xanthomonadaceae(99.5),Dyella(97.5)	k__Bacteria;p__Proteobacteria;c__Gammaproteobacteria;o__Xanthomonadales;f__Xanthomonadaceae;g__Dyella;s__	0,99
bc05_D05;OTUwell_2	Bacteria(100.0),"Proteobacteria"(100.0),Betaproteobacteria(98.6),Burkholderiales(98.6),Burkholderiaceae(98.5),Wautersia(84.7)	k__Bacteria;p__Proteobacteria;c__Betaproteobacteria;o__Burkholderiales;f__Oxalobacteraceae;g__Cupriavidus;s__	1,00
bc05_D06;OTUwell_1	Bacteria(100.0),"Proteobacteria"(100.0),Gammaproteobacteria(96.8),Pseudomonadales(96.8),Pseudomonadaceae(96.4),Rhizobacter(92.4)	k__Bacteria;p__Proteobacteria;c__Betaproteobacteria;o__Burkholderiales;f__Comamonadaceae;g__Methylibium;s__	1,00
bc05_D07;OTUwell_1	Bacteria(100.0),"Proteobacteria"(100.0),Gammaproteobacteria(99.5),Xanthomonadales(99.3),Xanthomonadaceae(99.3),Fulvimonas(90.8)	k__Bacteria;p__Proteobacteria;c__Gammaproteobacteria;o__Xanthomonadales;f__Xanthomonadaceae;g__Dyella;s__	0,94
bc05_D07;OTUwell_2	Bacteria(100.0),"Proteobacteria"(100.0),Gammaproteobacteria(99.6),Xanthomonadales(99.4),Xanthomonadaceae(99.4),Dyella(93.2)	k__Bacteria;p__Proteobacteria;c__Gammaproteobacteria;o__Xanthomonadales;f__Xanthomonadaceae;g__Dyella;s__	1,00
bc05_D07;OTUwell_3	Bacteria(100.0),"Proteobacteria"(100.0),Gammaproteobacteria(100.0),"Enterobacteriales"(99.2),Enterobacteriaceae(98.9),Enterobacter(95.4)	k__Bacteria;p__Proteobacteria;c__Gammaproteobacteria;o__Enterobacteriales;f__Enterobacteriaceae	1,00
bc05_D08;OTUwell_1	Bacteria(100.0),"Proteobacteria"(100.0),Alphaproteobacteria(98.7),Rhizobiales(98.7),Rhizobiaceae(98.7),Rhizobium(98.7)	k__Bacteria;p__Proteobacteria;c__Alphaproteobacteria;o__Rhizobiales;f__Rhizobiaceae;g__Rhizobium;s__	0,97

bc05_D08;OTUwell_2	Bacteria(100.0),Firmicutes(100.0),Bacilli(99.5),Bacillales(99.5),Bacillaceae_1(99.3),Bacillus(99.3)	k__Bacteria;p__Firmicutes;c__Bacilli;o__Bacillales;f__Bacillaceae;g__;s__	0,96
bc05_D09;OTUwell_1	Bacteria(100.0),"Proteobacteria"(100.0),Betaproteobacteria(97.6),Burkholderiales(96.6),Burkholderiaceae(95.4),Chitinimonas(89.1)	k__Bacteria;p__Proteobacteria;c__Betaproteobacteria;o__Neisseriales;f__Neisseriaceae;g__;s__	1,00
bc05_D11;OTUwell_1	Bacteria(100.0),"Proteobacteria"(100.0),Betaproteobacteria(96.6),Burkholderiales(96.6),Oxalobacteraceae(96.2),Herbaspirillum(85.8)	k__Bacteria;p__Proteobacteria;c__Betaproteobacteria;o__Burkholderiales;f__Oxalobacteraceae	1,00
bc05_D11;OTUwell_2	Bacteria(100.0),"Proteobacteria"(100.0),Betaproteobacteria(97.2),Burkholderiales(97.2),Oxalobacteraceae(97.2),Herbaspirillum(90.1)	k__Bacteria;p__Proteobacteria;c__Betaproteobacteria;o__Burkholderiales;f__Oxalobacteraceae	0,96
bc05_D11;OTUwell_3	Bacteria(100.0),"Proteobacteria"(100.0),Alphaproteobacteria(99.6),Caulobacterales(99.6),Caulobacteraceae(99.6),Caulobacter(99.2)	k__Bacteria;p__Proteobacteria;c__Alphaproteobacteria;o__Caulobacterales;f__Caulobacteraceae;g__Caulobacter;s__	1,00
bc05_D11;OTUwell_4	Bacteria(100.0),"Proteobacteria"(100.0),Betaproteobacteria(96.0),Burkholderiales(96.0),Oxalobacteraceae(95.2),Herbaspirillum(81.1)	k__Bacteria;p__Proteobacteria;c__Betaproteobacteria;o__Burkholderiales;f__Oxalobacteraceae	0,99
bc05_E02;OTUwell_1	Bacteria(100.0),"Bacteroidetes"(100.0),"Sphingobacteria"(100.0),"Sphingobacteriales"(100.0),Chitinophagaceae(100.0),Chitinophaga(99.9)	k__Bacteria;p__Bacteroidetes;c__[Saprospirae];o__[Saprospirales];f__Chitinophagaceae;g__Chitinophaga;s__	1,00
bc05_E04;OTUwell_1	Bacteria(100.0),"Proteobacteria"(100.0),Alphaproteobacteria(98.4),Rhizobiales(98.4),Rhizobiaceae(98.4),Rhizobium(98.4)	k__Bacteria;p__Proteobacteria;c__Alphaproteobacteria;o__Rhizobiales;f__Rhizobiaceae;g__Rhizobium;s__	0,96
bc05_E04;OTUwell_2	Bacteria(100.0),"Proteobacteria"(100.0),Betaproteobacteria(98.9),Burkholderiales(98.9),Comamonadaceae(97.7),Variovorax(93.2)	k__Bacteria;p__Proteobacteria;c__Betaproteobacteria;o__Burkholderiales;f__Comamonadaceae;g__Variovorax;s__paradoxus	0,97
bc05_E04;OTUwell_3	Bacteria(100.0),"Proteobacteria"(100.0),Betaproteobacteria(98.4),Burkholderiales(98.4),Comamonadaceae(96.2),Variovorax(88.0)	k__Bacteria;p__Proteobacteria;c__Betaproteobacteria;o__Burkholderiales;f__Comamonadaceae;g__Variovorax	0,94
bc05_E05;OTUwell_1	Bacteria(100.0),"Proteobacteria"(100.0),Gammaproteobacteria(99.8),Xanthomonadales(99.5),Xanthomonadaceae(99.5),Dokdonella(99.5)	k__Bacteria;p__Proteobacteria;c__Gammaproteobacteria;o__Xanthomonadales;f__Xanthomonadaceae;g__Dokdonella;s__	1,00
bc05_E06;OTUwell_1	Bacteria(100.0),"Bacteroidetes"(100.0),"Sphingobacteria"(98.9),"Sphingobacteriales"(98.9),Chitinophagaceae(98.9),Terrimonas(84.7)	k__Bacteria;p__Bacteroidetes;c__[Saprospirae];o__[Saprospirales];f__Chitinophagaceae;g__;s__	0,98
bc05_E08;OTUwell_1	Bacteria(100.0),"Bacteroidetes"(100.0),"Sphingobacteria"(100.0),"Sphingobacteriales"(100.0),Chitinophagaceae(100.0),Chitinophaga(99.7)	k__Bacteria;p__Bacteroidetes;c__[Saprospirae];o__[Saprospirales];f__Chitinophagaceae;g__Chitinophaga;s__	1,00
bc05_E09;OTUwell_1	Bacteria(100.0),"Proteobacteria"(100.0),Betaproteobacteria(96.6),Burkholderiales(94.0),Burkholderiaceae(92.4),Chitinimonas(78.6)	k__Bacteria;p__Proteobacteria;c__Betaproteobacteria;o__Neisseriales;f__Neisseriaceae;g__;s__	1,00
bc05_E10;OTUwell_1	Bacteria(100.0),"Proteobacteria"(100.0),Alphaproteobacteria(99.6),Sphingomonadales(99.3),Sphingomonadaceae(99.2),Sphingobium(97.7)	k__Bacteria;p__Proteobacteria;c__Alphaproteobacteria;o__Sphingomonadales;f__Sphingomonadaceae;g__Sphingobium;s__	1,00
bc05_E11;OTUwell_1	Bacteria(100.0),"Proteobacteria"(100.0),Betaproteobacteria(98.9),Burkholderiales(98.9),Comamonadaceae(97.8),Variovorax(95.9)	k__Bacteria;p__Proteobacteria;c__Betaproteobacteria;o__Burkholderiales;f__Comamonadaceae;g__Variovorax;s__paradoxus	0,84
bc05_E12;OTUwell_1	Bacteria(100.0),"Proteobacteria"(100.0),Alphaproteobacteria(97.1),Sphingomonadales(91.9),Sphingomonadaceae(90.2),Sphingomonas(72.5)	k__Bacteria;p__Proteobacteria;c__Alphaproteobacteria;o__Sphingomonadales;f__Sphingomonadaceae;g__Sphingomonas;s__wittichii	0,93
bc05_E12;OTUwell_2	Bacteria(100.0),"Proteobacteria"(100.0),Alphaproteobacteria(97.9),Sphingomonadales(94.0),Sphingomonadaceae(92.4),Sphingomonas(79.9)	k__Bacteria;p__Proteobacteria;c__Alphaproteobacteria;o__Sphingomonadales;f__Sphingomonadaceae;g__Sphingomonas;s__wittichii	1,00
bc05_F01;OTUwell_1	Bacteria(100.0),"Proteobacteria"(100.0),Betaproteobacteria(98.6),Burkholderiales(98.6),Burkholderiaceae(98.2),Burkholderia(93.8)	k__Bacteria;p__Proteobacteria;c__Betaproteobacteria;o__Burkholderiales;f__Burkholderiaceae;g__Burkholderia;s__	0,88
bc05_F01;OTUwell_2	Bacteria(100.0),"Proteobacteria"(100.0),Gammaproteobacteria(96.4),Pseudomonadales(96.4),Pseudomonadaceae(95.9),Rhizobacter(90.1)	k__Bacteria;p__Proteobacteria;c__Betaproteobacteria;o__Burkholderiales;f__Comamonadaceae;g__Methylibium;s__	1,00
bc05_F01;OTUwell_3	Bacteria(100.0),"Proteobacteria"(100.0),Betaproteobacteria(98.7),Burkholderiales(98.7),Burkholderiaceae(98.5),Burkholderia(94.5)	k__Bacteria;p__Proteobacteria;c__Betaproteobacteria;o__Burkholderiales;f__Burkholderiaceae	1,00
bc05_F01;OTUwell_4	Bacteria(100.0),Firmicutes(100.0),Bacilli(99.7),Bacillales(99.5),Bacillaceae_1(99.4),Bacillus(99.4)	k__Bacteria;p__Firmicutes;c__Bacilli;o__Bacillales;f__Bacillaceae;g__;s__	0,98
bc05_F02;OTUwell_1	Bacteria(100.0),"Proteobacteria"(100.0),Alphaproteobacteria(99.7),Caulobacterales(99.6),Caulobacteraceae(99.6),Asticcacaulis(99.6)	k__Bacteria;p__Proteobacteria;c__Alphaproteobacteria;o__Caulobacterales;f__Caulobacteraceae;g__Asticcacaulis;s__biprosthecium	1,00
bc05_F03;OTUwell_1	Bacteria(100.0),"Proteobacteria"(100.0),Betaproteobacteria(97.6),Burkholderiales(96.6),Burkholderiaceae(95.4),Chitinimonas(89.1)	k__Bacteria;p__Proteobacteria;c__Betaproteobacteria;o__Neisseriales;f__Neisseriaceae;g__;s__	1,00
bc05_F03;OTUwell_2	Bacteria(100.0),"Proteobacteria"(100.0),Betaproteobacteria(98.9),Burkholderiales(98.9),Burkholderiaceae(98.7),Burkholderia(97.7)	k__Bacteria;p__Proteobacteria;c__Betaproteobacteria;o__Burkholderiales;f__Burkholderiaceae;g__Burkholderia;s__	0,90
bc05_F05;OTUwell_1	Bacteria(100.0),"Proteobacteria"(100.0),Gammaproteobacteria(96.5),Pseudomonadales(96.5),Pseudomonadaceae(96.2),Rhizobacter(90.8)	k__Bacteria;p__Proteobacteria;c__Betaproteobacteria;o__Burkholderiales;f__Comamonadaceae;g__Methylibium;s__	1,00
bc05_F06;OTUwell_1	Bacteria(100.0),"Proteobacteria"(100.0),Gammaproteobacteria(96.8),Pseudomonadales(96.8),Pseudomonadaceae(96.4),Rhizobacter(92.4)	k__Bacteria;p__Proteobacteria;c__Betaproteobacteria;o__Burkholderiales;f__Comamonadaceae;g__Methylibium;s__	1,00
bc05_F09;OTUwell_1	Bacteria(100.0),"Proteobacteria"(100.0),Alphaproteobacteria(98.9),Rhizobiales(98.9),Rhizobiaceae(98.9),Rhizobium(98.9)	k__Bacteria;p__Proteobacteria;c__Alphaproteobacteria;o__Rhizobiales;f__Rhizobiaceae;g__Rhizobium;s__	1,00
bc05_F09;OTUwell_2	Bacteria(100.0),"Proteobacteria"(100.0),Betaproteobacteria(98.7),Burkholderiales(98.7),Comamonadaceae(97.4),Variovorax(92.4)	k__Bacteria;p__Proteobacteria;c__Betaproteobacteria;o__Burkholderiales;f__Comamonadaceae;g__Variovorax;s__paradoxus	0,93
bc05_F10;OTUwell_1	Bacteria(100.0),"Proteobacteria"(100.0),Gammaproteobacteria(96.6),Pseudomonadales(96.6),Pseudomonadaceae(96.4),Rhizobacter(92.4)	k__Bacteria;p__Proteobacteria;c__Betaproteobacteria;o__Burkholderiales;f__Comamonadaceae;g__Methylibium;s__	1,00
bc05_F11;OTUwell_1	Bacteria(100.0),"Bacteroidetes"(100.0),"Sphingobacteria"(99.9),"Sphingobacteriales"(99.9),Chitinophagaceae(99.9),Chitinophaga(99.9)	k__Bacteria;p__Bacteroidetes;c__[Saprospirae];o__[Saprospirales];f__Chitinophagaceae;g__Chitinophaga;s__	1,00
bc05_F11;OTUwell_2	Bacteria(100.0),"Bacteroidetes"(100.0),"Sphingobacteria"(99.6),"Sphingobacteriales"(99.6),Chitinophagaceae(99.6),Chitinophaga(99.4)	k__Bacteria;p__Bacteroidetes;c__[Saprospirae];o__[Saprospirales];f__Chitinophagaceae;g__Chitinophaga;s__	1,00
bc05_F12;OTUwell_1	Bacteria(100.0),"Bacteroidetes"(100.0),"Sphingobacteria"(99.9),"Sphingobacteriales"(99.9),Chitinophagaceae(99.9),Chitinophaga(99.9)	k__Bacteria;p__Bacteroidetes;c__[Saprospirae];o__[Saprospirales];f__Chitinophagaceae;g__Chitinophaga;s__	1,00
bc05_F12;OTUwell_2	Bacteria(100.0),"Proteobacteria"(100.0),Gammaproteobacteria(96.5),Pseudomonadales(96.5),Pseudomonadaceae(96.2),Rhizobacter(91.7)	k__Bacteria;p__Proteobacteria;c__Betaproteobacteria;o__Burkholderiales;f__Comamonadaceae;g__Methylibium;s__	1,00
bc05_G01;OTUwell_1	Bacteria(100.0),"Proteobacteria"(100.0),Gammaproteobacteria(99.3),Xanthomonadales(99.2),Xanthomonadaceae(99.1),Fulvimonas(88.0)	k__Bacteria;p__Proteobacteria;c__Gammaproteobacteria;o__Xanthomonadales;f__Xanthomonadaceae;g__Dyella;s__	0,97
bc05_G02;OTUwell_1	Bacteria(100.0),"Proteobacteria"(100.0),Gammaproteobacteria(100.0),"Enterobacteriales"(98.8),Enterobacteriaceae(98.0),Enterobacter(90.1)	k__Bacteria;p__Proteobacteria;c__Gammaproteobacteria;o__Enterobacteriales;f__Enterobacteriaceae;g__;s__	0,92
bc05_G02;OTUwell_2	Bacteria(100.0),"Proteobacteria"(100.0),Gammaproteobacteria(100.0),"Enterobacteriales"(99.0),Enterobacteriaceae(98.6),Enterobacter(93.2)	k__Bacteria;p__Proteobacteria;c__Gammaproteobacteria;o__Enterobacteriales;f__Enterobacteriaceae;g__;s__	0,94
bc05_G03;OTUwell_1	Bacteria(100.0),Firmicutes(100.0),Bacilli(99.6),Bacillales(99.4),Bacillaceae_1(98.8),Bacillus(98.8)	k__Bacteria;p__Firmicutes;c__Bacilli;o__Bacillales;f__Bacillaceae;g__Bacillus	1,00
bc05_G04;OTUwell_1	Bacteria(100.0),"Bacteroidetes"(100.0),"Sphingobacteria"(99.6),"Sphingobacteriales"(99.6),Chitinophagaceae(99.6),Chitinophaga(99.0)	k__Bacteria;p__Bacteroidetes;c__[Saprospirae];o__[Saprospirales];f__Chitinophagaceae;g__Chitinophaga;s__	1,00
bc05_G05;OTUwell_1	Bacteria(100.0),"Bacteroidetes"(100.0),"Sphingobacteria"(100.0),"Sphingobacteriales"(100.0),Chitinophagaceae(100.0),Chitinophaga(99.6)	k__Bacteria;p__Bacteroidetes;c__[Saprospirae];o__[Saprospirales];f__Chitinophagaceae;g__Chitinophaga;s__	1,00
bc05_G06;OTUwell_1	Bacteria(100.0),"Proteobacteria"(100.0),Betaproteobacteria(99.5),Burkholderiales(99.5),Burkholderiaceae(99.5),Burkholderia(99.4)	k__Bacteria;p__Proteobacteria;c__Betaproteobacteria;o__Burkholderiales;f__Burkholderiaceae;g__Burkholderia;s__	1,00
bc05_G06;OTUwell_2	Bacteria(100.0),"Proteobacteria"(100.0),Alphaproteobacteria(99.2),Rhizobiales(99.2),Bradyrhizobiaceae(99.0),Bosea(99.0)	k__Bacteria;p__Proteobacteria;c__Alphaproteobacteria;o__Rhizobiales	1,00
bc05_G06;OTUwell_3	Bacteria(100.0),"Proteobacteria"(100.0),Alphaproteobacteria(99.4),Rhizobiales(99.4),Bradyrhizobiaceae(99.3),Bosea(99.3)	k__Bacteria;p__Proteobacteria;c__Alphaproteobacteria;o__Rhizobiales	1,00
bc05_G06;OTUwell_4	Bacteria(100.0),"Proteobacteria"(100.0),Alphaproteobacteria(99.0),Rhizobiales(99.0),Bradyrhizobiaceae(98.8),Bosea(98.8)	k__Bacteria;p__Proteobacteria;c__Alphaproteobacteria;o__Rhizobiales	1,00
bc05_G07;OTUwell_1	Bacteria(100.0),"Actinobacteria"(100.0),Actinobacteria(100.0),Actinomycetales(100.0),Microbacteriaceae(99.4),Microbacterium(98.3)	k__Bacteria;p__Actinobacteria;c__Actinobacteria;o__Actinomycetales;f__Microbacteriaceae;g__Microbacterium	1,00
bc05_G07;OTUwell_2	Bacteria(100.0),"Proteobacteria"(100.0),Gammaproteobacteria(99.6),Xanthomonadales(99.4),Xanthomonadaceae(99.3),Dyella(93.2)	k__Bacteria;p__Proteobacteria;c__Gammaproteobacteria;o__Xanthomonadales;f__Xanthomonadaceae;g__Dyella;s__	1,00
bc05_G07;OTUwell_3	Bacteria(100.0),"Actinobacteria"(100.0),Actinobacteria(100.0),Actinomycetales(99.6),Microbacteriaceae(99.4),Microbacterium(98.9)	k__Bacteria;p__Actinobacteria;c__Actinobacteria;o__Actinomycetales;f__Microbacteriaceae;g__Microbacterium;s__	0,99
bc05_G08;OTUwell_1	Bacteria(100.0),"Bacteroidetes"(100.0),"Sphingobacteria"(99.6),"Sphingobacteriales"(99.6),Chitinophagaceae(99.6),Filimonas(99.0)	k__Bacteria;p__Bacteroidetes;c__[Saprospirae];o__[Saprospirales];f__Chitinophagaceae;g__;s__	0,98
bc05_G09;OTUwell_1	Bacteria(100.0),"Proteobacteria"(100.0),Alphaproteobacteria(98.4),Rhizobiales(98.4),Xanthobacteraceae(97.4),Starkeya(91.7)	k__Bacteria;p__Proteobacteria;c__Alphaproteobacteria;o__Rhizobiales;f__Xanthobacteraceae;g__;s__	1,00
bc05_G10;OTUwell_1	Bacteria(100.0),"Proteobacteria"(100.0),Gammaproteobacteria(99.3),Xanthomonadales(99.3),Sinobacteraceae(99.3),Sinobacter(83.4)	k__Bacteria;p__Proteobacteria;c__Gammaproteobacteria;o__Xanthomonadales;f__Sinobacteraceae;g__;s__	1,00
bc05_G11;OTUwell_1	Bacteria(100.0),"Bacteroidetes"(100.0),"Sphingobacteria"(98.3),"Sphingobacteriales"(98.3),Chitinophagaceae(98.3),Filimonas(70.9)	k__Bacteria;p__Bacteroidetes;c__[Saprospirae];o__[Saprospirales];f__Chitinophagaceae;g__;s__	1,00
bc05_G12;OTUwell_1	Bacteria(100.0),"Proteobacteria"(100.0),Betaproteobacteria(97.1),Burkholderiales(97.1),Burkholderiales_incertae_sedis(95.2),Mitsuaria(88.0)	k__Bacteria;p__Proteobacteria;c__Betaproteobacteria;o__Burkholderiales;f__Comamonadaceae	1,00
bc05_H01;OTUwell_1	Bacteria(100.0),"Proteobacteria"(100.0),Alphaproteobacteria(97.9),Rhizobiales(97.9),Rhizobiaceae(97.8),Rhizobium(96.6)	k__Bacteria;p__Proteobacteria;c__Alphaproteobacteria;o__Rhizobiales;f__Rhizobiaceae;g__Rhizobium;s__	1,00
bc05_H01;OTUwell_2	Bacteria(100.0),"Bacteroidetes"(100.0),"Sphingobacteria"(97.8),"Sphingobacteriales"(97.8),Sphingobacteriaceae(97.8),Mucilaginibacter(94.5)	k__Bacteria;p__Bacteroidetes;c__Sphingobacteriia;o__Sphingobacteriales;f__Sphingobacteriaceae;g__;s__	1,00
bc05_H02;OTUwell_1	Bacteria(100.0),"Proteobacteria"(100.0),Alphaproteobacteria(99.6),Rhizobiales(99.5),Hyphomicrobiaceae(99.5),Hyphomicrobium(99.5)	k__Bacteria;p__Proteobacteria;c__Alphaproteobacteria;o__Rhizobiales;f__Hyphomicrobiaceae;g__Hyphomicrobium;s__	1,00
bc05_H02;OTUwell_2	Bacteria(100.0),"Proteobacteria"(100.0),Gammaproteobacteria(99.1),Xanthomonadales(99.1),Xanthomonadaceae(98.8),Fulvimonas(86.9)	k__Bacteria;p__Proteobacteria;c__Gammaproteobacteria;o__Xanthomonadales;f__Xanthomonadaceae;g__Dyella;s__	0,96
bc05_H02;OTUwell_3	Bacteria(100.0),"Proteobacteria"(100.0),Gammaproteobacteria(99.3),Xanthomonadales(99.2),Xanthomonadaceae(99.0),Fulvimonas(89.1)	k__Bacteria;p__Proteobacteria;c__Gammaproteobacteria;o__Xanthomonadales;f__Xanthomonadaceae;g__Dyella;s__	0,96
bc05_H03;OTUwell_1	Bacteria(100.0),"Bacteroidetes"(100.0),"Sphingobacteria"(100.0),"Sphingobacteriales"(100.0),Chitinophagaceae(100.0),Chitinophaga(99.9)	k__Bacteria;p__Bacteroidetes;c__[Saprospirae];o__[Saprospirales];f__Chitinophagaceae;g__Chitinophaga;s__	1,00
bc05_H03;OTUwell_2	Bacteria(100.0),"Bacteroidetes"(100.0),"Sphingobacteria"(97.4),"Sphingobacteriales"(97.4),Sphingobacteriaceae(97.4),Mucilaginibacter(92.4)	k__Bacteria;p__Bacteroidetes;c__Sphingobacteriia;o__Sphingobacteriales;f__Sphingobacteriaceae;g__;s__	1,00
bc05_H04;OTUwell_1	Bacteria(100.0),"Bacteroidetes"(100.0),"Sphingobacteria"(98.0),"Sphingobacteriales"(98.0),Sphingobacteriaceae(98.0),Mucilaginibacter(95.4)	k__Bacteria;p__Bacteroidetes;c__Sphingobacteriia;o__Sphingobacteriales;f__Sphingobacteriaceae;g__;s__	1,00
bc05_H05;OTUwell_1	Bacteria(100.0),"Bacteroidetes"(100.0),"Sphingobacteria"(99.6),"Sphingobacteriales"(99.6),Chitinophagaceae(99.6),Chitinophaga(99.0)	k__Bacteria;p__Bacteroidetes;c__[Saprospirae];o__[Saprospirales];f__Chitinophagaceae;g__Chitinophaga;s__	1,00
bc05_H05;OTUwell_2	Bacteria(100.0),"Bacteroidetes"(100.0),"Sphingobacteria"(98.2),"Sphingobacteriales"(98.2),Sphingobacteriaceae(98.2),Mucilaginibacter(95.9)	k__Bacteria;p__Bacteroidetes;c__Sphingobacteriia;o__Sphingobacteriales;f__Sphingobacteriaceae;g__;s__	1,00
bc05_H05;OTUwell_3	Bacteria(100.0),"Proteobacteria"(100.0),Gammaproteobacteria(99.5),Xanthomonadales(99.4),Xanthomonadaceae(99.3),Dyella(92.4)	k__Bacteria;p__Proteobacteria;c__Gammaproteobacteria;o__Xanthomonadales;f__Xanthomonadaceae;g__Dyella;s__	1,00
bc05_H06;OTUwell_1	Bacteria(100.0),"Proteobacteria"(100.0),Gammaproteobacteria(99.5),Xanthomonadales(99.3),Xanthomonadaceae(99.3),Fulvimonas(90.8)	k__Bacteria;p__Proteobacteria;c__Gammaproteobacteria;o__Xanthomonadales;f__Xanthomonadaceae;g__Dyella;s__	0,98
bc05_H06;OTUwell_2	Bacteria(100.0),"Bacteroidetes"(100.0),"Sphingobacteria"(99.3),"Sphingobacteriales"(99.3),Sphingobacteriaceae(99.3),Mucilaginibacter(99.3)	k__Bacteria;p__Bacteroidetes;c__Sphingobacteriia;o__Sphingobacteriales;f__Sphingobacteriaceae;g__;s__	1,00
bc05_H07;OTUwell_1	Bacteria(100.0),"Proteobacteria"(100.0),Alphaproteobacteria(99.6),Rhizobiales(99.5),Bradyrhizobiaceae(99.5),Bosea(99.5)	k__Bacteria;p__Proteobacteria;c__Alphaproteobacteria;o__Rhizobiales	1,00
bc05_H07;OTUwell_2	Bacteria(100.0),"Proteobacteria"(100.0),Betaproteobacteria(99.0),Burkholderiales(99.0),Burkholderiaceae(98.8),Burkholderia(97.7)	k__Bacteria;p__Proteobacteria;c__Betaproteobacteria;o__Burkholderiales;f__Burkholderiaceae	1,00
bc05_H09;OTUwell_1	Bacteria(100.0),"Proteobacteria"(100.0),Alphaproteobacteria(98.2),Rhizobiales(98.2),Rhizobiaceae(98.2),Rhizobium(98.0)	k__Bacteria;p__Proteobacteria;c__Alphaproteobacteria;o__Rhizobiales;f__Rhizobiaceae;g__Rhizobium;s__	0,97
bc05_H09;OTUwell_2	Bacteria(100.0),"Proteobacteria"(100.0),Gammaproteobacteria(99.5),Xanthomonadales(99.3),Xanthomonadaceae(99.1),Dyella(91.7)	k__Bacteria;p__Proteobacteria;c__Gammaproteobacteria;o__Xanthomonadales;f__Xanthomonadaceae;g__Dyella;s__	0,99
bc05_H09;OTUwell_3	Bacteria(100.0),"Proteobacteria"(100.0),Alphaproteobacteria(98.4),Rhizobiales(98.4),Rhizobiaceae(98.4),Rhizobium(98.4)	k__Bacteria;p__Proteobacteria;c__Alphaproteobacteria;o__Rhizobiales;f__Rhizobiaceae;g__Rhizobium;s__	0,88
bc05_H10;OTUwell_1	Bacteria(100.0),"Proteobacteria"(100.0),Gammaproteobacteria(100.0),"Enterobacteriales"(99.4),Enterobacteriaceae(99.4),Escherichia/Shigella(98.9)	k__Bacteria;p__Proteobacteria;c__Gammaproteobacteria;o__Enterobacteriales;f__Enterobacteriaceae;g__;s__	1,00
bc05_H11;OTUwell_1	Bacteria(100.0),"Proteobacteria"(100.0),Gammaproteobacteria(100.0),"Enterobacteriales"(99.5),Enterobacteriaceae(99.4),Escherichia/Shigella(99.0)	k__Bacteria;p__Proteobacteria;c__Gammaproteobacteria;o__Enterobacteriales;f__Enterobacteriaceae;g__;s__	1,00
bc05_H12;OTUwell_1	Bacteria(100.0),"Proteobacteria"(100.0),Gammaproteobacteria(100.0),"Enterobacteriales"(99.4),Enterobacteriaceae(99.4),Escherichia/Shigella(98.9)	k__Bacteria;p__Proteobacteria;c__Gammaproteobacteria;o__Enterobacteriales;f__Enterobacteriaceae;g__;s__	1,00

Supplementary Table 2. Genera with confidence score >0.95 as determined by the UTAX algorithm (www.drive5.com/usearch/manual/utax_algo.html) against a trained RDP[1] database (www.drive5.com/utax/rdp_16s.fa); number of representatives identified in each genus and number of representatives in each sampled organ of sugarcane.

Genera	Number of representatives	Representatives per tissues	
		Rhizosphere	Endophytic root
Chitinophaga	78	44	34
Arthrobacter	5	4	1
Asticcacaulis	3	1	2
Bacillus	90	68	22
Bosea	9	2	7
Burkholderia	52	37	15
Caulobacter	12	9	3
Curtobacterium	1	1	0
Dokdonella	4	1	3
Dyadobacter	5	2	3
Dyella	24	16	8
Ensifer	4	4	0
Enterobacter	11	6	5
Escherichia/Shigella	15	9	6
Filimonas	2	0	2
Flavobacterium	5	2	3
Hyphomicrobium	2	1	1
Inquilinus	8	4	4
Kaistia	2	1	1
Lysobacter	11	7	4
Mesorhizobium	1	0	1
Microbacterium	9	7	2
Mucilaginibacter	36	29	7
Mycobacterium	1	0	1
Paenibacillus	2	2	0
Pedobacter	12	9	3
Promicromonospora	2	1	1
Pseudomonas	6	3	3
Rhizobium	73	40	33
Sphingobium	1	0	1
Sphingomonas	2	0	2
Sporosarcina	3	1	2
Streptomyces	1	0	1
Variovorax	29	20	9

SUPPLEMENTARY TABLES REFERENCES

1. Cole, J. R. *et al*. The Ribosomal Database Project (RDP-II): Sequences and tools for high-throughput rRNA analysis. *Nucleic Acids Res*. **33,** 294–296 (2005).
2. DeSantis, T. Z. *et al*. Greengenes, a chimera-checked 16S rRNA gene database and workbench compatible with ARB. *Appl. Environ. Microbiol.* **72,** 5069–5072 (2006).
3. Wang, Q., Garrity, G. M., Tiedje, J. M. & Cole, J. R. Naïve bayesian classifier for rapid assignment of rRNA sequences into the new bacterial taxonomy. *Appl. Environ. Microbiol.* **73,** 5261–5267 (2007).

Multiplex amplicon sequencing for microbe identification in community-based culture collections

Jaderson Silveira Leite Armanhi[1,6], Rafael Soares Correa de Souza[1,6], Laura Migliorini de Araújo[1], Vagner Katsumi Okura[1], Piotr Mieczkowski[2], Juan Imperial[3,4] and Paulo Arruda[1,5*]

[1]Centro de Biologia Molecular e Engenharia Genética, Universidade Estadual de Campinas (UNICAMP), 13083-875, Campinas, SP, Brazil.

[2]Department of Genetics, University of North Carolina, Chapel Hill, North Carolina, USA.

[3]Centro de Biotecnología y Genómica de Plantas, Universidad Politécnica de Madrid (UPM) – Instituto Nacional de Investigación y Tecnología Agraria y Alimentaria (INIA), Campus Montegancedo UPM 28223 – Pozuelo de Alarcón (Madrid), Spain.

[4]Consejo Superior de Investigaciones Científicas, Madrid, Spain.

[5]Departamento de Genética e Evolução, Instituto de Biologia, Universidade Estadual de Campinas (UNICAMP), 13083-970, Campinas, SP, Brazil.

[6]These authors contributed equally to this work.

*Correspondence should be addressed to P.A. (parruda@unicamp.br).

Running title: Community-based culture collections

SUPPLEMENTARY DATA

Supplementary Data 1. Primer sequences used for library preparation in multiplex strategy.

Plate tagging (first PCR)

Forward primers

Primer_ID	Nextera transposase sequence	MT-FS	Linker	Forward rRNA primer	Complete sequence (5' to 3')
341f_MT	TCGTCGGCAGCGTCAGATGTGTATAAGAGACAG	NNNNNNNNN	TG	CCTACGGGRSGCAGCAG	TCGTCGGCAGCGTCAGATGTGTATAAGAGACAGNNNNNNNNNTGCCTACGGGRSGCAGCAG
008f_MT	TCGTCGGCAGCGTCAGATGTGTATAAGAGACAG	NNNNNNNNN	AC	AGAGTTTGATCMTGGC	TCGTCGGCAGCGTCAGATGTGTATAAGAGACAGNNNNNNNNNACAGAGTTTGATCMTGGC

Reverse primers (plate tagging)

Primer_ID	Nextera transposase sequence	MT-FS	Barcodes (plate tagging)	Linker	Reverse rRNA primer	Complete sequence (5' to 3')
1492r_MT_bc1	GTCTCGTGGGCTCGGAGATGTGTATAAGAGACAG	NNNN	TTACCGACG	CG	TACCTTGTTACGACTT	GTCTCGTGGGCTCGGAGATGTGTATAAGAGACAGNNNNTTACCGACGCGTACCTTGTTACGACTT
1492r_MT_bc2	GTCTCGTGGGCTCGGAGATGTGTATAAGAGACAG	NNNN	ATTGGACAC	CG	TACCTTGTTACGACTT	GTCTCGTGGGCTCGGAGATGTGTATAAGAGACAGNNNNATTGGACACCGTACCTTGTTACGACTT
1492r_MT_bc3	GTCTCGTGGGCTCGGAGATGTGTATAAGAGACAG	NNNN	TCGCATGGA	CG	TACCTTGTTACGACTT	GTCTCGTGGGCTCGGAGATGTGTATAAGAGACAGNNNNTCGCATGGACGTACCTTGTTACGACTT
1492r_MT_bc4	GTCTCGTGGGCTCGGAGATGTGTATAAGAGACAG	NNNN	AGCGAACCT	CG	TACCTTGTTACGACTT	GTCTCGTGGGCTCGGAGATGTGTATAAGAGACAGNNNNAGCGAACCTCGTACCTTGTTACGACTT
1492r_MT_bc5	GTCTCGTGGGCTCGGAGATGTGTATAAGAGACAG	NNNN	AGCTTCGAC	CG	TACCTTGTTACGACTT	GTCTCGTGGGCTCGGAGATGTGTATAAGAGACAGNNNNAGCTTCGACCGTACCTTGTTACGACTT
1492r_MT_bc6	GTCTCGTGGGCTCGGAGATGTGTATAAGAGACAG	NNNN	GTCAGCCGT	CG	TACCTTGTTACGACTT	GTCTCGTGGGCTCGGAGATGTGTATAAGAGACAGNNNNGTCAGCCGTCGTACCTTGTTACGACTT
1492r_MT_bc7	GTCTCGTGGGCTCGGAGATGTGTATAAGAGACAG	NNNN	TCCAGATAG	CG	TACCTTGTTACGACTT	GTCTCGTGGGCTCGGAGATGTGTATAAGAGACAGNNNNTCCAGATAGCGTACCTTGTTACGACTT
1492r_MT_bc8	GTCTCGTGGGCTCGGAGATGTGTATAAGAGACAG	NNNN	GAGAGTCCA	CG	TACCTTGTTACGACTT	GTCTCGTGGGCTCGGAGATGTGTATAAGAGACAGNNNNGAGAGTCCACGTACCTTGTTACGACTT
1492r_MT_bc9	GTCTCGTGGGCTCGGAGATGTGTATAAGAGACAG	NNNN	GCTCACAAT	CG	TACCTTGTTACGACTT	GTCTCGTGGGCTCGGAGATGTGTATAAGAGACAGNNNNGCTCACAATCGTACCTTGTTACGACTT
1492r_MT_bc10	GTCTCGTGGGCTCGGAGATGTGTATAAGAGACAG	NNNN	TTGACGACA	CG	TACCTTGTTACGACTT	GTCTCGTGGGCTCGGAGATGTGTATAAGAGACAGNNNNTTGACGACACGTACCTTGTTACGACTT
1492r_MT_bc11	GTCTCGTGGGCTCGGAGATGTGTATAAGAGACAG	NNNN	CTTAGAACG	CG	TACCTTGTTACGACTT	GTCTCGTGGGCTCGGAGATGTGTATAAGAGACAGNNNNCTTAGAACGCGTACCTTGTTACGACTT
1492r_MT_bc12	GTCTCGTGGGCTCGGAGATGTGTATAAGAGACAG	NNNN	CGGTTCACA	CG	TACCTTGTTACGACTT	GTCTCGTGGGCTCGGAGATGTGTATAAGAGACAGNNNNCGGTTCACACGTACCTTGTTACGACTT
1492r_MT_bc13	GTCTCGTGGGCTCGGAGATGTGTATAAGAGACAG	NNNN	CGATAGGCC	CG	TACCTTGTTACGACTT	GTCTCGTGGGCTCGGAGATGTGTATAAGAGACAGNNNNCGATAGGCCCGTACCTTGTTACGACTT
1492r_MT_bc14	GTCTCGTGGGCTCGGAGATGTGTATAAGAGACAG	NNNN	GCTATATCC	CG	TACCTTGTTACGACTT	GTCTCGTGGGCTCGGAGATGTGTATAAGAGACAGNNNNGCTATATCCCGTACCTTGTTACGACTT
1492r_MT_bc15	GTCTCGTGGGCTCGGAGATGTGTATAAGAGACAG	NNNN	GTCTTCAGC	CG	TACCTTGTTACGACTT	GTCTCGTGGGCTCGGAGATGTGTATAAGAGACAGNNNNGTCTTCAGCCGTACCTTGTTACGACTT
1492r_MT_bc16	GTCTCGTGGGCTCGGAGATGTGTATAAGAGACAG	NNNN	TAGACACCG	CG	TACCTTGTTACGACTT	GTCTCGTGGGCTCGGAGATGTGTATAAGAGACAGNNNNTAGACACCGCGTACCTTGTTACGACTT
1492r_MT_bc17	GTCTCGTGGGCTCGGAGATGTGTATAAGAGACAG	NNNN	TCAGCTGAC	CG	TACCTTGTTACGACTT	GTCTCGTGGGCTCGGAGATGTGTATAAGAGACAGNNNNTCAGCTGACCGTACCTTGTTACGACTT
1492r_MT_bc18	GTCTCGTGGGCTCGGAGATGTGTATAAGAGACAG	NNNN	TAAGTCGGC	CG	TACCTTGTTACGACTT	GTCTCGTGGGCTCGGAGATGTGTATAAGAGACAGNNNNTAAGTCGGCCGTACCTTGTTACGACTT
1492r_MT_bc19	GTCTCGTGGGCTCGGAGATGTGTATAAGAGACAG	NNNN	GCTCCTTAG	CG	TACCTTGTTACGACTT	GTCTCGTGGGCTCGGAGATGTGTATAAGAGACAGNNNNGCTCCTTAGCGTACCTTGTTACGACTT
1492r_MT_bc20	GTCTCGTGGGCTCGGAGATGTGTATAAGAGACAG	NNNN	ATGGCCTGA	CG	TACCTTGTTACGACTT	GTCTCGTGGGCTCGGAGATGTGTATAAGAGACAGNNNNATGGCCTGACGTACCTTGTTACGACTT
1492r_MT_bc21	GTCTCGTGGGCTCGGAGATGTGTATAAGAGACAG	NNNN	TTGCAAGTA	CG	TACCTTGTTACGACTT	GTCTCGTGGGCTCGGAGATGTGTATAAGAGACAGNNNNTTGCAAGTACGTACCTTGTTACGACTT
1492r_MT_bc22	GTCTCGTGGGCTCGGAGATGTGTATAAGAGACAG	NNNN	CCTAGTAAG	CG	TACCTTGTTACGACTT	GTCTCGTGGGCTCGGAGATGTGTATAAGAGACAGNNNNCCTAGTAAGCGTACCTTGTTACGACTT
1492r_MT_bc23	GTCTCGTGGGCTCGGAGATGTGTATAAGAGACAG	NNNN	CTAGGATCA	CG	TACCTTGTTACGACTT	GTCTCGTGGGCTCGGAGATGTGTATAAGAGACAGNNNNCTAGGATCACGTACCTTGTTACGACTT
1492r_MT_bc24	GTCTCGTGGGCTCGGAGATGTGTATAAGAGACAG	NNNN	TATGAACGT	CG	TACCTTGTTACGACTT	GTCTCGTGGGCTCGGAGATGTGTATAAGAGACAGNNNNTATGAACGTCGTACCTTGTTACGACTT
1492r_MT_bc25	GTCTCGTGGGCTCGGAGATGTGTATAAGAGACAG	NNNN	CTTGTGCGA	CG	TACCTTGTTACGACTT	GTCTCGTGGGCTCGGAGATGTGTATAAGAGACAGNNNNCTTGTGCGACGTACCTTGTTACGACTT
1492r_MT_bc26	GTCTCGTGGGCTCGGAGATGTGTATAAGAGACAG	NNNN	CACGATGGT	CG	TACCTTGTTACGACTT	GTCTCGTGGGCTCGGAGATGTGTATAAGAGACAGNNNNCACGATGGTCGTACCTTGTTACGACTT
1492r_MT_bc27	GTCTCGTGGGCTCGGAGATGTGTATAAGAGACAG	NNNN	ACGTGCCTT	CG	TACCTTGTTACGACTT	GTCTCGTGGGCTCGGAGATGTGTATAAGAGACAGNNNNACGTGCCTTCGTACCTTGTTACGACTT
1492r_MT_bc28	GTCTCGTGGGCTCGGAGATGTGTATAAGAGACAG	NNNN	TGAACTAGC	CG	TACCTTGTTACGACTT	GTCTCGTGGGCTCGGAGATGTGTATAAGAGACAGNNNNTGAACTAGCCGTACCTTGTTACGACTT
1492r_MT_bc29	GTCTCGTGGGCTCGGAGATGTGTATAAGAGACAG	NNNN	TATTCAGCG	CG	TACCTTGTTACGACTT	GTCTCGTGGGCTCGGAGATGTGTATAAGAGACAGNNNNTATTCAGCGCGTACCTTGTTACGACTT
1492r_MT_bc30	GTCTCGTGGGCTCGGAGATGTGTATAAGAGACAG	NNNN	TAATCGGTG	CG	TACCTTGTTACGACTT	GTCTCGTGGGCTCGGAGATGTGTATAAGAGACAGNNNNTAATCGGTGCGTACCTTGTTACGACTT
1492r_MT_bc31	GTCTCGTGGGCTCGGAGATGTGTATAAGAGACAG	NNNN	GCGTCCATG	CG	TACCTTGTTACGACTT	GTCTCGTGGGCTCGGAGATGTGTATAAGAGACAGNNNNGCGTCCATGCGTACCTTGTTACGACTT
1492r_MT_bc32	GTCTCGTGGGCTCGGAGATGTGTATAAGAGACAG	NNNN	CGTAAGATG	CG	TACCTTGTTACGACTT	GTCTCGTGGGCTCGGAGATGTGTATAAGAGACAGNNNNCGTAAGATGCGTACCTTGTTACGACTT
1492r_MT_bc33	GTCTCGTGGGCTCGGAGATGTGTATAAGAGACAG	NNNN	CTGTTACAG	CG	TACCTTGTTACGACTT	GTCTCGTGGGCTCGGAGATGTGTATAAGAGACAGNNNNCTGTTACAGCGTACCTTGTTACGACTT
1492r_MT_bc34	GTCTCGTGGGCTCGGAGATGTGTATAAGAGACAG	NNNN	ACGATCATC	CG	TACCTTGTTACGACTT	GTCTCGTGGGCTCGGAGATGTGTATAAGAGACAGNNNNACGATCATCCGTACCTTGTTACGACTT
1492r_MT_bc35	GTCTCGTGGGCTCGGAGATGTGTATAAGAGACAG	NNNN	GTAACGGCT	CG	TACCTTGTTACGACTT	GTCTCGTGGGCTCGGAGATGTGTATAAGAGACAGNNNNGTAACGGCTCGTACCTTGTTACGACTT
1492r_MT_bc36	GTCTCGTGGGCTCGGAGATGTGTATAAGAGACAG	NNNN	CCATGCTTA	CG	TACCTTGTTACGACTT	GTCTCGTGGGCTCGGAGATGTGTATAAGAGACAGNNNNCCATGCTTACGTACCTTGTTACGACTT
1492r_MT_bc37	GTCTCGTGGGCTCGGAGATGTGTATAAGAGACAG	NNNN	GTACGCACA	CG	TACCTTGTTACGACTT	GTCTCGTGGGCTCGGAGATGTGTATAAGAGACAGNNNNGTACGCACACGTACCTTGTTACGACTT
1492r_MT_bc38	GTCTCGTGGGCTCGGAGATGTGTATAAGAGACAG	NNNN	TTAGAGCCA	CG	TACCTTGTTACGACTT	GTCTCGTGGGCTCGGAGATGTGTATAAGAGACAGNNNNTTAGAGCCACGTACCTTGTTACGACTT

1492r_MT_bc39	GTCTCGTGGGCTCGGAGATGTGTATAAGAGACAG	NNNN	ATAAGGTCG	CG	TACCTTGTTACGACTT	GTCTCGTGGGCTCGGAGATGTGTATAAGAGACAGNNNNATAAGGTCGCGTACCTTGTTACGACTT
1492r_MT_bc40	GTCTCGTGGGCTCGGAGATGTGTATAAGAGACAG	NNNN	AGTGGCACT	CG	TACCTTGTTACGACTT	GTCTCGTGGGCTCGGAGATGTGTATAAGAGACAGNNNNAGTGGCACTCGTACCTTGTTACGACTT
1492r_MT_bc41	GTCTCGTGGGCTCGGAGATGTGTATAAGAGACAG	NNNN	CCAGAAGTG	CG	TACCTTGTTACGACTT	GTCTCGTGGGCTCGGAGATGTGTATAAGAGACAGNNNNCCAGAAGTGCGTACCTTGTTACGACTT
1492r_MT_bc42	GTCTCGTGGGCTCGGAGATGTGTATAAGAGACAG	NNNN	CTACTAGCG	CG	TACCTTGTTACGACTT	GTCTCGTGGGCTCGGAGATGTGTATAAGAGACAGNNNNCTACTAGCGCGTACCTTGTTACGACTT
1492r_MT_bc43	GTCTCGTGGGCTCGGAGATGTGTATAAGAGACAG	NNNN	TAGCGTTCC	CG	TACCTTGTTACGACTT	GTCTCGTGGGCTCGGAGATGTGTATAAGAGACAGNNNNTAGCGTTCCCGTACCTTGTTACGACTT
1492r_MT_bc44	GTCTCGTGGGCTCGGAGATGTGTATAAGAGACAG	NNNN	GTGAGTCAT	CG	TACCTTGTTACGACTT	GTCTCGTGGGCTCGGAGATGTGTATAAGAGACAGNNNNGTGAGTCATCGTACCTTGTTACGACTT
1492r_MT_bc45	GTCTCGTGGGCTCGGAGATGTGTATAAGAGACAG	NNNN	TGGTCCTAC	CG	TACCTTGTTACGACTT	GTCTCGTGGGCTCGGAGATGTGTATAAGAGACAGNNNNTGGTCCTACCGTACCTTGTTACGACTT
1492r_MT_bc46	GTCTCGTGGGCTCGGAGATGTGTATAAGAGACAG	NNNN	TACGCGTAC	CG	TACCTTGTTACGACTT	GTCTCGTGGGCTCGGAGATGTGTATAAGAGACAGNNNNTACGCGTACCGTACCTTGTTACGACTT
1492r_MT_bc47	GTCTCGTGGGCTCGGAGATGTGTATAAGAGACAG	NNNN	GAGCCATCT	CG	TACCTTGTTACGACTT	GTCTCGTGGGCTCGGAGATGTGTATAAGAGACAGNNNNGAGCCATCTCGTACCTTGTTACGACTT
1492r_MT_bc48	GTCTCGTGGGCTCGGAGATGTGTATAAGAGACAG	NNNN	CGTCCGTAT	CG	TACCTTGTTACGACTT	GTCTCGTGGGCTCGGAGATGTGTATAAGAGACAGNNNNCGTCCGTATCGTACCTTGTTACGACTT
1492r_MT_bc49	GTCTCGTGGGCTCGGAGATGTGTATAAGAGACAG	NNNN	GATACGTTC	CG	TACCTTGTTACGACTT	GTCTCGTGGGCTCGGAGATGTGTATAAGAGACAGNNNNGATACGTTCCGTACCTTGTTACGACTT
1492r_MT_bc50	GTCTCGTGGGCTCGGAGATGTGTATAAGAGACAG	NNNN	CAGCTGGTT	CG	TACCTTGTTACGACTT	GTCTCGTGGGCTCGGAGATGTGTATAAGAGACAGNNNNCAGCTGGTTCGTACCTTGTTACGACTT
1492r_MT_bc51	GTCTCGTGGGCTCGGAGATGTGTATAAGAGACAG	NNNN	TTAAGCGCC	CG	TACCTTGTTACGACTT	GTCTCGTGGGCTCGGAGATGTGTATAAGAGACAGNNNNTTAAGCGCCCGTACCTTGTTACGACTT
1492r_MT_bc52	GTCTCGTGGGCTCGGAGATGTGTATAAGAGACAG	NNNN	CCTGCGAAG	CG	TACCTTGTTACGACTT	GTCTCGTGGGCTCGGAGATGTGTATAAGAGACAGNNNNCCTGCGAAGCGTACCTTGTTACGACTT
1492r_MT_bc53	GTCTCGTGGGCTCGGAGATGTGTATAAGAGACAG	NNNN	TTGTAGCCG	CG	TACCTTGTTACGACTT	GTCTCGTGGGCTCGGAGATGTGTATAAGAGACAGNNNNTTGTAGCCGCGTACCTTGTTACGACTT
1492r_MT_bc54	GTCTCGTGGGCTCGGAGATGTGTATAAGAGACAG	NNNN	TCTGTAGAG	CG	TACCTTGTTACGACTT	GTCTCGTGGGCTCGGAGATGTGTATAAGAGACAGNNNNTCTGTAGAGCGTACCTTGTTACGACTT
1492r_MT_bc55	GTCTCGTGGGCTCGGAGATGTGTATAAGAGACAG	NNNN	CTATTAAGC	CG	TACCTTGTTACGACTT	GTCTCGTGGGCTCGGAGATGTGTATAAGAGACAGNNNNCTATTAAGCCGTACCTTGTTACGACTT
1492r_MT_bc56	GTCTCGTGGGCTCGGAGATGTGTATAAGAGACAG	NNNN	CTCTGAGGT	CG	TACCTTGTTACGACTT	GTCTCGTGGGCTCGGAGATGTGTATAAGAGACAGNNNNCTCTGAGGTCGTACCTTGTTACGACTT

Well tagging (second PCR)

Forward primers (row tagging)

Primer_ID	Forward Illumina adapter	Barcode (row tagging)	Nextera transposase sequence	Complete sequence (5' to 3')
S501	AATGATACGGCGACCACCGAGATCTACAC	TAGATCGC	TCGTCGGCAGCGTC	AATGATACGGCGACCACCGAGATCTACACTAGATCGCTCGTCGGCAGCGTC
S502	AATGATACGGCGACCACCGAGATCTACAC	CTCTCTAT	TCGTCGGCAGCGTC	AATGATACGGCGACCACCGAGATCTACACCTCTCTATTCGTCGGCAGCGTC
S503	AATGATACGGCGACCACCGAGATCTACAC	TATCCTCT	TCGTCGGCAGCGTC	AATGATACGGCGACCACCGAGATCTACACTATCCTCTTCGTCGGCAGCGTC
S504	AATGATACGGCGACCACCGAGATCTACAC	AGAGTAGA	TCGTCGGCAGCGTC	AATGATACGGCGACCACCGAGATCTACACAGAGTAGATCGTCGGCAGCGTC
S505	AATGATACGGCGACCACCGAGATCTACAC	GTAAGGAG	TCGTCGGCAGCGTC	AATGATACGGCGACCACCGAGATCTACACGTAAGGAGTCGTCGGCAGCGTC
S506	AATGATACGGCGACCACCGAGATCTACAC	ACTGCATA	TCGTCGGCAGCGTC	AATGATACGGCGACCACCGAGATCTACACACTGCATATCGTCGGCAGCGTC
S507	AATGATACGGCGACCACCGAGATCTACAC	AAGGAGTA	TCGTCGGCAGCGTC	AATGATACGGCGACCACCGAGATCTACACAAGGAGTATCGTCGGCAGCGTC
S508	AATGATACGGCGACCACCGAGATCTACAC	CTAAGCCT	TCGTCGGCAGCGTC	AATGATACGGCGACCACCGAGATCTACACCTAAGCCTTCGTCGGCAGCGTC

Reverse primers (column tagging)

Primer_ID	Reverse Illumina adapter	Barcode (column tagging)	Nextera transposase sequence	Complete sequence (5' to 3')
N701	CAAGCAGAAGACGGCATACGAGAT	TCGCCTTA	GTCTCGTGGGCTCGG	CAAGCAGAAGACGGCATACGAGATTCGCCTTAGTCTCGTGGGCTCGG
N702	CAAGCAGAAGACGGCATACGAGAT	CTAGTACG	GTCTCGTGGGCTCGG	CAAGCAGAAGACGGCATACGAGATCTAGTACGGTCTCGTGGGCTCGG
N703	CAAGCAGAAGACGGCATACGAGAT	TTCTGCCT	GTCTCGTGGGCTCGG	CAAGCAGAAGACGGCATACGAGATTTCTGCCTGTCTCGTGGGCTCGG
N704	CAAGCAGAAGACGGCATACGAGAT	GCTCAGGA	GTCTCGTGGGCTCGG	CAAGCAGAAGACGGCATACGAGATGCTCAGGAGTCTCGTGGGCTCGG
N705	CAAGCAGAAGACGGCATACGAGAT	AGGAGTCC	GTCTCGTGGGCTCGG	CAAGCAGAAGACGGCATACGAGATAGGAGTCCGTCTCGTGGGCTCGG
N706	CAAGCAGAAGACGGCATACGAGAT	CATGCCTA	GTCTCGTGGGCTCGG	CAAGCAGAAGACGGCATACGAGATCATGCCTAGTCTCGTGGGCTCGG
N707	CAAGCAGAAGACGGCATACGAGAT	GTAGAGAG	GTCTCGTGGGCTCGG	CAAGCAGAAGACGGCATACGAGATGTAGAGAGGTCTCGTGGGCTCGG
N708	CAAGCAGAAGACGGCATACGAGAT	CCTCTCTG	GTCTCGTGGGCTCGG	CAAGCAGAAGACGGCATACGAGATCCTCTCTGGTCTCGTGGGCTCGG
N709	CAAGCAGAAGACGGCATACGAGAT	AGCGTAGC	GTCTCGTGGGCTCGG	CAAGCAGAAGACGGCATACGAGATAGCGTAGCGTCTCGTGGGCTCGG
N710	CAAGCAGAAGACGGCATACGAGAT	CAGCCTCG	GTCTCGTGGGCTCGG	CAAGCAGAAGACGGCATACGAGATCAGCCTCGGTCTCGTGGGCTCGG
N711	CAAGCAGAAGACGGCATACGAGAT	TGCCTCTT	GTCTCGTGGGCTCGG	CAAGCAGAAGACGGCATACGAGATTGCCTCTTGTCTCGTGGGCTCGG
N712	CAAGCAGAAGACGGCATACGAGAT	TCCTCTAC	GTCTCGTGGGCTCGG	CAAGCAGAAGACGGCATACGAGATTCCTCTACGTCTCGTGGGCTCGG

Multiplex amplicon sequencing for microbe identification in community-based culture collections

Jaderson Silveira Leite Armanhi[1,6], Rafael Soares Correa de Souza[1,6], Laura Migliorini de Araújo[1], Vagner Katsumi Okura[1], Piotr Mieczkowski[2], Juan Imperial[3,4] and Paulo Arruda[1,5*]

[1]Centro de Biologia Molecular e Engenharia Genética, Universidade Estadual de Campinas (UNICAMP), 13083-875, Campinas, SP, Brazil.

[2]Department of Genetics, University of North Carolina, Chapel Hill, North Carolina, USA.

[3]Centro de Biotecnología y Genómica de Plantas, Universidad Politécnica de Madrid (UPM) – Instituto Nacional de Investigación y Tecnología Agraria y Alimentaria (INIA), Campus Montegancedo UPM 28223 – Pozuelo de Alarcón (Madrid), Spain.

[4]Consejo Superior de Investigaciones Científicas, Madrid, Spain.

[5]Departamento de Genética e Evolução, Instituto de Biologia, Universidade Estadual de Campinas (UNICAMP), 13083-970, Campinas, SP, Brazil.

[6]These authors contributed equally to this work.

*Correspondence should be addressed to P.A. (parruda@unicamp.br).

Running title: Community-based culture collections

SUPPLEMENTARY NOTE

SUPPLEMENTARY NOTE

Community analysis of Sugarcane Microbiome. The microbial community composition of sugarcane was accessed through amplicon sequencing of 16S V4 region using the HiSeq 2500 platform[1]. A total of 341 million reads from roots, stalks, top leaves and young shoots were sampled from plants harvested at the 4, 6, 8 and 10th months after budding. Microbiota community was accessed in the internal (endophytic) and external (exophytic) organs. Clustering using UPARSE[2] pipeline resulted in 23,811 prokaryotic operational taxonomic units (OTUs). A customized dataset was prepared by selecting for OTUs with at least one hit against Greengenes[3] rRNA 16S gene database at ≥75% identity using the program "usearch_global" from USEARCH[4] software package. Then, based on the number of reads of the remaining 20,731 OTUs, the relative abundances for each OTU were recalculated per sample.

ADDITIONAL DETAILS OF THE FIGURES

Figure 2a. *Heatmap*. Heatmap was constructed by counting the matches in a pair-wise alignment of CCSs using 97% identity as a threshold. The CCS coverage was considered to evaluate the correlation between low- and high-coverage CCS. Taken into consideration that high-coverage CCS have a higher probability of representing true biological sequences, a match of low-coverage CCS against a high-coverage CCS would raise the confidence on that low-coverage CCS also to be a true biological sequence. It directly affects OTU clustering and related biological questions. For example, if an OTU is formed by the clustering of three CCSs with 3, 15 and 30× coverage, one could assume that even though one low-coverage CCS took part on clustering, the presence of other two high-coverage CCS would raise the reliability of that OTU to represent a true biological information. The fact that most low-coverage CCSs match high-coverage CCSs implies that this example would be the prevalent case in our dataset. The color assignment was made from the minimum to the maximum value of observations for each case of alignment. CCSs with no match, here called singletons, were also counted.

Figures 2b, 2c, Supplementary Figures 3a, 3b, 4b, 6b, 7 and 9. *Graphs*. All referred graphs were generated using GraphPad Prism v6 (GraphPad Software, www.graphpad.com).

Figure 2b, Supplementary Figure 6a and 6b. *Filtering of error-prone CCS*. The pipeline is outlined in **Supplementary Figure 6b**. The CCS dataset was aligned against Greengenes[3] database. CCSs with at least one hit against database were considered reliable and kept (light gray). Remaining CCS (without hits against the database) were then aligned against the CCS dataset. CCSs with at least one hit were considered reliable and also kept (strong gray). CCSs with no hit against database were considered error-prone and discarded (black). Both alignments were performed using the program "usearch_global" from USEARCH[4] software package with ≥97% identity. **Figure 2b** shows the percentage of CCSs for a given coverage. **Supplementary Figure 6b** shows raw numbers of the same above-mentioned analysis. The cumulative percentage of CCSs (triangles, in **Supplementary Figure 6b**) were calculated by dividing the amount of CCS considering a threshold of a given coverage by the total amount of CCSs.

Figure 2c. *Identification of more than one OTU per well in the community-based culture collection*. Wells that harbor a given number of microorganisms were counted based on the OTU table obtained from the second step of clustering.

Figure 3a, 3b and Supplementary Figure S8. *Cladograms*. OTUs obtained from the first step of clustering of the CBC were aligned to the sequences obtained through culture-independent community analysis using "usearch_global" from USEARCH[4] with ≥97% identity. We considered only the best hit for each OTU from the collection of microorganisms. The taxonomical assignment was transcribed from culture-independent data and used for the construction of the cladograms. Cladograms were constructed using GraPhlAn[5]. Each level of the cladogram represents a level of the taxonomic rank (from kingdom to genus). Due to the vast diversity of belowground compartments (**Figure 3a**), genera with no representatives in the collection were collapsed at class level (the complete cladogram is shown in **Supplementary Figure S8**). Relative abundances were calculated for each genus (or class, when collapsed) by taking together relative abundances of all representatives in the respective taxonomic level.

Figure 4. *Relative abundance of microorganisms from the collection in several sugarcane organs*. From the alignment obtained for the construction of the cladograms (**Figure 3a**, **3b** and **Supplementary Figure S8**), the relative abundance of each OTU, as well as their classification as belonging to the core communities, were transcribed from the customized culture-independent community profile of sugarcane root, leaf, and stalks[1].

Supplementary Figures S3a and S3b. *Estimative of the error rate of the CCSs based on* E. coli *16S rRNA gene*. A total of 200 CCSs from *E. coli* 16S V4–V9 region were aligned to the full-length 16S ribosomal RNA from rrnB operon of this organism deposited in GenBank (gene ID 948466), used as a reference. For the purpose of comparison, we decided to align the CCS of *E. coli* against only one of the seven ribosomal RNA found in the reference genome. Our choice was made upon the fact that these operons differ from each other in less than 1% in nucleotide sequence, as also reported in other researches[6]. Since traditional amplicon clustering uses thresholds above 3%, this would not be relevant for accuracy validation of 16S CCS amplicon and would serve as a good approximation. We used the program "usearch_global" from USEARCH[4] with ≥97% identity. The nucleotides were numbered according to the reference 16S rRNA gene. Insertions in CCS sequences were not considered. Mismatches or deletions were represented as a black dot (**Supplementary Figure S3a**). CCSs were grouped according to their coverage. For each group, the number of errors for a given nucleotide position was divided by the total number of sequences in the group and multiplied by 100 to generate the percentage of errors at each base (**Supplementary Figure S3b**). We noted that specific nucleotide positions (i.e. 349, 350, 449, 1,002, 1,010 and 1,006) showed remarkable variability in our data (high error rate). Our investigation has shown that these are the specific position in which the *E. coli* operons differ from each other. Thus, the higher error rates displayed in this figure resulted from the method of approximation in which CCSs were aligned regardless their operon of origin.

Supplementary Figure S7. *Redundancy of OTUs*. We used the OTU table obtained from the second step of clustering. This OTU table designates presence or absence of each OTU (rows) in each well (columns). The redundancy of OTUs was calculated by counting the number of wells that harbor a given OTU. OTUs were ordered (*x*-axis) from the most to the least abundant.

SUPPLEMENTARY NOTE REFERENCES

1. Souza, R. S. C. *et al*. Unlocking the bacterial and fungal communities assemblages of sugarcane microbiome. *Sci. Rep.* **6,** 28774 (2016).
2. Edgar, R. C. UPARSE: highly accurate OTU sequences from microbial amplicon reads. *Nat. Methods* **10,** 996–998 (2013).
3. DeSantis, T. Z. *et al*. Greengenes, a chimera-checked 16S rRNA gene database and workbench compatible with ARB. *Appl. Environ. Microbiol.* **72,** 5069–5072 (2006).
4. Edgar, R. C. Search and clustering orders of magnitude faster than BLAST. *Bioinformatics* **26,** 2460–2461 (2010).
5. Asnicar, F., Weingart, G., Tickle, T. L., Huttenhower, C. & Segata, N. Compact graphical representation of phylogenetic data and metadata with GraPhlAn. *PeerJ* **3,** e1029 (2015).
6. Pei, A. Y. *et al*. Diversity of 16S rRNA genes within individual prokaryotic genomes. *Appl. Environ. Microbiol.* **76,** 3886–3897 (2010).
